# Supplementary material for: Determining the burden of missed opportunities for vaccination among children admitted in healthcare facilities in India: a cross-sectional study
Source: BMJ Open. 2021 Mar 19;11(3):e046464. doi: 10.1136/bmjopen-2020-046464 (PMC7986782; doi:10.1136/bmjopen-2020-046464)
Supplement: Supplementary data [file bmjopen-2020-046464supp001.pdf]

Supplementary Data

Table S1. Characteristics of enrolled children, their caregivers, and the household (N = 263)

	n	(%)
**Child Age (Months)**		
Minimum	1	
Mean	13	
Median	7	
Maximum	58	
**Child Gender**		
Male	152	57.8%
Female	111	42.2%
**Child Order**		
First-born	119	45.2%
Second-born	87	33.1%
Third-born	36	13.7%
Fourth-born or younger	21	8.0%
**Caregiver Age (Years)**		
Minimum	18	
Mean	27	
Median	25	
Maximum	63	
**Caregiver Gender[1]**		
Male	56	21.4%
Female	206	78.6%
**Relationship to Child[2]**		
Mother	192	73.3%
Father	50	19.1%
Aunt / Uncle	11	4.2%
Grandparent	9	3.4%
**Can caregiver read and write**		
Yes	203	77.2%
No	60	22.8%
**Caregiver level of education[3]**		
No formal education / did not complete primary	58	22.1%
Primary / middle school	35	13.4%
High School	84	32.1%
College and postgraduate	85	32.4%
**Household Religion**		
Hindu	205	77.9%
Muslim	46	17.5%

Sikh	12	4.6%
**Primary Residence**		
Urban	86	32.7%
Rural	160	60.8%
Suburban	17	6.5%
**Household Total**		
Minimum	3	
Mean	8	
Median	7	
Maximum	26	
**Child been in contact with another health center before today**[4]		
Yes	138	53.7%
No	119	46.3%
**When was child admitted to the other health center**[5] (denominator includes those with prior health center contact - 138)		
Within last month	66	47.8%
1 – 6 months	45	32.6%
Over 6 months	27	19.6%
**Makes the primary decisions on vaccination in the household**[6]		
Mother	142	55.3%
Father	19	7.4%
Consensus of father and mother	72	28.0%
Grandparent or other relative	24	9.3%
**Distance of this hospital (PGIMER or SMS) from residence**[7]		
0-5 km	24	9.3%
6-10 km	20	7.8%
11-50 km	26	10.1%
>50 km	188	72.9%
**Distance of any health facility from residence**[8]		
0-5 km	177	68.9%
6-10 km	60	23.3%
11-50 km	18	7.0%
>50 km	2	0.8%
**Child has a vaccination card**		
Yes, present at hospital	154	58.6%
Yes, absent at hospital	89	33.8%
No	20	7.6%
**Source of Immunization History**		
Immunization card	157	59.7%
Parental recall	106	40.3%
**Immunization Status**		
Fully Vaccinated	91	34.6%
Partially Vaccinated	158	60.1%

Unvaccinated	14	5.3%

[1] 1 respondent missing

[2] 1 respondent missing

[3] 1 respondent missing

[4] 6 respondents missing

[5] Denominator is 138 children in contact with another health facilty before today

[6] 6 respondents missing

[7] 5 respondents missing

[8] 6 respondents missing

Table S2. Additional vaccine coverage (N = 263)

Vaccine Dose	Sample Immunization Status: Vaccinated N	Age Eligible N	Coverage (%)
f-IPV[1] Dose 1	108	263	41.1%
f-IPV[1] Dose 2	52	204	25.5%
Rotavirus Dose 1	74	263	28.1%
Rotavirus Dose 2	55	225	24.4%
Rotavirus Dose 3	35	204	17.2%
Measles 2 (Measles, MR[2], MMR[3])	18	44	40.9%
OPV[4] Booster	25	44	56.8%
DPT[5] Booster	26	44	59.1%

[1] Fractional dose of inactivated poliovirus vaccine (f-IPV).

[2] Measles -rubella (MR) vaccine.

[3] Measles-mumps-rubella (MMR) vaccine.

[4] Oral poliovirus vaccine (OPV).

[5] Diphtheria-tetanus-pertussis (DPT) vaccine.

Table S3. Full vaccination coverage by site compared to state coverage rates (Rajasthan and Chandigarh, NFHS-4)

Fully vaccinated Jaipur study site		Fully vaccinated (Jaipur District, NFHS-4)		Fully vaccinated Chandigarh study site		Fully vaccinated (Chandigarh, NFHS-4)	
n	%	n[1]	%[1,2]	n	%	n[1]	%[1,2]
66	32.8%	5582	58.2	25	40.3	29	79.5

1 International Institute for Population Sciences (IIPS) and ICF. National Family Health Survey (NFHS-4) 2015-16 [Dataset]. Data Extract from IAIR72.SAV, IAKR72.SAV, IABR72.SAV, IAMR74.SAV, and IAPR74.SAV. IPUMS Demographic and Health Surveys (IPUMS DHS), 2018. [2]
2 Ministries of Health and Family Welfare. National Family Health Survey - 4 District Fact Sheet Jaipur Rajasthan. 2015. http://rchiips.org/nfhs/FCTS/RJ/RJ_FactSheet_110_Jaipur.pdf [4]
3 Ministries of Health and Family Welfare. National Family Health Survey-4 State Fact Sheet Chandigarh. *Natl Fam Heal Surv – 4, Demogr Heal Surv* 2015. http://rchiips.org/nfhs/pdf/NFHS4/CH_FactSheet.pdf [5]
