# Supplementary material for: Determining the burden of missed opportunities for vaccination among children admitted in healthcare facilities in India: a cross-sectional study
Source: BMJ Open. 2021 Mar 19;11(3):e046464. doi: 10.1136/bmjopen-2020-046464 (PMC7986782; doi:10.1136/bmjopen-2020-046464)
Supplement: Author's manuscript [file bmjopen-2020-046464.draft_revisions.pdf]

BMJ Open is committed to open peer review. As part of this commitment we make the peer review history of every article we publish publicly available.

When an article is published we post the peer reviewers' comments and the authors' responses online. We also post the versions of the paper that were used during peer review. These are the versions that the peer review comments apply to.

The versions of the paper that follow are the versions that were submitted during the peer review process. They are not the versions of record or the final published versions. They should not be cited or distributed as the published version of this manuscript.

BMJ Open is an open access journal and the full, final, typeset and author-corrected version of record of the manuscript is available on our site with no access controls, subscription charges or pay-per-view fees (http://bmjopen.bmj.com).

If you have any questions on BMJ Open's open peer review process please email info.bmjopen@bmj.com

BMJ Open

Missed opportunities for vaccination among children admitted in healthcare facilities in India

Journal:	*BMJ Open*
Manuscript ID	bmjopen-2020-046464
Article Type:	Original research
Date Submitted by the Author:	03-Nov-2020
Complete List of Authors:	Albaugh, Nicholas; Johns Hopkins University Bloomberg School of Public Health, Department of Epidemiology<br>Matthew, Joseph; Post Graduate Institute of Medical Education and Research, Pediatrics<br>Choudhary, Richa ; Sawai Man Singh Medical College and Hospital, Pediatrics<br>Sitaraman, Sadasivan; Sawai Man Singh Medical College and Hospital, Pediatrics<br>Tomar, Anjali ; Sawai Man Singh Medical College and Hospital, Pediatrics<br>Bajwa, Ishumeet ; Post Graduate Institute of Medical Education and Research, Pediatrics<br>Dhaliwal, Baldeep; Johns Hopkins University Bloomberg School of Public Health, International Health<br>Shet, Anita; Johns Hopkins University Bloomberg School of Public Health, International Health
Keywords:	PAEDIATRICS, Paediatric infectious disease & immunisation < PAEDIATRICS, PUBLIC HEALTH

SCHOLARONE™
Manuscripts

I, the Submitting Author has the right to grant and does grant on behalf of all authors of the Work (as defined in the below author licence), an exclusive licence and/or a non-exclusive licence for contributions from authors who are: i) UK Crown employees; ii) where BMJ has agreed a CC-BY licence shall apply, and/or iii) in accordance with the terms applicable for US Federal Government officers or employees acting as part of their official duties; on a worldwide, perpetual, irrevocable, royalty-free basis to BMJ Publishing Group Ltd ("BMJ") its licensees and where the relevant Journal is co-owned by BMJ to the co-owners of the Journal, to publish the Work in this journal and any other BMJ products and to exploit all rights, as set out in our licence.

The Submitting Author accepts and understands that any supply made under these terms is made by BMJ to the Submitting Author unless you are acting as an employee on behalf of your employer or a postgraduate student of an affiliated institution which is paying any applicable article publishing charge ("APC") for Open Access articles. Where the Submitting Author wishes to make the Work available on an Open Access basis (and intends to pay the relevant APC), the terms of reuse of such Open Access shall be governed by a Creative Commons licence – details of these licences and which Creative Commons licence will apply to this Work are set out in our licence referred to above.

Other than as permitted in any relevant BMJ Author's Self Archiving Policies, I confirm this Work has not been accepted for publication elsewhere, is not being considered for publication elsewhere and does not duplicate material already published. I confirm all authors consent to publication of this Work and authorise the granting of this licence.

For peer review only

Missed opportunities for vaccination among children admitted in healthcare facilities in India

Nicholas W. Albaugh,[1,2] Joseph L. Mathew,[3] Richa Choudhary,[4] S. Sitaraman,[4] Anjali Tomar,[4] Ishumeet Kaur Bajwa,[3] Baldeep Dhaliwal,[2] Anita Shet[2,*]

1. Johns Hopkins Bloomberg School of Public Health, Department of Epidemiology, Baltimore, Maryland
2. Johns Hopkins Bloomberg School of Public Health, Department of International Health, Baltimore, Maryland
3. Postgraduate Institute of Medical Education and Research, Department of Pediatrics, Chandigarh, India
4. Sawai Man Singh Medical College, Department of Pediatrics, Jaipur, India

* Corresponding author at: Department of International Health, Johns Hopkins Bloomberg School of Public Health, 415 N. Washington Street, #535, Baltimore, MD 21205, USA.
Email: ashet1@jhu.edu

Word Count (excluding title page, abstract, references, figures and tables): 3,420
Tables: 3
Figures: 2
Supplementary Tables: 2

Keywords: vaccination, MOV, immunization status, India

Abbreviations:
aPR = Adjusted prevalence ratio
BCG = Bacille Calmette-Guerin vaccine
CI = Confidence interval
DPT = Diphtheria-tetanus-pertussis vaccine
f-IPV = Fractional dose of inactivated poliovirus vaccine
Hep B = Hepatitis B vaccine
MCV = Measles containing vaccine
MMR = Measles-mumps-rubella vaccine
MOV = Missed opportunity for vaccination
MR = Measles -rubella vaccine
OPV = Oral poliovirus vaccine
Penta = Diphtheria-tetanus-pertussis-hepatitis B-Haemophilus influenzae type b pentavalent (Penta) vaccine

For peer review only

ABSTRACT

Objectives

Children accessing healthcare systems represent a vulnerable population with risk factors for poor health outcomes, including vaccine-preventable diseases. We aimed to quantify missed vaccination opportunities among hospitalized children in India, and identify vaccination barriers perceived by caregivers and healthcare providers.

Setting

Two public-sector tertiary care hospitals in northern India, during November 2018 and March 2019.

Participants

We tracked 263 children aged 1-59 months who were incompletely vaccinated, until discharge, to document catch-up vaccinations given during the hospital stay, or vaccination advice or referals at discharge. We interviewed caregivers and healthcare providers to assess their perceptions on missed opportunities for vaccination.

Outcomes

Study outcomes included the proportion of hospitalized children considered under-vaccinated for their age; the proportion of missed opportunities for vaccination among under-vaccinated children who were eligible for vaccination, and vaccine coverage by antigen.

For peer review only

Results

We found that 65.4% of hospitalized children were under-vaccinated for their age when they presented to the hospital; among under-vaccinated children, 61.0% were less than 4 months old, and 55.6% reported prior contact with a health facility for a sick visit. The proportion of under-vaccinated children in hospitals were higher compared to the general population as indicated by regional vaccination coverage data. Among 161 children with inadequate vaccination who were tracked till discharge, 158 (98.1%) remained incompletely vaccinated at discharge and were considered 'missed opportunities for vaccination'. Inappropriate perception of vaccination contraindications was the most common reason for healthcare providers not to vaccinate children during hospital stay. Among caregivers of under-vaccinated children, 90.1% reported being comfortable having their children vaccinated while they were sick.

Conclusion

This pilot study confirmed that hospitalized sick children had substantial missed vaccination opportunities. This situation can be rectified through concerted actions involving caregivers and healthcare systems.

For peer review only

Strengths and limitations of this study

- This study focuses on children accessing healthcare facilities, and findings indicate that vaccination screening and catch-up vaccination at the inpatient health facility level provides an important opportunity to improve vaccination coverage and protection against vaccine-preventable diseases.
- Hospitalized children at the two regions in India were more likely to be under-vaccinated compared to the general population as indicated by regional vaccination coverage information, and a high proportion of hospitalized children were missed for catch-up vaccination during their admission and hospital course.
- Over half of the under-vaccinated children reported prior healthcare facility contact for a sick visit, indicating that these children were well within reach of the heathcare system.
- While this study is limited by its small size, the public-sector hospitals participating in this study are representative of healthcare facilities accessed by a majority of the nation’s population.

For peer review only

Introduction

Although global vaccination rates have remained high over for the last ten years[1], more than one out of every three children were not fully vaccinated in India in 2014[2]. According to the 2015-2016 National Family Health Survey (NFHS-4), vaccination rates for recommended vaccines in India's National Immunization Schedule have improved steadily in the past ten years by 8-23% [3-5]. A massive nationwide effort called the Mission Indradhanush strategy was launched by the Government of India in 2014, which vaccinated approximately 25.5 million vulnerable children in a two-year span [6]. Nonetheless, a joint report in 2016 conducted by the World Health Organization (WHO), United Nations, and Government of India Ministry of Health and Family Welfare concluded that estimated vaccination rates tended to be overestimated by administrative vaccination coverage reports and must still be improved to meet the WHO Sustainable Development Goal (SDG # 3) of reducing child mortality [7]. The report acknowledged the challenges faced by the government in identifying and addressing ‘missed vaccination’ opportunities for children [7]. WHO defines a missed opportunity for vaccination (MOV) as any contact with health services by a child or adult who is eligible for vaccination, which does not result in the individual receiving all the vaccine doses for which he or she is eligible [8].

Hospitalized children represent a vulnerable population with risk factors for poor health outcomes, who are more likely than the general population to be eligible for catch-up vaccinations [9]. Children admitted to healthcare facilities may be at greater risk for infectious diseases, and especially vaccine-preventable illnesses [10]. These children can benefit if vaccination opportunities are identified and addressed while the child is in the hospital. An

For peer review only

analysis using Demographic Health Surveys (DHS) and Multiple Indicator Cluster Surveys (MICS) estimated that if children in contact with health services for acute care were to receive their due vaccines, the potential increase in DTP3 vaccination coverage would be 3-14% in low and middle income countries (LMIC) settings, including a 12% increase in India [10].

While studies have attempted to quantify missed opportunities for vaccination by inpatient healthcare providers through questionnaires and interviews, the use of vaccination cards and medical records can provide more objective evidence of vaccination status. Our study aimed to document vaccination status of hospitalized children by verifying the medical records at hospital admission and discharge, and assess vaccination barriers perceived by caregivers and healthcare providers. Our findings will be useful for estimating the proportion of missed opportunities for vaccination among hospitalized children in India, and to understand local barriers for vaccination that can potentially form the basis for contextually effective interventions.

Methods

This study was a cross-sectional observational research study at two tertiary care government hospitals in Chandigarh and Jaipur conducted between November 2018 and March 2019. Children aged 1-59 months, were screened for study eligibility at admission to the hospitals. Those children admitted to the hospital, and whose parents agreed to provide vaccination information and consented to the study, were enrolled. Children who had health conditions that precluded the use of vaccines, such as a known primary or secondary immune deficiency, malignancy requiring chemotherapy, and children with previous reported allergies or contraindications to any vaccine or vaccine component, were excluded from enrolment.

For peer review only

Following written informed consent from the caregiver, concerted efforts were made to obtain the vaccination card from parents during the hospital stay. If the card was not present, the parent or caregiver was requested to have a family member bring the card from their home or send a picture of the card using their mobile phone. Parental recall was used to document vaccination status only if the vaccination card was unavailable.

At admission, caregivers were interviewed about demographic details, household information and prior health seeking behaviors. Children who had received all recommended vaccines for their age, per the prevalent national immunization schedule, were classified as 'fully vaccinated' [11]. Those who had received some, but not all, of the recommended vaccines for their age group, were considered 'partially vaccinated'. Those who had received none of the recommended vaccines for their age were labelled 'unvaccinated'. Research team members used study definitions and the recommended national immunization schedule to classify children who were unvaccinated or partially vaccinated as 'under-vaccinated'. This classification was verified during the analysis phase using the child's age at time of interview and reported vaccinations. No discrepancies existed between research staff evaluation and analysis. Under-vaccinated children were tracked until discharge to record if any vaccinations, or advice regarding vaccinations, had been given during hospitalization or at discharge. The medical chart was reviewed to document ~~hospitalization duration, diagnosis, illness severity,~~ which vaccines, if any, were administered during hospitalization. The caregivers of under-vaccinated children were interviewed at discharge to assess their perspectives on vaccination within the context of the hospital.

For peer review only

After the enrollment period, surveys were distributed to hospital healthcare providers involved in children's admission, treatment and discharge. Providers were asked to anonymously share their professional experiences, vaccination practices and related policies of the hospital. Eligible healthcare providers included physicians, nurses, pharmacy personnel, and postgraduate residents in the inpatient unit and the emergency unit. Questions were derived from the WHO *Planning Guide to Reduce Missed Opportunities for Vaccination* and WHO *Methodology for the Assessment of Missed Opportunities for Vaccination* [10, 12].

Ethical considerations

This study was approved by The Institutional Review Board at the Johns Hopkins Bloomberg School of Public Health and local Institutional Ethics Committees at the Postgraduate Institute of Medical Education and Research (PGIMER), Chandigarh and Sawai Man Singh (SMS) Medical College and attached hospitals, Jaipur. Informed consent was taken from participants prior to enrollment and implementation of study procedures. All anonymized data was collected on paper and entered into REDCap.

Patient and public involvement

Prior to study initiation and during the development process, some caregivers of hospitalized children were involved in helping with the refining and piloting of the questionnaires used in the study, and this contribution is noted in the acknowledgement section. Patients and their caregivers were active participants in this research, but were not involved in the conduct, analysis or dissemination of this research.

For peer review only

Statistical analysis

Study outcomes included (i) the proportion of hospitalized children considered under-vaccinated for their age; (ii) the proportion of missed opportunities for vaccination among under-vaccinated children who were eligible for vaccination [8] and (iii) vaccine coverage by antigen. In addition, we compared these estimates of vaccine coverage among our sample of hospitalized children with regional Chandigarh and Jaipur estimates from the NFHS-4 published dataset [2]. For this sub-analysis to remain comparable with the NFHS-4 datasets, we only included children aged 12-23 months old using the NFHS-4 definition of 'fully vaccinated' who have received 1 dose of BCG, 3 doses each of polio and DPT+Hib+ Hep B (Penta) and 1 dose measles [13]. We fitted multivariable Poisson regression models for predictors of being under-vaccinated. Poisson regression is more appropriate than logistic regression due to the common (>10%) prevalence of being under-vaccinated and because the log binomial odds ratio models did not converge [14]. Weight-for-age z-scores were calculated using WHO growth indicator equations [15]. Caregiver and healthcare provider survey responses were coded and analyzed as binary outcomes for knowledge questions (correct/incorrect) and perception statements (agree/disagree). All statistical analyses were conducted using Stata (Version 13.1, StataCorp, College Station, Texas, United States).

Results

Of the 312 admitted children who were screened for enrollment, 49 were ineligible (35 had no vaccination card or parental recall of vaccination details and 14 had a prior precluding health condition), leaving 263 (62 at Chandigarh and 201 at Jaipur) who consented and were subsequently enrolled into the study (Figure 1).

For peer review only

171

172 *Participant characteristics*

173 The median age of children in our sample was 7 months (range, 1 to 58 months), and 57.8% 174 (152/263) were male. First-born children made up 45.2% (119/263) of our sample. The median 175 age of caregivers in the study was 25 years (ranging from 18 to 63 years), and 78.6% (206/262) 176 were female, with the majority of caregivers being mothers. The highest attained education level 177 was high school or greater among 64.5% of caregivers, and 77.2% could read and write. The 178 primary residential setting was rural among 60.8% (160/263), with 31.1% (80/263) of children 179 living more than 5 kilometers away from their nearest health center (Supplemental Table 1). At 180 the time of this study, 92.4% (243/263) caregivers reported having a vaccination card for their 181 children, however only 59.7% (157/263) were able to make this available at the hospital. 182 Parental recall was used as the immunization source among the remaining 40.3% (106/263). 183 [Supplemental Table 1].

184

185 *Vaccination status*

186 Among the 263 children included in the final analysis, 34.6% (91) of the children were fully 187 vaccinated while 65.4% (172) were under-vaccinated. Among under-vaccinated children, 60.1% 188 (158/263) and 5.3% (14/263) were partially vaccinated and unvaccinated, respectively. The 189 proportion of under-vaccinated children was highest at 76.6%, (59/77) among those under four 190 months of age (Figure 2). The average hospital duration among those under-vaccinated was 5 191 days (ranging from 1 to 58 days), and 47% (72/161) were classified as underweight (weight < 2 192 z-scores below the mean age-for weight WHO definition) [15]. Prior health facility contact for a

For peer review only

sick visit at any time since birth, and within the last month, was reported in 55.6% (93/167) and 40.7% (68/167) of under-vaccinated children, respectively (Table 3).

The vaccinations with the lowest coverage among all included children were OPV3 (53.9%), DPT3 (54.9%), and DPT2 (63.1%). Our sample also had less than 80% vaccination coverage for the following vaccines: OPV0 (64.3%), OPV1 (69.2%), and OPV2 (64.4%) and MCV1 (79.7%). The coverage of Measles-containing-vaccine second-dose (MCV2) [Measles, MR, MMR], OPV booster dose and DPT booster dose were 40.9%, 56.8% and 59.1% respectively (Supplemental Table 2). Using the NHFS-4 study definition of full vaccination, our sample of 12-23 months old children had a proportion of 35.4% (17/48) fully vaccinated children, while the reported 2015-2016 estimated national average for full vaccination coverage was 62.0% [13]. Furthermore, our sample had lower coverage than the regional average for every vaccine, except BCG (90.1%) and MCV1 (79.7%) [2] (Table 2).

Among 161 under-vaccinated children who had a medical chart available at discharge, 98.1% (158/161) represented a missed opportunity for vaccination. Three children who expired during the study and 8 children who remained admitted in the hospital at study end (Figure 1) were excluded from the analysis. There was no significant difference of missed opportunities for vaccination by age. (Figure 2). Two children (aged 1 month and 17 months) were given their missed vaccinations in hospital, and a third child (aged 3 months) visited the immunization clinic for catch up vaccination prior to discharge. The discharge interview was completed among 88% (142/161) of caregivers of under-vaccinated children while the remaining 12% (19/161) were discharged outside of study hours. Among those interviewed, 62.9% of caregivers knew the

For peer review only

correct purpose of vaccination ('to prevent diseases'), 8.5% reported having their child's vaccination history verified by staff, 1.4% reported receiving vaccine recommendations and information on vaccine clinic locations, 1.4% were told about the potential vaccination side effects, and 90.1% reported they were comfortable having their children vaccinated while they were sick. When asked about vaccine reminder services or text message reminders for vaccinations after discharge, 95% of caregivers said they would support such measures, and 32.9% suggested there should be more vaccination outreach services.

Predictors of being under-vaccinated

In univariate analysis, there was a significant association between being under-vaccinated and the following determinants: children aged less than 4 months compared to older children (PR 1.26, 95% CI 1.04, 1.49), parents with lower than high school educational attainment (PR 1.45, 95% CI 1.15, 1.84), children of mothers not involved in vaccination decisions (PR 1.23, 95% CI 1.02, 1.50), those previously vaccinated in a private health facility (PR 1.32, 95% CI 1.04, 1.67), and children whose vaccination information was collected through parental recall due to the lack of an available vaccination card, (PR 1.48, 95% CI 1.25, 1.76). In multivariable analyses, there were similar trends between the outcome of being under-vaccinated and the predictors, although there was no statistical significance (Table 3).

Healthcare providers

Among 21 healthcare providers who participated across both sites (physicians and nurses from the pediatric inpatient units, emergency unit, and immunization clinic), 57% were female, the median age was 27 years and 76.2% reported having more than one year of experience working

For peer review only

in the healthcare field. Among the healthcare providers, 85.7% had incorrect knowledge of vaccination contraindications, listing low grade fever, pneumonia or diarrhea, and acute condition-related hospitalization as absolute contraindications. Although 95% agreed that it was appropriate to vaccinate a hospitalized child prior to discharge, 80% had concerns about vaccinating a child recovering from an acute illness, and 80% believed vaccines were not available in the inpatient unit. Reported barriers to vaccination of hospitalized children were, illness of the hospitalized child (71.4%), non-availability of immunization staff in the inpatient ward (71.4%), non-provision of vaccines in the inpatient ward (61.9%), and the focus of hospital health providers on management of acute illness rather than primary preventative measures (47.6%). Most (94.4%) believed these barriers could be addressed. Strategies suggested for improving catch-up vaccinations were: providing a reminder service at discharge for children missing vaccinations (85.7%), improving communication regarding vaccination status screening (76.2%), and improving vaccine delivery logistics and communication among hospital staff (66.7%).

Discussion

We observed a high proportion of under-vaccinated children among those hospitalized. This proportion was substantially higher than the national and regional averages for the period 2015-16 [2]. The overall proportion of missed opportunities for vaccination during hospitalization was 98.1%, which was significantly higher in comparison with other observational studies that included inpatient and outpatient healthcare facilities (25 - 43%) [9, 16-19]. A recent meta-analysis from seven low and middle income countries (LMICs) in Asia, Africa and the Americas found a pooled prevalence of missed vaccination opportunities among children accessing

For peer review only

healthcare facilities at 32% over a 22 year period [20]. An observational survey study in Mumbai found that healthcare providers missed opportunities to vaccinate children 80% of the time during a visit for an illness compared to only 0.7% of the time during a visit for a well child [21]. Using WHO standardized evaluation instruments, a study across 30 health facilities in Chad and Malawi found a higher proportion of missed vaccination opportunities in non-vaccination purpose visits (77%-92%), as seen in our study, compared to vaccination visits (47-51%) [22]. A descriptive analysis of private and public health facilities in four African countries found that 93-99% of eligible children remained under-vaccinated during outpatient non-vaccination visits based on the provider's response [23]. In line with these studies, our findings suggest that reducing missed vaccination opportunities can be impactful during inpatient non-vaccination hospital visits.

We observed that vaccination coverage among our sample of hospitalized children was substantially lower than nationally reported and regional vaccination coverage estimates for infant vaccination in general. Hospitalized children accessing healthcare in the public sector, such as our study sites, usually come from lower income quintiles from outside the cities seeking tertiary care and are often the most vulnerable to repeated events of ill health [24]. Individuals in the lowest-income quintiles are particularly vulnerable to illness as they are at higher risk of being malnourished, living in crowded conditions, and having less access to clean water [25]. In addition to conditions that contribute to poor health outcomes, the poorest children in India are less likely to be fully vaccinated, leaving them even more susceptible to preventable diseases, and leaving their caregivers susceptible to catastrophic health costs. We found that children who were in contact with a health facility in the last three months were less likely to be fully

For peer review only

vaccinated, suggesting a continual pattern of missed opportunities for vaccination. It is important in chracterizing this association to understanding that parents may not routinely seek vaccines or be aware that their children are under-vaccinated, hence it is crucial for facilities to devote more attention to vaccination screening among admitted children. Addressing missed opportunities for vaccination is even more important considering the COVID-19 pandemic, and its negative impact on routine vaccine-seeking behavior [26]. Interventions and communication strategies will be particularly important, as this will facilitate easy catch-up for vaccines missed during the pandemic, and it will also ensure there is infrastructure in place to rollout an eventual COVID-19 vaccine.

In this study, some healthcare provider perceptions could be significant barriers to provision of vaccinations. A study in Chad and Malawi found that 92% and 88% of healthcare providers, respectively, were unable to correctly identify valid contraindications for vaccination [22]. Our observations on a limited number of healthcare provider perceptions indicate that interventions targeting vaccination catch-up should focus on facility barriers training on vaccination schedules and contraindications, encourage screening for childhood vaccination status upon admission, and ensure that staff at inpatient and sick visit areas as well as in immunization clinics are well informed of the need for routine and catch-up childhood vaccination services. In studies that investigated the determinants of vaccination, barriers have been generalized into categories that included health system barriers (cost, infrastructure); provider barriers (poor knowledge of contraindications, poor access to immunization records); and caregiver barriers (economic problems, poor understanding of side effects) [27,28]. As barriers to vaccination are unique across regions, with varying determinants, understanding them at the caregiver and provider

For peer review only

level can lead to the design of context-specific interventions to drive vaccine uptake in the inpatient setting. A systematic review of barriers to vaccination and missed opportunities found risk factors associated with being under-vaccinated which were similar to our findings [20]. This review additionally identified lower household socioeconomic status, late initiation of the child's vaccination, parental information gap, provider practices, and a variety of clinic factors as contributing factors [28]. The first 3 months of life is an intense period for vaccinations, and is also a vulnerable time for illnesses. More research is needed to further explore the association among these risk factors, as well as test interventions that bring together community and facility-based care to ensure complete vaccination of all children. The MOV strategy designed by the WHO describes the standardized global practice for reducing missed opportunities for vaccination, which includes implementing the interventions, providing supportive supervision and moitoring progress, conducting rapid field evaluations of outcome, and incorporating into the long term vaccination plans to ensure sustainable goals [29]. Implementing screening and communication strategies to reduce missed opportunities for vaccination at the facility level will be crucial and cost-effective for enhancing and optimising vaccination coverage.

This study was limited by a small sample size and was completed within a short duration. We included those without a vaccination card and depended on parental recall in some children, which may have compromised reliability [30]. We took considerable precautions to ensure that hospital healthcare providers did not become greatly aware of the study details, and this minimized influencing their behavior during the study [31]. This study was only conducted at two government hospitals, which could limit the generalizability of the results. In India, patients that receive medical care at government-run hospitals have lower out-of-pocket expenses

For peer review only

compared to those accessing healthcare in the private sector, potentially biasing our sample based on facility selection [24]. While the two hospitals are in different states, there was no private hospital comparison in this preliminary study. However, we believe that focusing on government-run hospitals is a strength of our work, as it enabled us to characterize a population in India that is more prone to being under-vaccinated, while also allowing us to capture missed opportunities for vaccination among vulnerable populations [24, 28]. Moreover, the public sector is responsible for providing 60% of antenatal visits, as well as 90% of vaccination doses to children in India [24]. By conducting this research in the public sector, we were able to gain an understanding of missed opportunities, as well as barriers to vaccinations, in the setting where the vast majority of children are vaccinated. Interventions based on these insights have the potential to have a profound impact on vaccine uptake in India.

Conclusion

In conclusion, we found a high proportion of under-vaccination among hospitalized children, and the proportion of missed vaccination opportunities was high in these healthcare facilities. Our observation that a substantial proportion of under-vaccinated children have had prior contact with healthcare facilities underscores the need for interventions that repurpose existing resources at these healthcare facilities to ensure that vaccination opportunities are not missed. Addressing the system for vaccination screening at healthcare facilities, improved communication strategies targeted at both caregivers and healthcare providers and removing logistic barriers to vaccine access within the healthcare facility, can significantly improve vaccination inequities.

For peer review only

References

1 Peck M, Gacic-dobo M, Diallo MS, *et al.* Global Routine Vaccination Coverage, 2018. *MMWR* 2019;68:937–942. doi:http://dx.doi.org/10.15585/mmwr.mm6842a1

2 International Institute for Population Sciences (IIPS) and ICF. National Family Health Survey (NFHS-4) 2015-16 [Dataset]. Data Extract from IAIR72.SAV, IAKR72.SAV, IABR72.SAV, IAMR74.SAV, and IAPR74.SAV. IPUMS Demographic and Health Surveys (IPUMS DHS), 2018.

3 Ministries of Health and Family Welfare.. National Family Health Survey - 4 State Fact Sheet NCT Delhi. 2015; 1–36.

4 Ministries of Health and Family Welfare.. National Family Health Survey - 4 District Fact Sheet Jaipur Rajasthan. 2015.

5 Ministries of Health and Family Welfare. National Family Health Survey-4 State Fact Sheet Chandigarh. *Natl Fam Heal Surv – 4, Demogr Heal Surv* 2015.

6 Gurnani V, Haldar P, Aggarwal MK, *et al.* Improving vaccination coverage in India: lessons from Intensified Mission Indradhanush, a - cross-sectoral systems strengthening strategy. *bmj* 2018;363:1–7. doi:10.1136/bmj.k4782

For peer review only

7 Bhatnagar P, Gupta S, Kumar R, *et al.* Estimation of child vaccination coverage at state and national levels in India. *Bulletin of the World Health Organization* 2016;94:728–34. doi:10.2471/BLT.15.167593

8 Bvudzai P, Joao G, Shendale S, *et al.* Reducing missed opportunities for vaccination in selected provinces of Mozambique : A study protocol. *Gates Open Research* 2017;1:1–11. doi:10.12688/gatesopenres.12761.1

9 Kumar D, Aggarwal A, Gomber S. Immunization Status of Children Admitted to a Tertiary-care Hospital of North India : Reasons for Partial Immunization or Non-immunization. *Journal of health, population, and nutrition* 2010;28:300–4. doi:10.3329/jhpn.v28i3.5560

10 World Health Organization. Planning Guide to Reduce Missed Opportunities for Vaccination (MOV). 2016;:1–60.

11 Ministries of Health and Family Welfare. National Immunization Schedule (NIS) National Immunization Schedule in India; 2017. 2017. http://www.ihatepsm.com/blog/national-immunization-schedule-india-2017

12 World Health Organization. Methodology for the Assessment of Missed Opportunities for Vaccination. 2017;:1-72.

For peer review only

For peer review only - http://bmjopen.bmj.com/site/about/guidelines.xhtml

13 Ministries of Health and Family Welfare. National Family Health Survey - 4 India National Fact Sheet. *Natl Fam Heal Surv - 4* 2015.

14 Spiegelman D, Hertzmark E. Easy SAS Calculations for Risk or Prevalence Ratios and Differences. *Am J Epidemiol* 2005;162:199–200. doi:10.1093/aje/kwi188

15 World Health Organization. Training Course on Child Growth Assessment WHO Child Growth Standards. 2008;:7.

16 Garib Z, Vargas AL, Trumbo SP, *et al.* Missed Opportunities for Vaccination in the Dominican Republic : Results of an Operational Investigation. *BioMed research international* 2016. doi: https://doi.org/10.1155/2016/4721836

17 Tagbo B, Onwuasigwe C. Missed immunization opportunities among children in Enugu. *Niger J Paediatr* 2006;32:73-76

18 Deivanayagam N, Nedunchelian K, Mala N, *et al.* Missed opportunities for immunization in children under 2 years attending an urban teaching hospital. *Indian Pediatr* 1995;32:51–7.

19 Li AJ, Sudath T, Peiris R, *et al.* Opportunities to improve vaccination coverage in a country with a fledgling health system : Findings from an assessment of missed opportunities for vaccination among health center attendees — Timor. *Vaccine*

For peer review only

2019;37:4281–90. doi:10.1016/j.vaccine.2019.06.041

20 Sridhar S, Maleq N, Guillermet E, *et al.* A systematic literature review of missed opportunities for immunization in low- and middle-income countries. *Vaccine* 2014;32:6870–9. doi:10.1016/j.vaccine.2014.10.063

21 Muranjan Mehta, C., Pakhare, A. M. An Observational, Health Service Based Survey for Missed Opportunities for Immunization. *Indian Pediatr* 2011;48:633–6.

22 Ogbuanu IU, Li AJ, Anya BM, *et al.* Can vaccination coverage be improved by reducing missed opportunities for vaccination ? Findings from assessments in Chad and Malawi using the new WHO methodology. 2019;14:1–19. doi:10.1371/journal.pone.0210648

23 Olorunsaiye CZ, Langhamer MS, Wallace AS, *et al.* Missed opportunities and barriers for vaccination : a descriptive analysis of private and public health facilities in four African countries. *Pan Afr Med J* 2017;27:1–9. doi:10.11604/pamj.supp.2017.27.3.12083

24 Mahal A, Yazbeck AS, Peters DH, *et al.* The Poor and Health Service Use in India. 2001. doi: https://doi.org/10.11588/xarep.00003863

25 Agarwal S, Satyavada A, Kaushik S, *et al.* Urbanization, Urban Poverty and Health of the Urban Poor: Status, Challenges and the Way Forward. *Demography India* 2007;36:121–34.

For peer review only

26 Nelson R. COVID-19 disrupts vaccine delivery. *Lancet Infect Dis* 2020;20:546. doi:10.1016/S1473-3099(20)30304-2

27 Esposito S, Principi N, Cornaglia G, *et al.* Barriers to the vaccination of children and adolescents and possible solutions. *Clinical Microbiology and Infection* 2014;20:25-31. doi: https://doi.org/10.1111/1469-0691.12447

28 Santoli JM, Szilagyi PG, Rodewald LE. Barriers to immunization and missed opportunities. *Pediatr Ann* 1998;27:366–74. doi:10.3928/0090-4481-19980601-11

29 World Health Organization. Intervention Guidebook Intervention Guidebook for implementing and monitoring activities to reduce Missed Opportunities for Vaccination. 2019;:1-30

30 Miles M, Ryman TK, Dietz V, *et al.* Validity of vaccination cards and parental recall to estimate vaccination coverage: A systematic review of the literature. *Vaccine* 2013;31:1560–8. doi:10.1016/j.vaccine.2012.10.089

31 Leonard KL, Masatu MC. Using the Hawthorne effect to examine the gap between a doctor ' s best possible practice and actual performance. *J Dev Econ* 2010;93:226–34. doi:10.1016/j.jdeveco.2009.11.001

For peer review only

467 **Contributorship Statement:** NWA, JLM, and AS conceived the study with contributions from SS. NWA prepared the dataset and conducted the analysis of the data. NWA, BD, and AS prepared the first draft of the manuscript. NWA, RC, AT, and IB were responsible for overseeing the acquisition and management of the data for the study. All authors reviewed the drafts of this manuscript, provided critical input, and approved the final version for submission.

472

473 **Acknowledgements:** The authors thank Laura Lochlainn, Ikechukwu Udo, and Stephanie Shendale of the Department of Immunization, Vaccines & Biologicals, WHO headquarters (WHO-HQ) for early discussion and input on the study, and thank Farhad Khan for helpful discussions on the analysis and drafting of the manuscript. The authors also thank caregivers of hospitalized children who assisted in refining and piloting of the study questionnaires prior to study initiation.

479

480 **Funding:** The MOVAHC study was funded through the Bill and Melinda Gates Foundation Gates Grand Challenges (OPP1217304).

482

483 **Data sharing statement:** Additional data can be made available upon request by emaiing ashet1@jhu.edu.

For peer review only

Supplementary Data

Table 1. Characteristics of enrolled children, their caregivers, and the household (N = 263)

	n	(%)
**Child Age (Months)**		
Minimum	1	
Mean	13	
Median	7	
Maximum	58	
**Child Gender**		
Male	152	57.8%
Female	111	42.2%
**Child Order**		
First-born	119	45.2%
Second-born	87	33.1%
Third-born	36	13.7%
Fourth-born or younger	21	8.0%
**Caregiver Age (Years)**		
Minimum	18	
Mean	27	
Median	25	
Maximum	63	
**Caregiver Gender**[1]		
Male	56	21.4%
Female	206	78.6%
**Relationship to Child**[2]		
Mother	192	73.3%
Father	50	19.1%
Aunt / Uncle	11	4.2%
Grandparent	9	3.4%
**Can caregiver read and write**		
Yes	203	77.2%
No	60	22.8%
**Caregiver level of education**[3]		
No formal education / did not complete primary	58	22.1%
Primary / middle school	35	13.4%
High School	84	32.1%
College and postgraduate	85	32.4%
**Household Religion**		
Hindu	205	77.9%
Muslim	46	17.5%

Sikh	12	4.6%
**Primary Residence**		
Urban	86	32.7%
Rural	160	60.8%
Suburban	17	6.5%
**Household Total**		
Minimum	3	
Mean	8	
Median	7	
Maximum	26	
**Child been in contact with another health center before today**[4]		
Yes	138	53.7%
No	119	46.3%
**When was child admitted to the other health center**[5] (denominator includes those with prior health center contact - 138)		
Within last month	66	47.8%
1 – 6 months	45	32.6%
Over 6 months	27	19.6%
**Makes the primary decisions on vaccination in the household**[6]		
Mother	142	55.3%
Father	19	7.4%
Consensus of father and mother	72	28.0%
Grandparent or other relative	24	9.3%
**Distance of this hospital (PGIMER or SMS) from residence**[7]		
0-5 km	24	9.3%
6-10 km	20	7.8%
11-50 km	26	10.1%
>50 km	188	72.9%
**Distance of any health facility from residence**[8]		
0-5 km	177	68.9%
6-10 km	60	23.3%
11-50 km	18	7.0%
>50 km	2	0.8%
**Child has a vaccination card**		
Yes, present at hospital	154	58.6%
Yes, absent at hospital	89	33.8%
No	20	7.6%
**Source of Immunization History**		
Immunization card	157	59.7%
Parental recall	106	40.3%
**Immunization Status**		
Fully Vaccinated	91	34.6%
Partially Vaccinated	158	60.1%

Unvaccinated	14	5.3%

[1] 1 respondent missing

[2] 1 respondent missing

[3] 1 respondent missing

[4] 6 respondents missing

[5] Denominator is 138 children in contact with another health facilty before today

[6] 6 respondents missing

[7] 5 respondents missing

[8] 6 respondents missing

For peer review only

Table 2. Additional vaccine coverage (N = 263)

Vaccine Dose	Sample Immunization Status		
	Vaccinated	Age Eligible	Coverage
	N	N	(%)
f-IPV[1] Dose 1	108	263	41.1%
f-IPV[1] Dose 2	52	204	25.5%
Rotavirus Dose 1	74	263	28.1%
Rotavirus Dose 2	55	225	24.4%
Rotavirus Dose 3	35	204	17.2%
Measles 2 (Measles, MR[2], MMR[3])	18	44	40.9%
OPV[4] Booster	25	44	56.8%
DPT[5] Booster	26	44	59.1%

[1] Fractional dose of inactivated poliovirus vaccine (f-IPV).

[2] Measles -rubella (MR) vaccine.

[3] Measles-mumps-rubella (MMR) vaccine.

[4] Oral poliovirus vaccine (OPV).

[5] Diphtheria-tetanus-pertussis (DPT) vaccine.

For peer review only

Figure 1. Enrollment flow-chart for the study

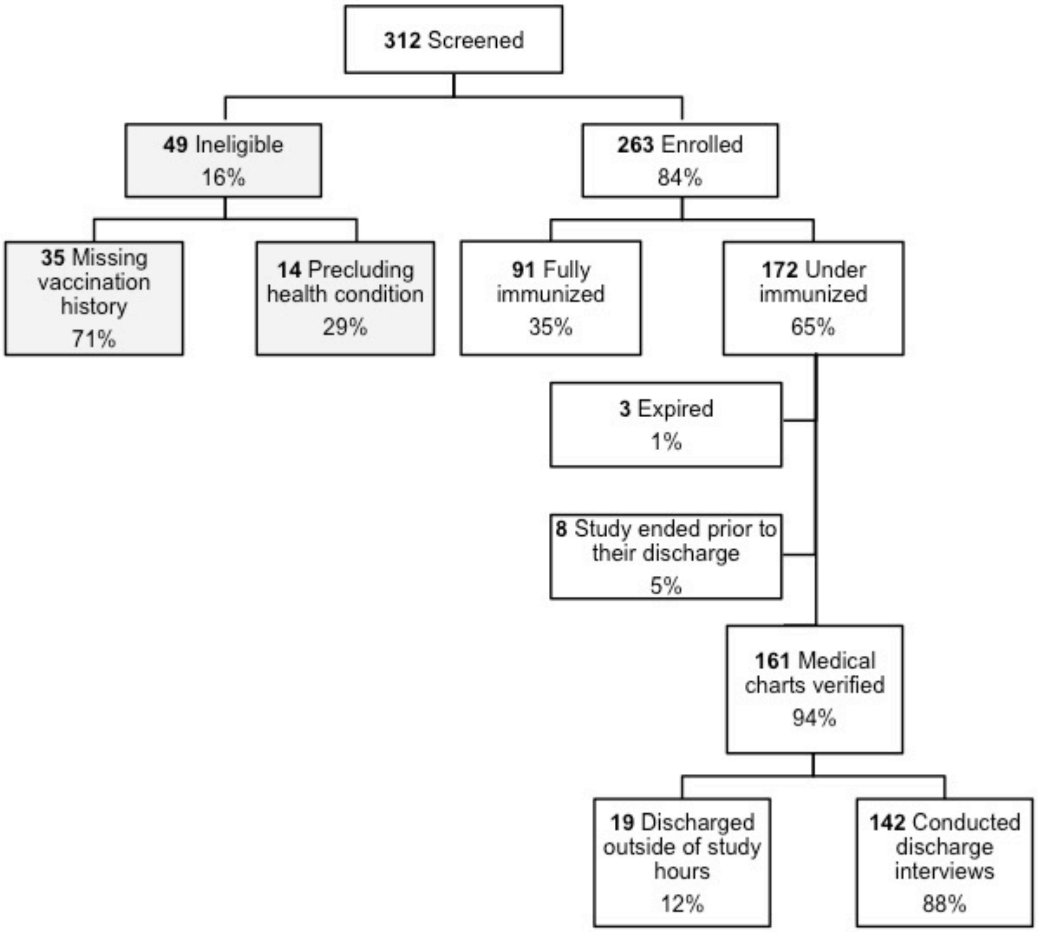

Fig. 1. Health-center–based flow-chart for determining eligibility. Missing vaccination history includes unable able to provide a vaccination card or accurately recall the vaccination history. Precluding health conditions include vaccination contraindications.

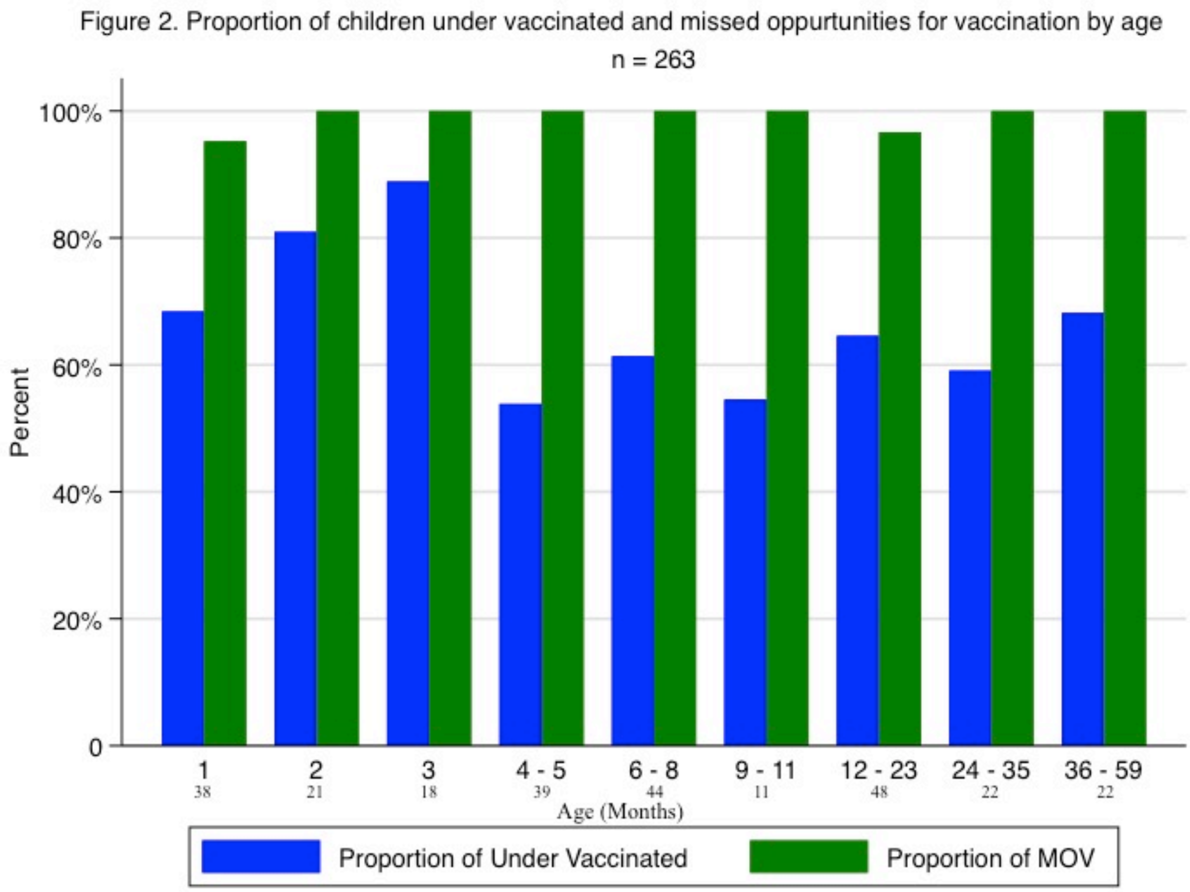

Fig. 2. The two main study outcomes are shown: proportion under vaccinated (in blue) and proportion of missed opportunities for vaccination (in green) by age range groupings (months). The number below the age range groupings on the x-axis show the count by age range grouping. The age range groupings correspond to when doses are given in the National Immunization Schedule.

For peer review only - http://bmjopen.bmj.com/site/about/guidelines.xhtml

Table 1. Study definitions for vaccination status by age range

Age range (months)	Birth Dose	First Dose	Second Dose	Third Dose	Booster Dose
<1 month	BCG				
1 month [4-7 weeks]	BCG<br>OPV	OPV OR f-IPV;<br><br>Penta OR DPT+Hep B			
2 months [8-11 weeks]	BCG<br>OPV	OPV OR f-IPV;<br><br>Penta OR DPT+Hep B	OPV OR f-IPV;<br><br>Penta OR DPT+Hep B		
3 – 8 months	BCG<br>OPV	OPV OR f-IPV;<br><br>Penta OR DPT+Hep B	OPV OR f-IPV;<br><br>Penta OR DPT+Hep B	OPV OR f-IPV;<br><br>Penta OR DPT+Hep B	
9 – 15 months	BCG<br>OPV	OPV OR f-IPV;<br><br>Penta OR DPT+Hep B;<br><br>Measles OR MMR OR MR	OPV OR f-IPV;<br><br>Penta OR DPT+Hep B	OPV OR f-IPV;<br><br>Penta OR DPT+Hep B	
16 – 59 months	BCG<br>OPV	OPV OR f-IPV;<br><br>Penta OR DPT+Hep B;<br><br>Measles OR MMR OR MR	OPV OR f-IPV;<br><br>Penta OR DPT+Hep B;<br><br>Measles OR MMR OR MR	OPV OR f-IPV;<br><br>Penta OR DPT+Hep B	OPV<br>DPT

Vaccination Status	Definition
**Fully vaccinated**	Child has had all of the recommended vaccines for his/her age group as defined above
**Partially vaccinated**	Child has had at least one, but not all, of the recommended vaccines for his/her age group as defined above
**Unvaccinated**	Child has had none of the recommended vaccines for his/her age group as defined above

BCG: Bacille Calmette-Guerin vaccine; OPV: Oral poliovirus vaccine; f-IPV: Fractional dose of inactivated poliovirus vaccine; Penta: Diphtheria-tetanus-pertussis-hepatitis B-Haemophilus influenzae type b pentavalent vaccine; DPT: Diphtheria-tetanus-pertussis vaccine; Hep B: Hepatitis B vaccine; Measles: Measles vaccine; MMR: Measles-mumps-rubella vaccine; MR: Measles-rubella vaccine

For peer review only

For peer review only - http://bmjopen.bmj.com/site/about/guidelines.xhtml

Table 2. Vaccine coverage compared to national vaccination coverage rates

Vaccine Dose	Vaccination Status among study population of hospitalized children (n=263)			Aggregated Chandigarh and Jaipur NFHS-4 Estimate [2015-2016][1]
	Vaccinated N	Age Eligible N	Coverage (%)	Coverage[2] (%)
BCG	237	263	90.1%	88.2%
OPV Dose 0	169	263	64.3%	71.7%
OPV Dose 1	182	263	69.2%	84.8%
OPV Dose 2	145	225	64.4%	78.2%
OPV Dose 3	110	204	53.9%	63.7%
DPT Dose 1 (DPT or Penta)	187	263	71.1%	83.6%
DPT Dose 2 (DPT or Penta)	142	225	63.1%	78.0%
DPT Dose 3 (DPT or Penta)	112	204	54.9%	69.6%
Measles 1 (Measles, MR, MMR)	55	69	79.7%	80.4%
Fully Vaccinated[3]	17	48	35.4%	62.0%

1 National Family Health Survey (NFHS-4) 2015-16 [Dataset] [1]
2 Coverage is from birth to 48 months of age. age range in our sample was from 1-59 months of age
3 NFHS defines fully vaccinated as having BCG, measles, and 3 doses each of polio and DPT by 12-23 months of age.

review only

Table 3. Prevalence rate ratio [PRR] of being under vaccinated based on multiple predictors (N = 263)

	Total		Under Vaccinated		Univariate Analysis			Multivariable Analysis[1]		
	N	(%)	N	(%)	PR	95% CI	p-value	aPR	95% CI	p-value
Child Gender										
Female	111	42%	71	64%	referent			referent		
Male	152	58%	101	66%	1.03	(0.87 , 1.24)	0.68	0.97	(0.80 , 1.18)	0.78
Child Age										
Over Four Months	186	71%	113	61%	referent			referent		
Under Four Months	77	29%	59	77%	1.26	(1.06 , 1.49)	<0.01*	1.04	(0.83 , 1.29)	0.74
Child Birth Order										
First	119	45%	71	60%	referent			referent		
After (2nd, 3rd, 4th)	144	55%	101	70%	1.18	(0.98 , 1.41)	0.08	1.15	(0.93 , 1.42)	0.19
Residency										
Urban/Suburban	103	39%	61	59%	referent			referent		
Rural	160	61%	111	69%	1.17	(0.97 , 1.42)	0.10	1.14	(0.91 , 1.43)	0.26
Caregiver Education (262)[2]										
College or beyond college	85	32%	44	26%	referent			referent		
High school	84	32%	57	33%	1.31	(1.02 , 1.69)	0.04	1.3	(0.97 , 1.75)	0.08
Less than high school	93	35%	70	41%	1.45	(1.15 , 1.84)	<0.01*	1.29	(0.99 , 1.68)	0.06
Health Facility Contact (257[2]										
Over three months	44	17%	25	15%	referent			referent		
Within last three months	94	37%	68	41%	1.27	(0.96 , 1.70)	0.10	1.13	(0.81 , 1.57)	0.49
Never	119	46%	74	44%	1.09	(0.82 , 1.47)	0.55	1.01	(0.73 , 1.40)	0.94
Health Facility Distance (257)[2]										
0 - 5 km	177	69%	111	63%	referent			referent		
5 - 50 km	80	31%	56	70%	1.12	(0.93 , 1.34)	0.24	0.99	(0.80 , 1.22)	0.92
Vaccination Decision Maker (257)[2]										
Mother involved	214	83%	133	62%	referent			referent		
Mother not involved	43	17%	33	77%	1.23	(1.02 , 1.50)	0.03*	1.16	(0.92 , 1.46)	0.22
Has Rotavirus Vaccine Dose 1										
Yes	74	28%	31	42%	referent			referent		
No	189	72%	141	75%	1.78	(1.34 , 2.36)	<0.01*	1.40	(1.01 , 1.95)	0.05
Prior Vaccination Setting (223)[2]										
Government	202	91%	124	61%	referent			referent		
Private	21	9%	17	81%	1.32	(1.04 , 1.67)	0.02*	1.35	(1.07 , 1.71)	0.02*
Have Their Vaccination Card										
Present at hospital	154	59%	84	55%	referent			referent		
Absent at hospital	109	41%	88	81%	1.48	(1.25 , 1.76)	<0.00	1.18	(0.94 , 1.47)	0.15

1 Adjusted for all variables in the table
2 Number in parenthesis refers to denomoninator in each category if different from the main denominator of 263.

BMJ Open

Determining the burden of missed opportunities for vaccination among children admitted in healthcare facilities in India: a cross-sectional study

Journal:	*BMJ Open*
Manuscript ID	bmjopen-2020-046464.R1
Article Type:	Original research
Date Submitted by the Author:	18-Feb-2021
Complete List of Authors:	Albaugh, Nicholas; Johns Hopkins University Bloomberg School of Public Health, Department of Epidemiology<br>Mathew, Joseph; Post Graduate Institute of Medical Education and Research, Pediatrics<br>Choudhary, Richa ; Sawai Man Singh Medical College and Hospital, Pediatrics<br>Sitaraman, Sadasivan; Sawai Man Singh Medical College and Hospital, Pediatrics<br>Tomar, Anjali ; Sawai Man Singh Medical College and Hospital, Pediatrics<br>Bajwa, Ishumeet ; Post Graduate Institute of Medical Education and Research, Pediatrics<br>Dhaliwal, Baldeep; Johns Hopkins University Bloomberg School of Public Health, International Health<br>Shet, Anita; Johns Hopkins University Bloomberg School of Public Health, International Health
**Primary Subject Heading**:	Paediatrics
Secondary Subject Heading:	Global health, Infectious diseases
Keywords:	PAEDIATRICS, Paediatric infectious disease & immunisation < PAEDIATRICS, PUBLIC HEALTH

SCHOLARONE™
Manuscripts

I, the Submitting Author has the right to grant and does grant on behalf of all authors of the Work (as defined in the below author licence), an exclusive licence and/or a non-exclusive licence for contributions from authors who are: i) UK Crown employees; ii) where BMJ has agreed a CC-BY licence shall apply, and/or iii) in accordance with the terms applicable for US Federal Government officers or employees acting as part of their official duties; on a worldwide, perpetual, irrevocable, royalty-free basis to BMJ Publishing Group Ltd ("BMJ") its licensees and where the relevant Journal is co-owned by BMJ to the co-owners of the Journal, to publish the Work in this journal and any other BMJ products and to exploit all rights, as set out in our licence.

The Submitting Author accepts and understands that any supply made under these terms is made by BMJ to the Submitting Author unless you are acting as an employee on behalf of your employer or a postgraduate student of an affiliated institution which is paying any applicable article publishing charge ("APC") for Open Access articles. Where the Submitting Author wishes to make the Work available on an Open Access basis (and intends to pay the relevant APC), the terms of reuse of such Open Access shall be governed by a Creative Commons licence – details of these licences and which Creative Commons licence will apply to this Work are set out in our licence referred to above.

Other than as permitted in any relevant BMJ Author's Self Archiving Policies, I confirm this Work has not been accepted for publication elsewhere, is not being considered for publication elsewhere and does not duplicate material already published. I confirm all authors consent to publication of this Work and authorise the granting of this licence.

For peer review only

Determining the burden of missed opportunities for vaccination among children admitted in healthcare facilities in India: a cross-sectional study

Nicholas W. Albaugh,[1,2] Joseph L. Mathew,[3] Richa Choudhary,[4] S. Sitaraman,[4] Anjali Tomar,[4] Ishumeet Kaur Bajwa,[3] Baldeep Dhaliwal,[2] Anita Shet[2,*]

1. Johns Hopkins Bloomberg School of Public Health, Department of Epidemiology, Baltimore, Maryland
2. Johns Hopkins Bloomberg School of Public Health, Department of International Health, Baltimore, Maryland
3. Postgraduate Institute of Medical Education and Research, Department of Pediatrics, Chandigarh, India
4. Sawai Man Singh Medical College, Department of Pediatrics, Jaipur, India

* Corresponding author at: Department of International Health, Johns Hopkins Bloomberg School of Public Health, 415 N. Washington Street, #535, Baltimore, MD 21205, USA.
Email: ashet1@jhu.edu

Word Count (excluding title page, abstract, references, figures and tables): 3,505
Tables: 3
Figures: 2
Supplementary Tables: 3

Keywords: vaccination, MOV, immunization status, India

Abbreviations:
aPR = Adjusted prevalence ratio
BCG = Bacille Calmette-Guerin vaccine
CI = Confidence interval
DPT = Diphtheria-tetanus-pertussis vaccine
f-IPV = Fractional dose of inactivated poliovirus vaccine
Hep B = Hepatitis B vaccine
MCV = Measles containing vaccine
MMR = Measles-mumps-rubella vaccine
MOV = Missed opportunity for vaccination
MR = Measles -rubella vaccine
OPV = Oral poliovirus vaccine
Penta = Diphtheria-tetanus-pertussis-hepatitis B-Haemophilus influenzae type b pentavalent (Penta) vaccine

For peer review only

ABSTRACT

Objectives

Children accessing healthcare systems represent a vulnerable population with risk factors for poor health outcomes, including vaccine-preventable diseases. We aimed to quantify missed vaccination opportunities among hospitalized children in India, and identify vaccination barriers perceived by caregivers and healthcare providers.

Design

Cross-sectional study

Setting

Two public-sector tertiary care hospitals in northern India, during November 2018 and March 2019.

Participants

We tracked 263 hospitalized children aged 1-59 months through hospital discharge, to assess vaccination status, and document catch-up vaccinations given during the hospital stay. We interviewed caregivers and healthcare providers to assess their perceptions on vaccination.

Outcomes

Proportion of hospitalized children considered under-vaccinated for their age; proportion of missed opportunities for vaccination among under-vaccinated children who were eligible for vaccination, and vaccine coverage by antigen.

For peer review only

Results

We found that 65.4% (172/263) of hospitalized children were under-vaccinated for their age when they presented to the hospital. Among under-vaccinated children, 61.0% were less than 4 months old, and 55.6% reported prior contact with a health facility for a sick visit. The proportion of under-vaccinated children in hospitals were higher compared to the general population as indicated by regional vaccination coverage data. Among under-vaccinated children who were tracked till discharge, 98.1% (158/161) remained incompletely vaccinated at discharge and were considered 'missed opportunities for vaccination'. Perceived vaccination contraindications that are not part of established contraindications included in national and international guidelines, was the most common reason for healthcare providers not to vaccinate children during hospital stay. Among caregivers of under-vaccinated children, 90.1% reported being comfortable having their children vaccinated while they were sick, if recommended by the healthcare provider.

Conclusion

This pilot study confirmed that hospitalized sick children had substantial missed vaccination opportunities. Addressing these opportunities through concerted actions involving caregivers, healthcare providers, and healthcare systems can improve overall vaccination coverage.

For peer review only

Strengths and limitations of this study

- This study focuses on children accessing healthcare facilities, and findings indicate that vaccination screening and catch-up vaccination at the inpatient health facility level provides an important opportunity to improve vaccination coverage and protection against vaccine-preventable diseases.
- The proportion of under-vaccinated children was higher among hospitalized children at the two regions in India compared to those in the general population as indicated by regional vaccination coverage information.
- A high proportion of hospitalized children were missed for catch-up vaccination during their admission and hospital course despite the available opportunities to provide catch-up vaccination.
- Over half of the under-vaccinated children reported prior healthcare facility contact for a sick visit, indicating that these children were well within reach of the heathcare system.

For peer review only

Introduction

Although global vaccination rates have remained high over for the last ten years[1], more than one out of every three children were not fully vaccinated in India in 2014[2]. According to the 2015-2016 National Family Health Survey (NFHS-4), vaccination rates have improved steadily with each required vaccine coverage increasing between 8-23 percentage points in overall vaccination coverage in India's National Immunization Schedule over the past ten years [3-5]. A massive nationwide effort called the Mission Indradhanush strategy was launched by the Government of India in 2014, which vaccinated approximately 25.5 million vulnerable children in a two-year span [6]. Nonetheless, a joint report in 2016 conducted by the World Health Organization (WHO), United Nations, and Government of India Ministry of Health and Family Welfare concluded that estimated vaccination rates tended to be overestimated by administrative vaccination coverage reports and must still be improved to meet the WHO Sustainable Development Goal (SDG # 3) of reducing child mortality [7]. The report acknowledged the challenges faced by the government in identifying and addressing 'missed vaccination' opportunities for children [7]. WHO defines a missed opportunity for vaccination (MOV) as any contact with health services by a child or adult who is eligible for vaccination, which does not result in the individual receiving all the vaccine doses for which he or she is eligible [8].

Hospitalized children represent a vulnerable population with risk factors for poor health outcomes, who are more likely than the general population to be eligible for catch-up vaccinations [9]. Children admitted to healthcare facilities may be at greater risk for infectious diseases, especially vaccine-preventable illnesses [10]. These children can benefit if vaccination opportunities are identified and addressed while the child is in the hospital. An analysis using

For peer review only

Demographic Health Surveys (DHS) and Multiple Indicator Cluster Surveys (MICS) estimated that if children in contact with health services for acute care were to receive their due vaccines, the potential increase in DTP3 vaccination coverage would be 3-14% in low and middle income countries (LMIC) settings, including a 12% increase in India [10]. Our study aimed to document vaccination status of hospitalized children by obtaining vaccination cards where available, verifying the medical records at hospital admission and discharge, and assessing vaccination barriers perceived by caregivers and healthcare providers. Our findings will be useful for estimating the proportion of missed opportunities for vaccination among hospitalized children in India, and to understand local barriers for vaccination that can potentially form the basis for contextually effective interventions.

Methods

This study was a cross-sectional observational research study at two tertiary care government hospitals in Chandigarh and Jaipur conducted between November 2018 and March 2019. Children aged 1-59 months, were screened for study eligibility at admission to the hospitals. Those children admitted to the hospital, and whose parents agreed to provide vaccination information and consented to the study, were enrolled. Children who had health conditions that precluded the use of vaccines, such as a known primary or secondary immune deficiency, malignancy requiring chemotherapy, and children with previous reported allergies or contraindications to any vaccine or vaccine component, were excluded from enrollment. Following written informed consent from the caregiver, concerted efforts were made to obtain the vaccination card from parents during the hospital stay. If the card was not present, the parent or caregiver was requested to have a family member bring the card from their home or send a

For peer review only

picture of the card using their mobile phone. Parental recall was used to document vaccination status only if the vaccination card was unavailable.

At admission, caregivers were interviewed about demographic details, household information and prior health seeking behaviors. Children who had received all recommended vaccines for their age, per the prevalent national immunization schedule, were classified as ‘fully vaccinated’ [11]. Those who had received some, but not all, of the recommended vaccines for their age group, were considered ‘partially vaccinated’. Those who had received none of the recommended vaccines for their age were labelled ‘unvaccinated’. Research team members used study definitions and the recommended national immunization schedule to classify children who were unvaccinated or partially vaccinated as ‘under-vaccinated’ (Table 1). This classification was verified during the analysis phase using the child’s age at time of interview and reported vaccinations. No discrepancies existed between research staff evaluation and analysis. Under-vaccinated children were tracked until discharge to record if any vaccinations, or advice regarding vaccinations, had been given during hospitalization or at discharge. The medical chart was reviewed to document, which vaccines, if any, were administered during hospitalization. The caregivers of under-vaccinated children were interviewed at discharge to assess their perspectives on vaccination within the context of the hospital.

Table 1. Study definitions for vaccination status by age range

Age range (months)	Birth Dose	First Dose	Second Dose	Third Dose	Booster Dose
<1 month	BCG				
1 month [4-7 weeks]	BCG OPV	OPV OR f-IPV;			

For peer review only

		Penta OR DPT+Hep B			
2 months [8-11 weeks]	BCG OPV	OPV OR f-IPV; Penta OR DPT+Hep B	OPV OR f-IPV; Penta OR DPT+Hep B		
3 – 8 months	BCG OPV	OPV OR f-IPV; Penta OR DPT+Hep B	OPV OR f-IPV; Penta OR DPT+Hep B	OPV OR f-IPV; Penta OR DPT+Hep B	
9 – 15 months	BCG OPV	OPV OR f-IPV; Penta OR DPT+Hep B; Measles OR MMR OR MR	OPV OR f-IPV; Penta OR DPT+Hep B	OPV OR f-IPV; Penta OR DPT+Hep B	
16 – 59 months	BCG OPV	OPV OR f-IPV; Penta OR DPT+Hep B; Measles OR MMR OR MR	OPV OR f-IPV; Penta OR DPT+Hep B; Measles OR MMR OR MR	OPV OR f-IPV; Penta OR DPT+Hep B	OPV DPT

**Vaccination Status**	**Definition**
**Fully vaccinated**	Child has had all of the recommended vaccines for his/her age group as defined above
**Partially vaccinated**	Child has had at least one, but not all, of the recommended vaccines for his/her age group as defined above
**Unvaccinated**	Child has had none of the recommended vaccines for his/her age group as defined above

BCG: Bacille Calmette-Guerin vaccine; OPV: Oral poliovirus vaccine; f-IPV: Fractional dose of inactivated poliovirus vaccine; Penta: Diphtheria-tetanus-pertussis-hepatitis B-Haemophilus influenzae type b pentavalent vaccine; DPT: Diphtheria-tetanus-pertussis vaccine; Hep B: Hepatitis B vaccine; Measles: Measles vaccine; MMR: Measles-mumps-rubella vaccine; MR: Measles-rubella vaccine

After the enrollment period, surveys were distributed to hospital healthcare providers involved in children's admission, treatment and discharge. Providers were asked to anonymously share their professional experiences, vaccination practices and related policies of the hospital. Eligible

For peer review only

healthcare providers included physicians, nurses, pharmacy personnel, and postgraduate residents in the inpatient unit and the emergency unit. Questions were derived from the WHO *Planning Guide to Reduce Missed Opportunities for Vaccination* and WHO *Methodology for the Assessment of Missed Opportunities for Vaccination* [10, 12].

Ethical considerations

This study was approved by The Institutional Review Board at the Johns Hopkins Bloomberg School of Public Health and local Institutional Ethics Committees at the Postgraduate Institute of Medical Education and Research (PGIMER), Chandigarh and Sawai Man Singh (SMS) Medical College and attached hospitals, Jaipur. Written informed consent was taken from participants prior to enrollment and implementation of study procedures. All anonymized data was collected on paper and entered into REDCap.

Patient and public involvement

Prior to study initiation and during the development process, we piloted the study questionnaires to refine them further. We involved some caregivers of hospitalized children for this piloting purpose, and their feedback was used to re-design the data collection methods. This contribution is noted in the acknowledgement section. Patients and their caregivers were active participants in this research, but were not involved in the conduct, analysis or dissemination of this research.

Statistical analysis

Study outcomes included (i) the proportion of hospitalized children considered under-vaccinated for their age; (ii) the proportion of missed opportunities for vaccination among under-vaccinated

For peer review only

children who were eligible for vaccination [8] and (iii) vaccine coverage by antigen. In addition, we compared these estimates of vaccine coverage among our sample of hospitalized children with regional Chandigarh and Jaipur estimates from the NFHS-4 published dataset [2]. For this sub-analysis to remain comparable with the NFHS-4 datasets, we only included children aged 12-23 months old using the NFHS-4 definition of 'fully vaccinated' who have received 1 dose of BCG, 3 doses each of polio and DPT+Hib+ Hep B (Penta) and 1 dose measles [13]. We fitted multivariable Poisson regression models for predictors of being under-vaccinated. Poisson regression is more appropriate than logistic regression due to the common (>10%) prevalence of being under-vaccinated and because the log binomial odds ratio models did not converge [14]. Weight-for-age z-scores were calculated using WHO growth indicator equations [15]. Caregiver and healthcare provider survey responses were coded and analyzed as binary outcomes for knowledge questions (correct/incorrect) and perception statements (agree/disagree). All statistical analyses were conducted using Stata (Version 13.1, StataCorp, College Station, Texas, United States).

Results

Of the 312 admitted children who were screened for enrollment, 49 were ineligible (35 had no vaccination card or parental recall of vaccination details and 14 had a prior precluding health condition), leaving 263 (62 at Chandigarh and 201 at Jaipur) who consented and were subsequently enrolled into the study (Figure 1).

Participant characteristics

For peer review only

The median age of children in our sample was 7 months (range, 1 to 58 months), and 57.8% (152/263) were male. First-born children made up 45.2% (119/263) of our sample. The median age of caregivers in the study was 25 years (ranging from 18 to 63 years), and 78.6% (206/262) were female, with the majority of caregivers being mothers. The highest attained education level was high school or greater among 64.5% of caregivers, and 77.2% could read and write. The primary residential setting was rural among 60.8% (160/263), with 31.1% (80/263) of children living more than 5 kilometers away from their nearest health center (Supplemental Table S1). At the time of this study, 92.4% (243/263) caregivers reported having a vaccination card for their children, however only 59.7% (157/263) were able to make this available at the hospital. Parental recall was used as the immunization source among the remaining 40.3% (106/263) (Supplemental Table S1).

Vaccination status

Among the 263 children included in the final analysis, 34.6% (91) of the children were fully vaccinated while 65.4% (172) were under-vaccinated. Among under-vaccinated children, 60.1% (158/263) and 5.3% (14/263) were partially vaccinated and unvaccinated, respectively. The proportion of under-vaccinated children was highest at 76.6%, (59/77) among those under four months of age (Figure 2). The average hospital duration among those under-vaccinated was 5 days (ranging from 1 to 58 days), and 47% (72/161) were classified as underweight (weight < 2 z-scores below the mean age-for weight WHO definition) [15]. Prior health facility contact for a sick visit at any time since birth, and within the last month, was reported in 55.6% (93/167) and 40.7% (68/167) of under-vaccinated children, respectively.

For peer review only

The vaccinations with the lowest coverage among all included children were OPV3 (53.9%), DPT3 (54.9%), and DPT2 (63.1%) (Table 2). Our sample also had less than 80% vaccination coverage for the following vaccines: OPV0 (64.3%), OPV1 (69.2%), and OPV2 (64.4%) and MCV1 (79.7%). The coverage of Measles-containing-vaccine second-dose (MCV2) [Measles, MR, MMR], OPV booster dose and DPT booster dose were 40.9%, 56.8% and 59.1% respectively (Supplemental Table S2). Using the NHFS-4 study definition of full vaccination, our sample of 12-23 months old children had a proportion of 35.4% (17/48) fully vaccinated children, while the reported 2015-2016 estimated national average for full vaccination coverage was 62.0% [13]. Furthermore, our sample had lower coverage than the regional average for every vaccine, except BCG (90.1%) and MCV1 (79.7%) [2] (Table 2). A similar look at the two states separately showed similar results (Supplemental Table S3).

Table 2. Vaccination coverage for individual vaccine antigens among hospitalized children at the study sites compared to national vaccination coverage rates from states (Chandigarh and Rajasthan NFHS-4)

	Vaccination coverage among study population of hospitalized children (n=263)			Aggregated Chandigarh and Rajasthan NFHS-4 estimates [2015-2016][1]
**Vaccine Dose**	**Vaccinated N**	**Age Eligible N**	**Coverage (%)**	**Coverage[2] (%)**
BCG	237	263	90.1%	88.2%
OPV Dose 0	169	263	64.3%	71.7%
OPV Dose 1	182	263	69.2%	84.8%
OPV Dose 2	145	225	64.4%	78.2%
OPV Dose 3	110	204	53.9%	63.7%
DPT Dose 1 (DPT or Penta)	187	263	71.1%	83.6%
DPT Dose 2 (DPT or Penta)	142	225	63.1%	78.0%
DPT Dose 3 (DPT or Penta)	112	204	54.9%	69.6%
Measles 1 (Measles, MR, MMR)	55	69	79.7%	80.4%

Fully Vaccinated[3]	17	48	35.4%	62.0%

1 International Institute for Population Sciences (IIPS) and ICF. National Family Health Survey (NFHS-4) 2015-16 [Dataset]. Data Extract from IAIR72.SAV, IAKR72.SAV, IABR72.SAV, IAMR74.SAV, and IAPR74.SAV. IPUMS Demographic and Health Surveys (IPUMS DHS), 2018. [2]
2 Coverage is from birth to 48 months of age. age range in our sample was from 1-59 months of age
3 NFHS defines fully vaccinated as having BCG, measles, and 3 doses each of polio and DPT by 12-23 months of age.

Among 161 under-vaccinated children who had a medical chart available at discharge, 98.1% (158/161) represented a missed opportunity for vaccination. The most common admission diagnoses were, lower respiratory tract infection, sepsis, fever for evaluation, and epilepsy. Three children who expired during the study and 8 children who remained admitted in the hospital at study end (Figure 1) were excluded from the analysis. There was no significant difference of missed opportunities for vaccination by age. (Figure 2). Two children (aged 1 month and 17 months) were given their missed vaccinations in hospital, and a third child (aged 3 months) visited the immunization clinic for catch up vaccination prior to discharge. The discharge interview was completed among 88% (142/161) of caregivers of under-vaccinated children while the remaining 12% (19/161) were discharged outside of study hours. Among those interviewed, 62.9% of caregivers knew the correct purpose of vaccination ('to prevent diseases'), 8.5% reported having their child's vaccination history verified by staff, 1.4% reported receiving vaccine recommendations and information on vaccine clinic locations, 1.4% were told about the potential vaccination side effects, and 90.1% reported they were comfortable having their children vaccinated while they were sick. When asked about vaccine reminder services or text message reminders for vaccinations after discharge, 95% of caregivers said they would support such measures, and 32.9% suggested there should be more vaccination outreach services.

Predictors of being under-vaccinated

For peer review only

In univariate analysis, there was a significant association between being under-vaccinated and the following determinants: children aged less than 4 months compared to older children (PR 1.26, 95% CI 1.04, 1.49), parents with lower than high school educational attainment (PR 1.45, 95% CI 1.15, 1.84), children of mothers not involved in vaccination decisions (PR 1.23, 95% CI 1.02, 1.50), those previously vaccinated in a private health facility (PR 1.32, 95% CI 1.04, 1.67), and children whose vaccination information was collected through parental recall due to the lack of an available vaccination card, (PR 1.48, 95% CI 1.25, 1.76). In multivariable analyses, there were similar trends between the outcome of being under-vaccinated and the predictors, although there was no statistical significance (Table 3).

For peer

Table 3. Prevalence rate ratio [PRR] of being under vaccinated based on multiple predictors (N = 263)

	Total		Under Vaccinated		Univariate Analysis			Multivariable Analysis[1]		
	N	(%)	N	(%)	PR	95% CI	p-value	aPR	95% CI	p-value
Child Gender										
Female	111	42%	71	64%	referent			referent		
Male	152	58%	101	66%	1.03	(0.87 , 1.24)	0.68	0.97	(0.80 , 1.18)	0.78
Child Age										
Over Four Months	186	71%	113	61%	referent			referent		
Under Four Months	77	29%	59	77%	1.26	(1.06 , 1.49)	<0.01*	1.04	(0.83 , 1.29)	0.74
Child Birth Order										
First	119	45%	71	60%	referent			referent		
After (2nd, 3rd, 4th)	144	55%	101	70%	1.18	(0.98 , 1.41)	0.08	1.15	(0.93 , 1.42)	0.19
Residency										
Urban/Suburban	103	39%	61	59%	referent			referent		
Rural	160	61%	111	69%	1.17	(0.97 , 1.42)	0.10	1.14	(0.91 , 1.43)	0.26
Caregiver Education (262)[2]										
College or beyond college	85	32%	44	26%	referent			referent		
High school	84	32%	57	33%	1.31	(1.02 , 1.69)	0.04	1.3	(0.97 , 1.75)	0.08
Less than high school	93	35%	70	41%	1.45	(1.15 , 1.84)	<0.01*	1.29	(0.99 , 1.68)	0.06
Health Facility Contact (257[2]										
Over three months	44	17%	25	15%	referent			referent		
Within last three months	94	37%	68	41%	1.27	(0.96 , 1.70)	0.10	1.13	(0.81 , 1.57)	0.49

For peer review only - http://bmjopen.bmj.com/site/about/guidelines.xhtml

Never	119	46%	74	44%	1.09	(0.82 , 1.47)	0.55	1.01	(0.73 , 1.40)	0.94
Health Facility Distance (257) [2]										
0 - 5 km	177	69%	111	63%	referent			referent		
5 - 50 km	80	31%	56	70%	1.12	(0.93 , 1.34)	0.24	0.99	(0.80 , 1.22)	0.92
Vaccination Decision Maker (257) [2]										
Mother involved	214	83%	133	62%	referent			referent		
Mother not involved	43	17%	33	77%	1.23	(1.02 , 1.50)	0.03*	1.16	(0.92 , 1.46)	0.22
Has Rotavirus Vaccine Dose 1										
Yes	74	28%	31	42%	referent			referent		
No	189	72%	141	75%	1.78	(1.34 , 2.36)	<0.01*	1.40	(1.01 , 1.95)	0.05
Prior Vaccination Setting (223) [2]										
Government	202	91%	124	61%	referent			referent		
Private	21	9%	17	81%	1.32	(1.04 , 1.67)	0.02*	1.35	(1.07 , 1.71)	0.02*
Have Their Vaccination Card										
Present at hospital	154	59%	84	55%	referent			referent		
Absent at hospital	109	41%	88	81%	1.48	(1.25 , 1.76)	<0.00	1.18	(0.94 , 1.47)	0.15

1 Adjusted for all variables in the table
2 Number in parenthesis refers to denominator in each category if different from the main denominator of 263.

Healthcare providers

Among 21 healthcare providers who participated across both sites (physicians and nurses from the pediatric inpatient units, emergency unit, and immunization clinic), 57% were female, the median age was 27 years and 76.2% reported having more than one year of experience working in the healthcare field. Among the healthcare providers, 85.7% had incorrect knowledge of vaccination contraindications, listing low grade fever, pneumonia or diarrhea, and acute condition-related hospitalization as absolute contraindications. Although 95% agreed that it was appropriate to vaccinate a hospitalized child prior to discharge, 80% had concerns about vaccinating a child recovering from an acute illness, and 80% believed vaccines were not available in the inpatient unit. Reported barriers to vaccination of hospitalized children were, illness of the hospitalized child (71.4%), non-availability of immunization staff in the inpatient ward (71.4%), non-provision of vaccines in the inpatient ward (61.9%), and the focus of hospital

For peer review only - http://bmjopen.bmj.com/site/about/guidelines.xhtml

249 health providers on management of acute illness rather than primary preventative measures
250 (47.6%). Most (94.4%) believed these barriers could be addressed. Strategies suggested for
251 improving catch-up vaccinations were: providing a reminder service at discharge for children
252 missing vaccinations (85.7%), improving communication regarding vaccination status screening
253 (76.2%), and improving vaccine delivery logistics and communication among hospital staff
254 (66.7%).

For peer review only

256 **Discussion**

257 We observed a high proportion of under-vaccinated children among those hospitalized. This
258 proportion was substantially higher than the national and regional averages for the period 2015-
259 16 [2]. The overall proportion of missed opportunities for vaccination during hospitalization was
260 98.1%, which was significantly higher in comparison with other observational studies that
261 included inpatient and outpatient healthcare facilities (25 - 43%) [9, 16-19]. A recent meta-
262 analysis from seven low and middle income countries (LMICs) in Asia, Africa and the Americas
263 found a pooled prevalence of missed vaccination opportunities among children accessing
264 healthcare facilities at 32% over a 22 year period [20]. An observational survey study in Mumbai
265 found that healthcare providers missed opportunities to vaccinate children 80% of the time
266 during a visit for an illness compared to only 0.7% of the time during a visit for a well child [21].
267 Using WHO standardized evaluation instruments, a study across 30 health facilities in Chad and
268 Malawi found a higher proportion of missed vaccination opportunities in non-vaccination
269 purpose visits (77%-92%), as seen in our study, compared to vaccination visits (47-51%) [22]. A
270 descriptive analysis of private and public health facilities in four African countries found that 93-
271 99% of eligible children remained under-vaccinated during outpatient non-vaccination, acute-

care visits based on the provider’s response [23]. In line with these studies, our findings suggest that reducing missed vaccination opportunities can be impactful during inpatient non-vaccination hospital visits.

We observed that vaccination coverage among our sample of hospitalized children was substantially lower than nationally reported and regional vaccination coverage estimates for infant vaccination in general. Hospitalized children accessing healthcare in the public sector, such as our study sites, usually come from lower income quintiles from outside the cities seeking tertiary care and are often the most vulnerable to repeated events of ill health [24]. Individuals in the lowest-income quintiles are particularly vulnerable to illness as they are at higher risk of being malnourished, living in crowded conditions, and having less access to clean water [25]. In addition to conditions that contribute to poor health outcomes, the poorest children in India are less likely to be fully vaccinated, leaving them even more susceptible to preventable diseases, and leaving their caregivers susceptible to catastrophic health costs. We found that children who were in contact with a health facility in the last three months were less likely to be fully vaccinated, suggesting a continual pattern of missed opportunities for vaccination. It is important in chracterizing this association to understanding that parents may not routinely seek vaccines or be aware that their children are under-vaccinated, hence it is crucial for facilities to devote more attention to vaccination screening among admitted children. Addressing missed opportunities for vaccination is even more important considering the COVID-19 pandemic, and its negative impact on routine vaccine-seeking behavior [26]. Interventions and communication strategies will be particularly important, as this will facilitate easy catch-up for vaccines missed during the

For peer review only

294 pandemic, and it will also ensure there is infrastructure in place to rollout an eventual COVID-19 vaccine.

In this study, some healthcare provider perceptions could be significant barriers to provision of vaccinations. A study in Chad and Malawi found that 92% and 88% of healthcare providers, respectively, were unable to correctly identify valid contraindications for vaccination [22]. Our observations on a limited number of healthcare provider perceptions indicate that interventions targeting vaccination catch-up should focus on facility barriers training on vaccination schedules and contraindications, encourage screening for childhood vaccination status upon admission, and ensure that staff at inpatient and sick visit areas as well as in immunization clinics are well informed of the need for routine and catch-up childhood vaccination services. In studies that investigated the determinants of vaccination, barriers have been generalized into categories that included health system barriers (cost, infrastructure); provider barriers (poor knowledge of contraindications, poor access to immunization records); and caregiver barriers (economic problems, poor understanding of side effects) [27,28]. As barriers to vaccination are unique across regions, with varying determinants, understanding them at the caregiver and provider level can lead to the design of context-specific interventions to drive vaccine uptake in the inpatient setting. A systematic review of barriers to vaccination and missed opportunities found risk factors associated with being under-vaccinated which were similar to our findings [20]. This review additionally identified lower household socioeconomic status, late initiation of the child's vaccination, parental information gap, provider practices, and a variety of clinic factors as contributing factors [28]. The first 3 months of life is an intense period for vaccinations, and is also a vulnerable time for illnesses. More research is needed to further explore the association

For peer review only

among these risk factors, as well as test interventions that bring together community and facility-based care to ensure complete vaccination of all children. The MOV strategy designed by the WHO describes the standardized global practice for reducing missed opportunities for vaccination, which includes implementing the interventions, providing supportive supervision and monitoring progress, conducting rapid field evaluations of outcome, and incorporating into the long term vaccination plans to ensure sustainable goals [29]. Implementing screening and communication strategies to reduce missed opportunities for vaccination at the facility level will be crucial and cost-effective for enhancing and optimising vaccination coverage.

This study was limited by a small sample size and was completed within a short duration. We included those without a vaccination card and depended on parental recall in some children, which may have affected reliability [30]. A systematic review on validity of parental recall for childhood vaccination surveys called into question the reliability of this method, but also concluded that excluding caregiver recall could result in considerable bias in vaccine coverage estimations [31]. We took considerable precautions to ensure that hospital healthcare providers did not become greatly aware of the study details, and this minimized influencing their behavior during the study [32]. Caregiver persepctives were assessed only among those whose children were under-vaccinated, due to limitations in follow-up. This study was only conducted at two government hospitals, which could limit the generalizability of the results. In India, patients that receive medical care at government-run hospitals have lower out-of-pocket expenses compared to those accessing healthcare in the private sector, potentially biasing our sample based on facility selection [24]. While the two hospitals are in different states, there was no private hospital comparison in this preliminary study. However, we believe that focusing on

For peer review only

government-run hospitals is a strength of our work, as it enabled us to characterize a population in India that is more prone to being under-vaccinated, while also allowing us to capture missed opportunities for vaccination among vulnerable populations [24, 28]. Moreover, the public sector is responsible for providing 60% of antenatal visits, as well as 90% of vaccination doses to children in India [24]. By conducting this research in the public sector, we were able to gain an understanding of missed opportunities, as well as barriers to vaccinations, in the setting where the vast majority of children are vaccinated. Interventions based on these insights have the potential to have a profound impact on vaccine uptake in India. This pilot study helped establish feasibility assessments and power calculations for an interventional trial for addressing missed opportunities for vaccination that has been initiated in India and Nigeria.

Conclusion

In conclusion, we found a high proportion of under-vaccination among hospitalized children, and the proportion of missed vaccination opportunities was high in these healthcare facilities. Our observation that a substantial proportion of under-vaccinated children have had prior contact with healthcare facilities underscores the need for interventions that repurpose existing resources at these healthcare facilities to ensure that vaccination opportunities are not missed. Addressing the system for vaccination screening at healthcare facilities, improved communication strategies targeted at both caregivers and healthcare providers and removing logistic barriers to vaccine access within the healthcare facility, can significantly improve vaccination inequities.

For peer review only

References

1 Peck M, Gacic-dobo M, Diallo MS, *et al.* Global Routine Vaccination Coverage, 2018. *MMWR* 2019;68:937–942. doi:http://dx.doi.org/10.15585/mmwr.mm6842a1

2 International Institute for Population Sciences (IIPS) and ICF. National Family Health Survey (NFHS-4) 2015-16 [Dataset]. Data Extract from IAIR72.SAV, IAKR72.SAV, IABR72.SAV, IAMR74.SAV, and IAPR74.SAV. IPUMS Demographic and Health Surveys (IPUMS DHS), 2018.

3 Ministries of Health and Family Welfare. National Family Health Survey - 4 State Fact Sheet NCT Delhi. 2015; 1–36. http://rchiips.org/NFHS/pdf/NFHS4/DL_FactSheet.pdf

4 Ministries of Health and Family Welfare. National Family Health Survey - 4 District Fact Sheet Jaipur Rajasthan. 2015. http://rchiips.org/nfhs/FCTS/RJ/RJ_FactSheet_110_Jaipur.pdf

5 Ministries of Health and Family Welfare. National Family Health Survey-4 State Fact Sheet Chandigarh. *Natl Fam Heal Surv – 4, Demogr Heal Surv* 2015. http://rchiips.org/nfhs/pdf/NFHS4/CH_FactSheet.pdf

6 Gurnani V, Haldar P, Aggarwal MK, *et al.* Improving vaccination coverage in India: lessons from Intensified Mission Indradhanush, a - cross-sectoral systems strengthening

For peer review only

strategy. *bmj* 2018;363:1–7. doi:10.1136/bmj.k4782

7 Bhatnagar P, Gupta S, Kumar R, *et al.* Estimation of child vaccination coverage at state and national levels in India. *Bulletin of the World Health Organization* 2016;94:728–34. doi:10.2471/BLT.15.167593

8 Bvudzai P, Joao G, Shendale S, *et al.* Reducing missed opportunities for vaccination in selected provinces of Mozambique : A study protocol. *Gates Open Research* 2017;1:1–11. doi:10.12688/gatesopenres.12761.1

9 Kumar D, Aggarwal A, Gomber S. Immunization Status of Children Admitted to a Tertiary-care Hospital of North India : Reasons for Partial Immunization or Non-immunization. *Journal of health, population, and nutrition* 2010;28:300–4. doi:10.3329/jhpn.v28i3.5560

10 World Health Organization. Planning Guide to Reduce Missed Opportunities for Vaccination (MOV). 2016;:1–60.

11 Ministries of Health and Family Welfare. National Immunization Schedule (NIS) National Immunization Schedule in India; 2017. 2017. http://www.ihatepsm.com/blog/national-immunization-schedule-india-2017

12 World Health Organization. Methodology for the Assessment of Missed Opportunities

For peer review only

for Vaccination. 2017;:1-72.

13 Ministries of Health and Family Welfare. National Family Health Survey - 4 India National Fact Sheet. *Natl Fam Heal Surv - 4* 2015.

14 Spiegelman D, Hertzmark E. Easy SAS Calculations for Risk or Prevalence Ratios and Differences. *Am J Epidemiol* 2005;162:199–200. doi:10.1093/aje/kwi188

15 World Health Organization. Training Course on Child Growth Assessment WHO Child Growth Standards. 2008;:7.

16 Garib Z, Vargas AL, Trumbo SP, *et al.* Missed Opportunities for Vaccination in the Dominican Republic : Results of an Operational Investigation. *BioMed research international* 2016. doi: https://doi.org/10.1155/2016/4721836

17 Tagbo B, Onwuasigwe C. Missed immunization opportunities among children in Enugu. *Niger J Paediatr* 2006;32:73-76

18 Deivanayagam N, Nedunchelian K, Mala N, *et al.* Missed opportunities for immunization in children under 2 years attending an urban teaching hospital. *Indian Pediatr* 1995;32:51–7.

19 Li AJ, Sudath T, Peiris R, *et al.* Opportunities to improve vaccination coverage in a

For peer review only

country with a fledgling health system : Findings from an assessment of missed opportunities for vaccination among health center attendees — Timor. *Vaccine* 2019;37:4281–90. doi:10.1016/j.vaccine.2019.06.041

20 Sridhar S, Maleq N, Guillermet E, *et al.* A systematic literature review of missed opportunities for immunization in low- and middle-income countries. *Vaccine* 2014;32:6870–9. doi:10.1016/j.vaccine.2014.10.063

21 Muranjan Mehta, C., Pakhare, A. M. An Observational, Health Service Based Survey for Missed Opportunities for Immunization. *Indian Pediatr* 2011;48:633–6.

22 Ogbuanu IU, Li AJ, Anya BM, *et al.* Can vaccination coverage be improved by reducing missed opportunities for vaccination ? Findings from assessments in Chad and Malawi using the new WHO methodology. 2019;14:1–19. doi:10.1371/journal.pone.0210648

23 Olorunsaiye CZ, Langhamer MS, Wallace AS, *et al.* Missed opportunities and barriers for vaccination : a descriptive analysis of private and public health facilities in four African countries. *Pan Afr Med J* 2017;27:1–9. doi:10.11604/pamj.supp.2017.27.3.12083

24 Mahal A, Yazbeck AS, Peters DH, *et al.* The Poor and Health Service Use in India. 2001. doi: https://doi.org/10.11588/xarep.00003863

25 Agarwal S, Satyavada A, Kaushik S, *et al.* Urbanization, Urban Poverty and Health of the

For peer review only

Urban Poor: Status, Challenges and the Way Forward. *Demography India* 2007;36:121–34.

26 Nelson R. COVID-19 disrupts vaccine delivery. *Lancet Infect Dis* 2020;20:546. doi:10.1016/S1473-3099(20)30304-2

27 Esposito S, Principi N, Cornaglia G, *et al.* Barriers to the vaccination of children and adolescents and possible solutions. *Clinical Microbiology and Infection* 2014;20:25-31. doi: https://doi.org/10.1111/1469-0691.12447

28 Santoli JM, Szilagyi PG, Rodewald LE. Barriers to immunization and missed opportunities. *Pediatr Ann* 1998;27:366–74. doi:10.3928/0090-4481-19980601-11

29 World Health Organization. Intervention Guidebook Intervention Guidebook for implementing and monitoring activities to reduce Missed Opportunities for Vaccination. 2019;:1-30

30 Miles M, Ryman TK, Dietz V, *et al.* Validity of vaccination cards and parental recall to estimate vaccination coverage: A systematic review of the literature. *Vaccine* 2013;31:1560–8. doi:10.1016/j.vaccine.2012.10.089

31 Modi RN, King C, Bar-Zeev N, *et al*. Caregiver recall in childhood vaccination surveys: Systematic review of recall quality and use in low- and middle-income settings. *Vaccine*

For peer review only

2018;36:4161-4170. doi: 10.1016/j.vaccine.2018.05.089

32 Leonard KL, Masatu MC. Using the Hawthorne effect to examine the gap between a doctor ' s best possible practice and actual performance. *J Dev Econ* 2010;93:226–34. doi:10.1016/j.jdeveco.2009.11.001

For peer review only

Contributorship Statement: NWA, JLM, and AS conceived the study with contributions from SS. NWA prepared the dataset and conducted the analysis of the data. NWA, BD, and AS prepared the first draft of the manuscript. NWA, RC, AT, and IB were responsible for overseeing the acquisition and management of the data for the study. All authors reviewed the drafts of this manuscript, provided critical input, and approved the final version for submission.

Acknowledgements: The authors thank Laura Lochlainn, Ikechukwu Udo, and Stephanie Shendale of the Department of Immunization, Vaccines & Biologicals, WHO headquarters (WHO-HQ) for early discussion and input on the study, and thank Farhad Khan for helpful discussions on the analysis and drafting of the manuscript. The authors are grateful to the caregivers of hospitalized children who assisted in refining and piloting of the study questionnaires prior to study initiation.

Funding: The MOVAHC study was funded through the Bill and Melinda Gates Foundation Gates Grand Challenges (OPP1217304).

For peer review only

Data sharing statement: Additional data can be made available upon request by emailing ashet1@jhu.edu

Figure Legends

Figure 1. Health-center–based flow-chart for determining eligibility. Missing vaccination history includes unable able to provide a vaccination card or accurately recall the vaccination history.

Figure 2. The two main study outcomes are shown: proportion under-vaccinated (in blue) and proportion who missed opportunities for vaccination while in the health facility (in green) by age range groupings (months). The number below the age range groupings on the x-axis show the number of children within each age range grouping. The age range groupings correspond to ages when doses are recommended by the National Immunization Schedule.

For peer review only

Figure 1. Enrollment flow-chart for the study

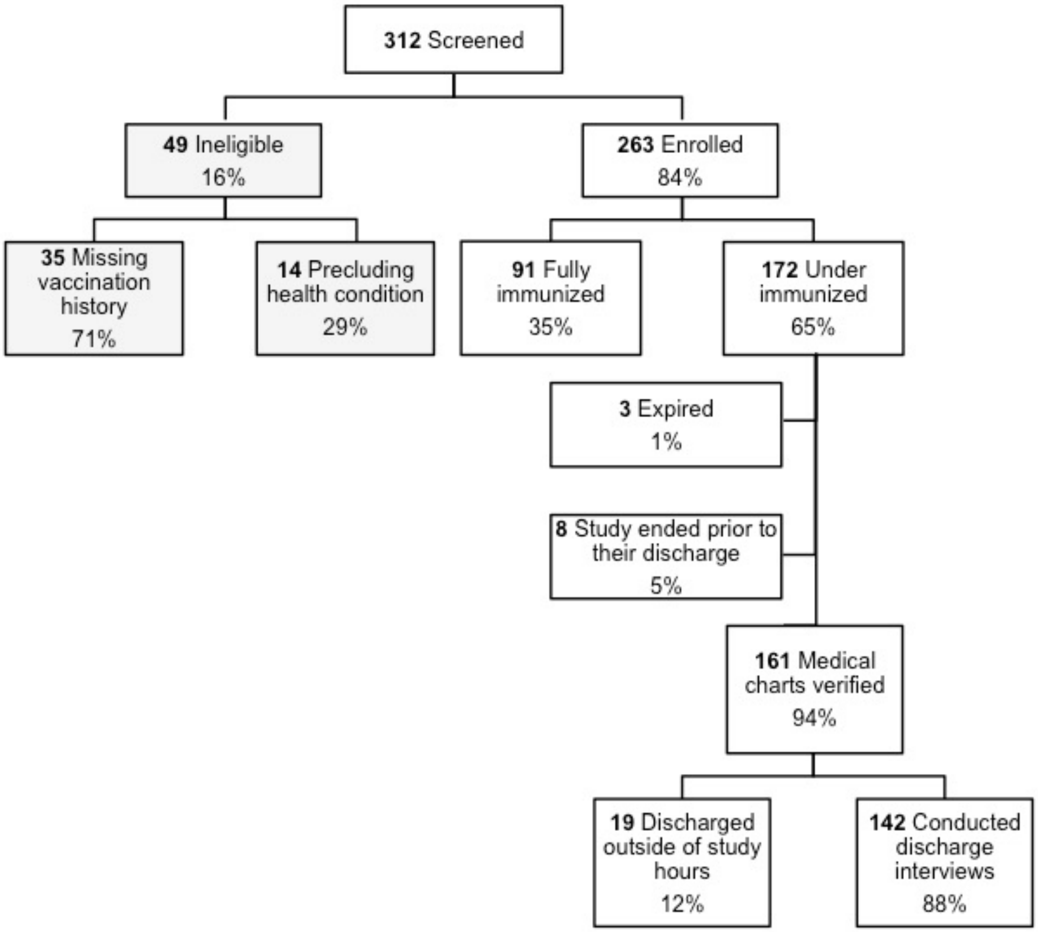

Figure 2. Proportion of children under vaccinated and missed oppurtunities for vaccination by age

n = 263

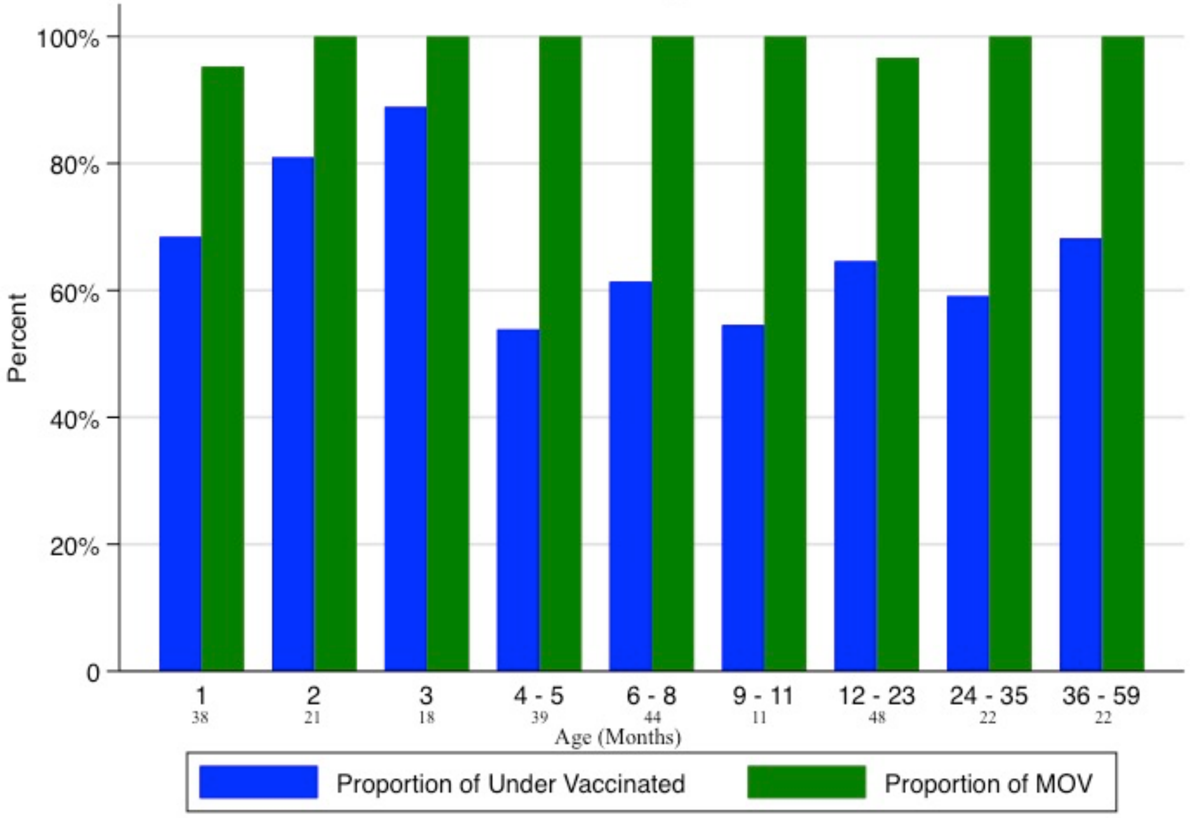

Table 1. Study definitions for vaccination status by age range

Age range (months)	Birth Dose	First Dose	Second Dose	Third Dose	Booster Dose
<1 month	BCG				
1 month<br>[4-7 weeks]	BCG<br>OPV	OPV OR<br>f-IPV;<br><br>Penta OR<br>DPT+Hep B			
2 months<br>[8-11 weeks]	BCG<br>OPV	OPV OR<br>f-IPV;<br><br>Penta OR<br>DPT+Hep B	OPV OR<br>f-IPV;<br><br>Penta OR<br>DPT+Hep B		
3 – 8 months	BCG<br>OPV	OPV OR<br>f-IPV;<br><br>Penta OR<br>DPT+Hep B	OPV OR<br>f-IPV;<br><br>Penta OR<br>DPT+Hep B	OPV OR<br>f-IPV;<br><br>Penta OR<br>DPT+Hep B	
9 – 15 months	BCG<br>OPV	OPV OR<br>f-IPV;<br><br>Penta OR<br>DPT+Hep B;<br><br>Measles OR<br>MMR OR<br>MR	OPV OR<br>f-IPV;<br><br>Penta OR<br>DPT+Hep B	OPV OR<br>f-IPV;<br><br>Penta OR<br>DPT+Hep B	
16 – 59 months	BCG<br>OPV	OPV OR<br>f-IPV;<br><br>Penta OR<br>DPT+Hep B;<br><br>Measles OR<br>MMR OR<br>MR	OPV OR<br>f-IPV;<br><br>Penta OR<br>DPT+Hep B;<br><br>Measles OR<br>MMR OR<br>MR	OPV OR<br>f-IPV;<br><br>Penta OR<br>DPT+Hep B	OPV<br>DPT

Vaccination Status	Definition
**Fully vaccinated**	Child has had all of the recommended vaccines for his/her age group as defined above
**Partially vaccinated**	Child has had at least one, but not all, of the recommended vaccines for his/her age group as defined above
**Unvaccinated**	Child has had none of the recommended vaccines for his/her age group as defined above

BCG: Bacille Calmette-Guerin vaccine; OPV: Oral poliovirus vaccine; f-IPV: Fractional dose of inactivated poliovirus vaccine; Penta: Diphtheria-tetanus-pertussis-hepatitis B-Haemophilus influenzae type b pentavalent vaccine; DPT: Diphtheria-tetanus-pertussis vaccine; Hep B: Hepatitis B vaccine; Measles: Measles vaccine; MMR: Measles-mumps-rubella vaccine; MR: Measles-rubella vaccine

For peer review only

Table 2. Vaccination coverage for individual vaccine antigens among hospitalized children at the study sites compared to national vaccination coverage rates from states (Rajasthan and Chandigarh, NFHS-4)

	Vaccination coverage among study population of hospitalized children (n=263)			Aggregated Chandigarh and Rajasthan NFHS-4 estimates [2015-2016][1]
Vaccine Dose	Vaccinated N	Age Eligible N	Coverage (%)	Coverage[2] (%)
BCG	237	263	90.1%	88.2%
OPV Dose 0	169	263	64.3%	71.7%
OPV Dose 1	182	263	69.2%	84.8%
OPV Dose 2	145	225	64.4%	78.2%
OPV Dose 3	110	204	53.9%	63.7%
DPT Dose 1 (DPT or Penta)	187	263	71.1%	83.6%
DPT Dose 2 (DPT or Penta)	142	225	63.1%	78.0%
DPT Dose 3 (DPT or Penta)	112	204	54.9%	69.6%
Measles 1 (Measles, MR, MMR)	55	69	79.7%	80.4%
Fully Vaccinated[3]	17	48	35.4%	62.0%

1 International Institute for Population Sciences (IIPS) and ICF. National Family Health Survey (NFHS-4) 2015-16 [Dataset]. Data Extract from IAIR72.SAV, IAKR72.SAV, IABR72.SAV, IAMR74.SAV, and IAPR74.SAV. IPUMS Demographic and Health Surveys (IPUMS DHS), 2018. [2]
2 Coverage is from birth to 48 months of age. age range in our sample was from 1-59 months of age
3 NFHS defines fully vaccinated as having BCG, measles, and 3 doses each of polio and DPT by 12-23 months of age.

review only

Table 3. Prevalence rate ratio [PRR] of being under vaccinated based on multiple predictors (N = 263)

	Total		Under Vaccinated		Univariate Analysis			Multivariable Analysis[1]		
	N	(%)	N	(%)	PR	95% CI	p-value	aPR	95% CI	p-value
Child Gender										
Female	111	42%	71	64%	referent			referent		
Male	152	58%	101	66%	1.03	(0.87 , 1.24)	0.68	0.97	(0.80 , 1.18)	0.78
Child Age										
Over Four Months	186	71%	113	61%	referent			referent		
Under Four Months	77	29%	59	77%	1.26	(1.06 , 1.49)	<0.01*	1.04	(0.83 , 1.29)	0.74
Child Birth Order										
First	119	45%	71	60%	referent			referent		
After (2nd, 3rd, 4th)	144	55%	101	70%	1.18	(0.98 , 1.41)	0.08	1.15	(0.93 , 1.42)	0.19
Residency										
Urban/Suburban	103	39%	61	59%	referent			referent		
Rural	160	61%	111	69%	1.17	(0.97 , 1.42)	0.10	1.14	(0.91 , 1.43)	0.26
Caregiver Education (262)[2]										
College or beyond college	85	32%	44	26%	referent			referent		
High school	84	32%	57	33%	1.31	(1.02 , 1.69)	0.04	1.3	(0.97 , 1.75)	0.08
Less than high school	93	35%	70	41%	1.45	(1.15 , 1.84)	<0.01*	1.29	(0.99 , 1.68)	0.06
Health Facility Contact (257[2]										
Over three months	44	17%	25	15%	referent			referent		
Within last three months	94	37%	68	41%	1.27	(0.96 , 1.70)	0.10	1.13	(0.81 , 1.57)	0.49
Never	119	46%	74	44%	1.09	(0.82 , 1.47)	0.55	1.01	(0.73 , 1.40)	0.94
Health Facility Distance (257)[2]										
0 - 5 km	177	69%	111	63%	referent			referent		
5 - 50 km	80	31%	56	70%	1.12	(0.93 , 1.34)	0.24	0.99	(0.80 , 1.22)	0.92
Vaccination Decision Maker (257)[2]										
Mother involved	214	83%	133	62%	referent			referent		
Mother not involved	43	17%	33	77%	1.23	(1.02 , 1.50)	0.03*	1.16	(0.92 , 1.46)	0.22
Has Rotavirus Vaccine Dose 1										
Yes	74	28%	31	42%	referent			referent		
No	189	72%	141	75%	1.78	(1.34 , 2.36)	<0.01*	1.40	(1.01 , 1.95)	0.05
Prior Vaccination Setting (223)[2]										
Government	202	91%	124	61%	referent			referent		
Private	21	9%	17	81%	1.32	(1.04 , 1.67)	0.02*	1.35	(1.07 , 1.71)	0.02*
Have Their Vaccination Card										
Present at hospital	154	59%	84	55%	referent			referent		
Absent at hospital	109	41%	88	81%	1.48	(1.25 , 1.76)	<0.00	1.18	(0.94 , 1.47)	0.15

1 Adjusted for all variables in the table
2 Number in parenthesis refers to denominator in each category if different from the main denominator of 263.

Supplementary Data

Table S1. Characteristics of enrolled children, their caregivers, and the household (N = 263)

	n	(%)
**Child Age (Months)**		
Minimum	1	
Mean	13	
Median	7	
Maximum	58	
**Child Gender**		
Male	152	57.8%
Female	111	42.2%
**Child Order**		
First-born	119	45.2%
Second-born	87	33.1%
Third-born	36	13.7%
Fourth-born or younger	21	8.0%
**Caregiver Age (Years)**		
Minimum	18	
Mean	27	
Median	25	
Maximum	63	
**Caregiver Gender[1]**		
Male	56	21.4%
Female	206	78.6%
**Relationship to Child[2]**		
Mother	192	73.3%
Father	50	19.1%
Aunt / Uncle	11	4.2%
Grandparent	9	3.4%
**Can caregiver read and write**		
Yes	203	77.2%
No	60	22.8%
**Caregiver level of education[3]**		
No formal education / did not complete primary	58	22.1%
Primary / middle school	35	13.4%
High School	84	32.1%
College and postgraduate	85	32.4%
**Household Religion**		
Hindu	205	77.9%
Muslim	46	17.5%

Sikh	12	4.6%
**Primary Residence**		
Urban	86	32.7%
Rural	160	60.8%
Suburban	17	6.5%
**Household Total**		
Minimum	3	
Mean	8	
Median	7	
Maximum	26	
**Child been in contact with another health center before today[4]**		
Yes	138	53.7%
No	119	46.3%
**When was child admitted to the other health center[5]** (denominator includes those with prior health center contact - 138)		
Within last month	66	47.8%
1 – 6 months	45	32.6%
Over 6 months	27	19.6%
**Makes the primary decisions on vaccination in the household[6]**		
Mother	142	55.3%
Father	19	7.4%
Consensus of father and mother	72	28.0%
Grandparent or other relative	24	9.3%
**Distance of this hospital (PGIMER or SMS) from residence[7]**		
0-5 km	24	9.3%
6-10 km	20	7.8%
11-50 km	26	10.1%
>50 km	188	72.9%
**Distance of any health facility from residence[8]**		
0-5 km	177	68.9%
6-10 km	60	23.3%
11-50 km	18	7.0%
>50 km	2	0.8%
**Child has a vaccination card**		
Yes, present at hospital	154	58.6%
Yes, absent at hospital	89	33.8%
No	20	7.6%
**Source of Immunization History**		
Immunization card	157	59.7%
Parental recall	106	40.3%
**Immunization Status**		
Fully Vaccinated	91	34.6%
Partially Vaccinated	158	60.1%

Unvaccinated	14	5.3%

[1] 1 respondent missing

[2] 1 respondent missing

[3] 1 respondent missing

[4] 6 respondents missing

[5] Denominator is 138 children in contact with another health facilty before today

[6] 6 respondents missing

[7] 5 respondents missing

[8] 6 respondents missing

For peer review only

Table S2. Additional vaccine coverage (N = 263)

Vaccine Dose	Vaccinated N	Age Eligible N	Coverage (%)
	Sample Immunization Status		
f-IPV[1] Dose 1	108	263	41.1%
f-IPV[1] Dose 2	52	204	25.5%
Rotavirus Dose 1	74	263	28.1%
Rotavirus Dose 2	55	225	24.4%
Rotavirus Dose 3	35	204	17.2%
Measles 2 (Measles, MR[2], MMR[3])	18	44	40.9%
OPV[4] Booster	25	44	56.8%
DPT[5] Booster	26	44	59.1%

[1] Fractional dose of inactivated poliovirus vaccine (f-IPV).

[2] Measles -rubella (MR) vaccine.

[3] Measles-mumps-rubella (MMR) vaccine.

[4] Oral poliovirus vaccine (OPV).

[5] Diphtheria-tetanus-pertussis (DPT) vaccine.

Table S3. Full vaccination coverage by site compared to state coverage rates (Rajasthan and Chandigarh, NFHS-4)

Fully vaccinated Jaipur study site		Fully vaccinated (Jaipur District, NFHS-4)		Fully vaccinated Chandigarh study site		Fully vaccinated (Chandigarh, NFHS-4)	
n	%	n[1]	%[1,2]	n	%	n[1]	%[1,2]
66	32.8%	5582	58.2	25	40.3	29	79.5

1 International Institute for Population Sciences (IIPS) and ICF. National Family Health Survey (NFHS-4) 2015-16 [Dataset]. Data Extract from IAIR72.SAV, IAKR72.SAV, IABR72.SAV, IAMR74.SAV, and IAPR74.SAV. IPUMS Demographic and Health Surveys (IPUMS DHS), 2018. [2]
2 Ministries of Health and Family Welfare. National Family Health Survey - 4 District Fact Sheet Jaipur Rajasthan. 2015. http://rchiips.org/nfhs/FCTS/RJ/RJ_FactSheet_110_Jaipur.pdf [4]
3 Ministries of Health and Family Welfare. National Family Health Survey-4 State Fact Sheet Chandigarh. *Natl Fam Heal Surv – 4, Demogr Heal Surv* 2015. http://rchiips.org/nfhs/pdf/NFHS4/CH_FactSheet.pdf [5]

Missed Opportunities for Vaccination Among Hospitalized Children (MOVAHC)

Caregiver Case Reporting Form 1

CRF 1, Part A: Risk Factor Survey

**The following section is information about the health facility and interviewer and will be filled out before administering the questionnaire**		
**No.**	**Questions**	**Coding categories**
0.1	Health facility name:	Serial number ☐☐
0.2	Questionnaire Serial Number	Serial number ☐☐☐☐
0.3	Name of interviewer:	______________________
0.4	Current Date	DD MM YYYY<br>☐☐ ☐☐ ☐☐☐☐
**Please make sure that consent was given before continuing. Please distribute one copy of the consent form to participants and save one copy of the consent form for research records.**		
1	Name of participant:	______________________
2	Address:<br>Please enter the entire address	______________________
2.1	Area (House No., Locality, Society):	______________________
2.2	Village/City Name	______________________
2.3	Local landmark:	______________________
2.4	District:	______________________
2.5	Pin code:	______________________
2.6	State:	______________________
3	Phone numbers:	Father:<br>Mother:<br>Other household member:<br>Other:
**Child Information: The following questions pertain to the admitted child of the caregiver.**		
4	What is your child's date of birth?	DD MM YYYY<br>☐☐ ☐☐ ☐☐☐☐
5	What is your child's age? (years/months)	☐☐ ☐☐
6	What is your child's gender?	1. Male<br>2. Female

For peer review only - http://bmjopen.bmj.com/site/about/guidelines.xhtml

		3. Other
7	What order was your child born?	1. First born<br>2. Second born<br>3. Third born<br>4. Forth born<br>5. Fifth born<br>6. Sixth or younger
**Caregiver Information: The following questions pertain to the caregiver of the admitted child.**		
8	What is your age? (Record age in completed years)	Years ☐☐
9	What is your gender?	1. Male<br>2. Female<br>3. Other
10	What is your relationship to the child?	1. Mother<br>2. Father<br>3. Grandparent<br>4. Uncle/aunt<br>5. Brother/sister<br>6. Non Household Member (neighbor/hired care giver etc.)<br>7. Other (please specify): ________________________
10.1	If "Other" was selected for the previous question then please specify:	________________________<br>________________________<br>________________________
11	Can you read?	1. Yes<br>2. No
12	Can you write?	1. Yes<br>2. No
13	What is your level of formal education?	1. No formal education<br>2. Did not complete primary (less than 4)<br>3. Primary school (up to 4th)<br>4. Middle school (5th-7th)<br>5. High school (8th-10th)<br>6. College (11th-12th)<br>7. Beyond college<br>8. Unknown
14	What do you do for a living?	1. Professional - tech high level<br>2. Professional - tech low level<br>3. Admin/exec/managers; clerical<br>4. Sales workers<br>5. Service workers (farm, fish, hunt, logging)<br>6. Production, transport

For peer review only - http://bmjopen.bmj.com/site/about/guidelines.xhtml

		7. Household duties<br>8. Students<br>9. Retired persons<br>10. Disabled persons<br>11. Unemployed persons<br>12. Other: ______________________
14.1	If "Other" was selected for the previous question then please specify:	______________________<br>______________________<br>______________________
**Household information: The following questions pertain to the household of the admitted child. Please answer to the best of your ability or skip if you do not know.**		
15	What religion does the head of the household follow?	1. Hindu<br>2. Muslim<br>3. Christian<br>4. Buddhist<br>5. Jain<br>6. Sikh<br>7. No Religion<br>8. Other, specify:<br>9. Unknown
16	What is your primary residence	1. Urban<br>2. Rural<br>3. Suburban<br>4. Other: ______________________
17	Are you a permanent residence of this city (where the hospital is)?	1. Yes<br>2. No
18	How many year(s) have you been in town where the hospital is?	1. Less than one<br>2. 1-2<br>3. 3-5<br>4. > 5 years
19	What is the number of living older siblings of the admitted child?	1. 0<br>2. 1<br>3. 2<br>4. 3 or more
20	What is the number of living younger siblings of the admitted child?	1. 0<br>2. 1<br>3. 2<br>4. 3 or more
21	How many (total) people usually live in the same household as this child (including	☐☐<br>People

IRB #9076

	the study child) (*household defined as sharing a cooking pot/area*)?	
**Hospital experience: The following questions pertain to your experience prior to the current hospital visit.**		
22	Has the child been in contact with a health center or admitted before today?	1. Yes<br>2. No
22.1	If "Yes" to the previous question, when was the child admitted to the health center	1. Within the last month<br>2. Within the last 3 months<br>3. Within the last 6 months<br>4. Within the last year<br>5. Over a year ago
23	Who makes the primary decisions on vaccination in the household?	1. Mother<br>2. Father<br>3. Consensus of father and mother<br>4. Grandparent<br>5. Other relative<br>6. Non-household member
24	How far is this health facility/ hospital from your house?	1. 0-5 km<br>2. 5-10 km<br>3. 10-50 km<br>4. >50km
25	How far is the nearest health facility/ hospital from your house?	1. 0-5 km<br>2. 5-10 km<br>3. 10-50 km<br>4. >50km
26	How did you hear about vaccinations? (CHECK ALL THAT APPLY)	1. Doctor<br>2. Nurse<br>3. Local health worker<br>4. Friend/neighbor<br>5. Family member<br>6. TV/radio<br>7. Other
26.1	If "Other" was selected for the previous question then please specify:	______________________<br>______________________<br>______________________
27	Does your child have a vaccination card?	1. Yes, and I have it with me<br>2. Yes, but I do not have it with me<br>3. No
27.1	If without a vaccination card/health passport, could you tell	1. It is at the nursery school/day care center<br>2. I left it at home (because I forgot to bring it)<br>3. I lost it

For peer review only - http://bmjopen.bmj.com/site/about/guidelines.xhtml

	us why you do not have the vaccination card/health passport with you today?	4. The card/health passport has been damaged<br>5. I have never been given one<br>6. Because vaccination was not the reason for this visit<br>7. Other: ________________________
27.1.1	If "Other" was selected for the previous question then please specify:	________________________<br>________________________<br>________________________
28	Has this child ever been vaccinated?	1. Yes<br>2. No
28.1	If "No" was selected for the previous question then why not?	1. I am not in favor of vaccination<br>2. My husband/the decision maker is not in favor of vaccination<br>3. I have not visited the health facility on a vaccination day<br>4. I did not know that the child was eligible to be vaccinated<br>5. Health center was too far<br>6. The necessary vaccines or supplies were not available<br>7. Heard that there are adverse reaction to vaccines/vaccines are not safe<br>8. Other: ________________________
28.1.1	If "Other" was selected for the previous question then please specify:	________________________<br>________________________<br>________________________
**Please proceed to the following form: Caregiver 1B – verifying the immunization card/status.**		

For peer review only

CRF 1, Part B: Verifying immunization status

The following section is information that will be filled out before verifying the immunization card		
**No.**	**Questions**	**Coding categories**
1	Health facility name:	Serial number ☐☐
2	Questionnaire Serial Number	Serial number ☐☐☐☐
3	Name of interviewer:	______________________________
4.1	What is the source of information?	1. From immunization card<br>2. From Immunization Register (ANM/Nurse/Doctor/MCTS register/Data base)<br>3. From hospital medical record<br>4. Based on caregiver recall only<br>5. Based on a combination of immunization card and caregiver recall
4.2	Was a photo of the immunization document taken?	1. Yes<br>2. No
**Please enter the following information about the immunization status of the child: If the vaccine(s) was given (yes/no), the date given (day/month/year), and any notified place of vaccination. If date is not given then leave date blank.**		

S. No	Vaccine	Status	Date
**5.1**	**BCG vaccine**	☐Yes ☐No	☐☐ ☐☐ ☐☐☐☐
**5.2**	**OPV (Oral Polio Vaccine)[1]**	☐Yes ☐No	
5.2.1	Dose 0	☐Yes ☐No	☐☐ ☐☐ ☐☐☐☐
5.2.2	Dose1	☐Yes ☐No	☐☐ ☐☐ ☐☐☐☐
5.2.3	Dose 2	☐Yes ☐No	☐☐ ☐☐ ☐☐☐☐
5.2.4	Dose 3	☐Yes ☐No	☐☐ ☐☐ ☐☐☐☐
5.2.5	Booster 1	☐Yes ☐No	☐☐ ☐☐ ☐☐☐☐
5.2.6	Last SIA dose ONLY	☐Yes ☐No	☐☐ ☐☐ ☐☐☐☐
**5.3**	**IPV (Injectable Polio Vaccine)**	☐Yes ☐No	
5.3.1	Dose1	☐Yes ☐No	☐☐ ☐☐ ☐☐☐☐
5.3.2	Dose 2	☐Yes ☐No	☐☐ ☐☐ ☐☐☐☐
5.3.3	Dose 3	☐Yes ☐No	☐☐ ☐☐ ☐☐☐☐

[1] Specify if monovalent, bivalent or trivalent OPV was given

For peer review only - http://bmjopen.bmj.com/site/about/guidelines.xhtml

S. No	Vaccine	Status	Date
**5.4**	**LPV (Liquid Pentavalent vaccine)[2,3]**	☐ Yes ☐ No	
5.4.1	Dose 1	☐ Yes ☐ No	
5.4.2	Dose 2	☐ Yes ☐ No	
5.4.3	Dose 3	☐ Yes ☐ No	
**5.5**	**Hepatitis B (Hepatitis B vaccine)[2]**	☐ Yes ☐ No	
5.5.1	Dose 0	☐ Yes ☐ No	
5.5.2	Dose 1	☐ Yes ☐ No	
5.5.3	Dose 2	☐ Yes ☐ No	
5.5.4	Dose 3	☐ Yes ☐ No	
**5.6**	**DPT (DwPT/ DaPT)-(Diphtheria, Pertussis and Tetanus)[2,3]**	☐ Yes ☐ No	
5.6.1	Dose1	☐ Yes ☐ No	
5.6.2	Dose 2	☐ Yes ☐ No	
5.6.3	Dose 3	☐ Yes ☐ No	
5.6.4	Booster 1	☐ Yes ☐ No	
**5.7**	**Hib (H Influenza b vaccine)[2]**	☐ Yes ☐ No	
5.7.1	Dose 1	☐ Yes ☐ No	
5.7.2	Dose 2	☐ Yes ☐ No	
5.7.3	Dose 3	☐ Yes ☐ No	
5.7.4	Booster 1	☐ Yes ☐ No	
**5.8**	**MMR (Measles, Mumps & Rubella vaccine)[4]**	☐ Yes ☐ No	
5.8.1	Dose 1	☐ Yes ☐ No	
5.8.2	Dose 2	☐ Yes ☐ No	
**S. No**	**Vaccine**	**Status**	**Date**
**5.9**	**MR (Measles & Rubella vaccine)[4]**	☐ Yes ☐ No	
5.9.1	Dose 1	☐ Yes ☐ No	
5.9.2	Dose 2	☐ Yes ☐ No	
5.9.3	Last SIA dose	☐ Yes ☐ No	
**5.10**	**Measles[4]**	☐ Yes ☐ No	

[2] Dates for doses 1, 2 and 3 of Hepatiti[illegible] DPT and [illegible]B vacci[illegible] case combination pentavalent vaccine was g[illegible]n

[4] If child has received combination dos[illegible] MR or M[illegible]R, please enter at appropriate place and should not be entered with measles(alone) rows

IRB #9076

For peer review only

5.10.1	Dose 1	☐ Yes ☐ No	☐☐ ☐☐ ☐☐☐☐
5.10.2	Dose 2	☐ Yes ☐ No	☐☐ ☐☐ ☐☐☐☐
5.10.3	Last SIA dose	☐ Yes ☐ No	
**5.11**	**PCV (Pneumococcal vaccine)**[5]	☐ Yes ☐ No	
5.11.1	Dose 1	☐ Yes ☐ No	☐☐ ☐☐ ☐☐☐☐
5.11.2	Dose 2	☐ Yes ☐ No	☐☐ ☐☐ ☐☐☐☐
5.11.3	Dose 3	☐ Yes ☐ No	☐☐ ☐☐ ☐☐☐☐
**5.12**	**Rotavirus vaccine**	☐ Yes ☐ No	
5.12.1	Dose 1	☐ Yes ☐ No	☐☐ ☐☐ ☐☐☐☐
5.12.2	Dose 2	☐ Yes ☐ No	☐☐ ☐☐ ☐☐☐☐
5.12.3	Dose 3	☐ Yes ☐ No	☐☐ ☐☐ ☐☐☐☐
**5.13**	**JE (Japanese Encephalitis vaccine)**	☐ Yes ☐ No	
5.13.1	Dose 1	☐ Yes ☐ No	☐☐ ☐☐ ☐☐☐☐
5.13.2	Dose 2	☐ Yes ☐ No	☐☐ ☐☐ ☐☐☐☐
5.13.3	Last SIA dose	☐ Yes ☐ No	☐☐ ☐☐ ☐☐☐☐
**5.14**	**Hepatitis A (Hepatitis A vaccine)**	☐ Yes ☐ No	
5.14.1	Dose 1	☐ Yes ☐ No	☐☐ ☐☐ ☐☐☐☐
5.14.2	Dose 2	☐ Yes ☐ No	☐☐ ☐☐ ☐☐☐☐
**5.15**	**Varicella/ Chickenpox vaccine**	☐ Yes ☐ No	
5.15.1	Dose 1	☐ Yes ☐ No	☐☐ ☐☐ ☐☐☐☐
**5.16**	**Typhoid**	☐ Yes ☐ No	☐☐ ☐☐ ☐☐☐☐
5.16.1	Dose 1	☐ Yes ☐ No	☐☐ ☐☐ ☐☐☐☐
**S. No**	**Vaccine**	**Status**	**Date**
5.16.2	Dose 2	☐ Yes ☐ No	☐☐ ☐☐ ☐☐☐☐
**5.17**	**Rabies Vaccine**	☐ Yes ☐ No	☐☐ ☐☐ ☐☐☐☐
5.17.1	Dose 1	☐ Yes ☐ No	☐☐ ☐☐ ☐☐☐☐
5.17.2	Dose 2	☐ Yes ☐ No	☐☐ ☐☐ ☐☐☐☐
5.17.3	Dose 3	☐ Yes ☐ No	☐☐ ☐☐ ☐☐☐☐

[5] Specify if PCV 10 or PCV13 was given

For peer review only - http://bmjopen.bmj.com/site/about/guidelines.xhtml

5.17.4	Dose 4	☐ Yes ☐ No	☐☐ ☐☐ ☐☐☐☐
5.17.5	Dose 5	☐ Yes ☐ No	☐☐ ☐☐ ☐☐☐☐
5.18	Influenza vaccine (Last done)	☐ Yes ☐ No	☐☐ ☐☐ ☐☐☐☐
**5.19**	**Any other vaccines (specify the last dose only in every case)**		
5.19.1		☐ Yes ☐ No	☐☐ ☐☐ ☐☐☐☐
5.19.2		☐ Yes ☐ No	☐☐ ☐☐ ☐☐☐☐
5.19.3		☐ Yes ☐ No	☐☐ ☐☐ ☐☐☐☐
**5.20**	**Vitamin A**	☐ Yes ☐ No	
5.20.1	Dose 1	☐ Yes ☐ No	☐☐ ☐☐ ☐☐☐☐
5.20.2	Dose 2	☐ Yes ☐ No	☐☐ ☐☐ ☐☐☐☐
**5.21**	**Place of vaccination** *(Even if one dose/one vaccine has been given at a particular type of health facility other than where rest were administered – the response to be marked as mixed)*	1. Government<br>2. Private<br>3. Mixed<br>4. Not given	

For peer review only - http://bmjopen.bmj.com/site/about/guidelines.xhtml

**Immunization Status: This part is to be filled out by the interviewers at the conclusion of the interview using the immunization source of information.**		
	Immunization Assessment:	1. Fully Immunized: The child has all of the required vaccinations for their age.<br>2. Partially Immunized: The child has some, but not all, of the required vaccinations for their age.<br>3. Unimmunized: The child has none of the required vaccinations for their age.
	Based on the immunization assessment, is the child eligible for part two of the study?	1. Yes<br>2. No
	Additional comments about the interview:	______________________________________ (7 lines)

For peer review only

For peer review only - http://bmjopen.bmj.com/site/about/guidelines.xhtml

Missed Opportunities for Vaccination Among Hospitalized Children (MOVAHC)

Caregiver Case Reporting Form 2

CRF 2, Part A: Vaccination Experience Survey

**The following section is information about the health facility and interviewer and will be filled out before administering the questionnaire**		
**No.**	**Questions**	**Coding categories**
0.1	Health facility name:	Serial number ☐☐
0.2	Questionnaire Serial Number	Serial number ☐☐☐☐
0.3	Name of interviewer:	______________________________
0.4	Current Date	DD ☐☐ MM ☐☐ YYYY ☐☐☐☐
**Please ask for the name of the participant. Make sure that the name matches the participant name on the Form 1A with the same serial number before continuing.**		
**Hospital Vaccination: The following questions pertain to the vaccination services that did (or did not) take place at the hospital**		
1	Are you satisfied with the service provided at the hospital	1. Yes<br>2. No
1.1	If satisfied ("Yes"), why were you satisfied with the service?<br><br>CHECK ALL THAT APPLY	1. Immediate attention<br>2. Friendly treatment by staff<br>3. No charge for service<br>4. The necessary vaccines and supplies were available<br>5. Other: ______________________
1.1.1	If "Other" was selected for the previous question then please specify:	______________________<br>______________________<br>______________________
1.2	If not satisfied (No), why were you NOT satisfied?<br><br>CHECK ALL THAT APPLY	1. Had to wait a long time<br>2. The staff was discourteous<br>3. The language that the health workers use is not clear<br>4. They did not explain what vaccines they had given the child<br>5. The necessary vaccines or supplies were not available<br>6. Other: ______________________

For peer review only - http://bmjopen.bmj.com/site/about/guidelines.xhtml

1.2.1	If "Other" was selected for the previous question then please specify:	_________________________<br>_________________________<br>_________________________
2	Could you tell me the purpose of vaccines?<br><br>(<u>DO NOT read out the options</u>)<br><br><u>CHECK ALL THAT APPLY</u>	1. To prevent diseases<br>2. So children will grow up healthy<br>3. To cure/heal diseases<br>4. They don't do any good<br>5. Not sure what they are for<br>6. Other: _________________________
2.1	If "Other" was selected for the previous question then please specify:	_________________________<br>_________________________<br>_________________________
3	During today's visit, did the personnel/staff ask you for the child's vaccination card/vaccination status?	1. Yes<br>2. No
3.1	Did you refuse vaccine services at any time during your stay?	1. Yes<br>2. No
4	Was your child vaccinated here during your stay?	1. Yes<br>2. No
4.1.1	If vaccination took place ("Yes"),<br><br>Were you given a prescription for catch up vaccinations?	1. Yes<br>2. No
4.1.2	If vaccination took place ("Yes"),<br><br>Did they tell you today what vaccines they gave the child?	1. Yes<br>2. No
4.1.3	If vaccination took place ("Yes"),<br><br>Did they tell you the date of the next	1. Yes<br>2. No

For peer review only - http://bmjopen.bmj.com/site/about/guidelines.xhtml

	vaccination appointment?	
4.1.4	If vaccination took place ("Yes"),<br><br>Did they write down for you the date of the next vaccination appointment?	1. Yes<br>2. No
4.1.5	If vaccination took place ("Yes"),<br><br>Did you receive information today on the reactions or side effects that can occur following vaccination?	1. Yes<br>2. No
4.1.6	If vaccination took place ("Yes"),<br><br>Did you receive instructions on what to do if your child has a fever or other discomfort post vaccination?	1. Yes<br>2. No
4.2.1	**If child was not vaccinated ask why they were not, and pick from the following options**<br>**(<u>DO NOT read out the options</u>)**<br><br>**<u>CHECK ALL THAT APPLY</u>**	
4.2.1.1	<u>REASONS RELATED TO THE HEALTH WORKERS</u>	1. The health worker who saw us did not tell me about vaccinating the child today<br>2. The doctor/nurse said that the child could not be vaccinated because s/he was sick<br>3. Other
4.2.1.2	<u>REASONS RELATED TO THE CAREGIVER</u>	4. The last time the child was vaccinated, he/she got sick or had a reaction.<br>5. My religion doesn't permit vaccination or I don't believe in vaccines<br>6. Vaccination was not the purpose of this visit<br>7. This child is already fully vaccinated for his/her age<br>8. I don't trust the health workers/the vaccines

For peer review only - http://bmjopen.bmj.com/site/about/guidelines.xhtml

		in this health facility<br>9. I didn't have time today to wait for vaccination<br>10. Other
4.2.1.3	REASONS RELATED TO THE HEALTH FACILITY (LOGISTICS & ORGANIZATION)	11. There were no vaccines in the health facility today<br>12. There were no syringes or other vaccination supplies<br>13. Today is not a vaccination day in this health facility<br>14. The vaccination area was closed<br>15. The person in charge of vaccinations was not there<br>16. There would have been a long wait<br>17. Other
4.2.2	If your child was eligible for vaccination but was not vaccinated today, did the health worker refer you to or inform you where you can receive the missing vaccine doses?	1. Yes<br>2. No
**Possible Interventions: The following questions will ask your opinion regarding possible interventions to reduce missed opportunities to vaccinate children in the hospital.**		
5	What suggestions do you have to improve vaccination services?<br><br>(DO NOT read out the options)<br><br>CHECK ALL THAT APPLY	1. There should be more vaccination personnel<br>2. There should be less of a wait<br>3. Hours and days when vaccination services are available should not be limited<br>4. Vaccination should remain free of charge<br>5. The treatment of the children being vaccinated and their families, should be friendlier<br>6. Vaccines should always be in stock<br>7. They should provide information on the vaccines that are being given, on the diseases that they prevent, and on the reactions that they produce.<br>8. More outreach services<br>9. Other: ________________________<br>10. None<br>11. Don't know
5.1	If "Other" was selected for the previous question then	________________________<br>________________________<br>________________________

For peer review only - http://bmjopen.bmj.com/site/about/guidelines.xhtml

	please specify:	
6	Are you comfortable having your child vaccinated at the hospital when you come for a sick visit?	1. Yes<br>2. No
7	Would a reminder service at discharge be helpful for getting your child their next vaccination?	1. Yes<br>2. No
8	Would a SMS reminder service at home be helpful for getting your child their next vaccination?	1. Yes<br>2. No
	Additional comments about the interview:	____________________<br>____________________<br>____________________<br>____________________<br>____________________<br>____________________<br>____________________
**Please proceed to the following form: Caregiver 2B – verifying the immunization card and medical chart.**		

For peer review only

For peer review only - http://bmjopen.bmj.com/site/about/guidelines.xhtml

CRF 2, Part B: Verifying immunization status

The following section is information that will be filled out before verifying the medical chart		
**No.**	**Questions**	**Coding categories**
1	Health facility name:	Serial number ☐☐
2	Questionnaire Serial Number	Serial number ☐☐☐☐
3	Name of interviewer:	______________________
4	Current Date	DD MM YYYY<br>☐☐ ☐☐ ☐☐☐☐
**The following information will come from verifying the medical chart or ask the treating physician**		
5	What was the admission time? (24 hour format)	Time HH:MM ☐☐ ☐☐
6	What was the discharge time? (24 hour format)	Time HH:MM ☐☐ ☐☐
7	What was the duration of the hospital stay?	Days ☐☐
8	What was the admission diagnosis?	______________________<br>______________________<br>______________________
9	What was the discharge diagnosis?	______________________<br>______________________<br>______________________
10	Was the child admitted to the Intensive care unit?	1. Yes<br>2. No
11	What is the child's weight?	kg ☐☐
12	What is the child's height?	cm ☐☐☐
13.1	What is the source of information?	1. From immunization card<br>2. From Immunization Register (ANM/Nurse/Doctor/MCTS register/Data base)<br>3. Medical Record<br>4. Based on caregiver recall only<br>5. Based on a combination of immunization card and caregiver recall

For peer review only - http://bmjopen.bmj.com/site/about/guidelines.xhtml

13.2	Was a photo of the immunization document taken?	1. Yes<br>2. No
13.3	Did the child receive any vaccinations during this visit?	1. Yes<br>2. No

Please enter the following information about the discharge immunization status of the child: If the vaccine(s) was given during hospitalization (yes/no), the date given (day/month/year), and any notified place of vaccination. If date is not given then leave date blank. Please only include vaccines that were given during this hospital admission/visit.

S. No	Vaccine	Status	Date
**14.1**	**BCG vaccine**	☐ Yes ☐ No	
**14.2**	**OPV (Oral Polio Vaccine)**[1]	☐ Yes ☐ No	
14.2.1	Dose 0	☐ Yes ☐ No	
14.2.2	Dose1	☐ Yes ☐ No	
14.2.3	Dose 2	☐ Yes ☐ No	
14.2.4	Dose 3	☐ Yes ☐ No	
14.2.5	Booster 1	☐ Yes ☐ No	
14.2.6	Last SIA dose ONLY	☐ Yes ☐ No	
**14.3**	**IPV (Injectable Polio Vaccine)**	☐ Yes ☐ No	
14.3.1	Dose1	☐ Yes ☐ No	
14.3.2	Dose 2	☐ Yes ☐ No	
14.3.3	Dose 3	☐ Yes ☐ No	
**S. No**	**Vaccine**	**Status**	**Date**
**14.4**	**LPV (Liquid Pentavalent vaccine)**[2,3]	☐ Yes ☐ No	
14.4.1	Dose 1	☐ Yes ☐ No	
14.4.2	Dose 2	☐ Yes ☐ No	
14.4.3	Dose 3	☐ Yes ☐ No	
**14.5**	**Hepatitis B (Hepatitis B vaccine)**[2]	☐ Yes ☐ No	
14.5.1	Dose 0	☐ Yes ☐ No	
14.5.2	Dose 1	☐ Yes ☐ No	

[1] Specify if monovalent, bivalent or triv[illegible] OPV w[illegible] given

For peer review only - http://bmjopen.bmj.com/site/about/guidelines.xhtml

14.5.3	Dose 2	☐ Yes ☐ No	
14.5.4	Dose 3	☐ Yes ☐ No	
**14.6**	**DPT (DwPT/ DaPT)- (Diphtheria, Pertussis and Tetanus)[2,3]**	☐ Yes ☐ No	
14.6.1	Dose1	☐ Yes ☐ No	
14.6.2	Dose 2	☐ Yes ☐ No	
14.6.3	Dose 3	☐ Yes ☐ No	
14.6.4	Booster 1	☐ Yes ☐ No	
**14.7**	**Hib (H Influenza b vaccine)[2]**	☐ Yes ☐ No	
14.7.1	Dose 1	☐ Yes ☐ No	
14.7.2	Dose 2	☐ Yes ☐ No	
14.7.3	Dose 3	☐ Yes ☐ No	
14.7.4	Booster 1	☐ Yes ☐ No	
**14.8**	**MMR (Measles, Mumps & Rubella vaccine)[4]**	☐ Yes ☐ No	
14.8.1	Dose 1	☐ Yes ☐ No	
14.8.2	Dose 2	☐ Yes ☐ No	
**S. No**	**Vaccine**	**Status**	**Date**
**14.9**	**MR (Measles & Rubella vaccine)[4]**	☐ Yes ☐ No	
14.9.1	Dose 1	☐ Yes ☐ No	
14.9.2	Dose 2	☐ Yes ☐ No	
14.9.3	Last SIA dose	☐ Yes ☐ No	
**14.10**	**Measles[4]**	☐ Yes ☐ No	
14.10.1	Dose 1	☐ Yes ☐ No	
14.10.2	Dose 2	☐ Yes ☐ No	
14.10.3	Last SIA dose	☐ Yes ☐ No	
**14.11**	**PCV (Pneumococcal vaccine)[5]**	☐ Yes ☐ No	
14.11.1	Dose 1	☐ Yes ☐ No	
14.11.2	Dose 2	☐ Yes ☐ No	
14.11.3	Dose 3	☐ Yes ☐ No	
**14.12**	**Rotavirus vaccine**	☐ Yes ☐ No	

[2] Dates for doses 1, 2 and 3 of Hepatitis [illegible] DPT and [illegible] B vacci[illegible] case combination pentavalent vaccine was g[illegible]

[4] If child has received combination dos[illegible] MR or M[illegible]R, pleas[illegible] should not be entered with measles(alone) rows

[5] Specify if PCV 10 or PCV13 was given

IRB #9076

For peer review only - http://bmjopen.bmj.com/site/about/guidelines.xhtml

14.12.1	Dose 1	☐ Yes ☐ No	☐☐ ☐☐ ☐☐☐☐
14.12.2	Dose 2	☐ Yes ☐ No	☐☐ ☐☐ ☐☐☐☐
14.12.3	Dose 3	☐ Yes ☐ No	☐☐ ☐☐ ☐☐☐☐
**14.13**	**JE (Japanese Encephalitis vaccine)**	☐ Yes ☐ No	
14.13.1	Dose 1	☐ Yes ☐ No	☐☐ ☐☐ ☐☐☐☐
14.13.2	Dose 2	☐ Yes ☐ No	☐☐ ☐☐ ☐☐☐☐
14.13.3	Last SIA dose	☐ Yes ☐ No	☐☐ ☐☐ ☐☐☐☐
**14.14**	**Hepatitis A (Hepatitis A vaccine)**	☐ Yes ☐ No	
14.14.1	Dose 1	☐ Yes ☐ No	☐☐ ☐☐ ☐☐☐☐
14.14.2	Dose 2	☐ Yes ☐ No	☐☐ ☐☐ ☐☐☐☐
**14.15**	**Varicella/ Chickenpox vaccine**	☐ Yes ☐ No	
14.15.1	Dose 1	☐ Yes ☐ No	☐☐ ☐☐ ☐☐☐☐
**14.16**	**Typhoid**	☐ Yes ☐ No	☐☐ ☐☐ ☐☐☐☐
14.16.1	Dose 1	☐ Yes ☐ No	☐☐ ☐☐ ☐☐☐☐
**S. No**	**Vaccine**	**Status**	**Date**
14.16.2	Dose 2	☐ Yes ☐ No	☐☐ ☐☐ ☐☐☐☐
**14.17**	**Rabies Vaccine**	☐ Yes ☐ No	☐☐ ☐☐ ☐☐☐☐
14.17.1	Dose 1	☐ Yes ☐ No	☐☐ ☐☐ ☐☐☐☐
14.17.2	Dose 2	☐ Yes ☐ No	☐☐ ☐☐ ☐☐☐☐
14.17.3	Dose 3	☐ Yes ☐ No	☐☐ ☐☐ ☐☐☐☐
14.17.4	Dose 4	☐ Yes ☐ No	☐☐ ☐☐ ☐☐☐☐
14.17.5	Dose 5	☐ Yes ☐ No	☐☐ ☐☐ ☐☐☐☐
14.18	Influenza vaccine (Last done)	☐ Yes ☐ No	☐☐ ☐☐ ☐☐☐☐
**14.19**	**Any other vaccines (specify the last dose only in every case)**		
14.19.1		☐ Yes ☐ No	☐☐ ☐☐ ☐☐☐☐
14.19.2		☐ Yes ☐ No	☐☐ ☐☐ ☐☐☐☐

For peer review only

14.19.3		☐ Yes ☐ No	☐☐ ☐☐ ☐☐☐☐
**14.20**	**Vitamin A**	☐ Yes ☐ No	
14.20.1	Dose 1	☐ Yes ☐ No	☐☐ ☐☐ ☐☐☐☐
14.20.2	Dose 2	☐ Yes ☐ No	☐☐ ☐☐ ☐☐☐☐

For peer review only

For peer review only - http://bmjopen.bmj.com/site/about/guidelines.xhtml

Missed Opportunities for Vaccination Among Hospitalized Children (MOVAHC)

Hospital Healthcare Worker Case Reporting Form 4

**By answering these questions, you are consenting to the study. Your personal identifiable information will not be collected or. Your answers will remain confidential and not shared with anyone outside of the research team.**	
Do you agree to participate in this study?	1. Agree<br>2. Disagree

**The following section is information about the health facility and will be filled out before administering the questionnaire**		
**No.**	**Questions**	**Coding categories**
0.1	Health facility name:	Serial number ☐☐
0.2	Classification of this health facility:	1. Public/Government<br>2. Private<br>3. Other: ________________
0.3	Type of health facility:	1. Hospital<br>2. Clinic<br>3. Health Center<br>4. Health Post
0.4	What is the pediatric inpatient bed capacity in this health facility?	Beds ☐☐☐

**Background: The following questions will ask about your work experience at the hospital.**		
**No.**	**Questions**	**Coding categories**
1	Participant serial number: (Will be provided for you)	☐☐☐
2	Current Date	DD MM YYYY<br>☐☐ ☐☐ ☐☐☐☐
3	Current Designation:	______________________________
4	What is your gender?	1. Male<br>2. Female<br>3. Other
5	What is your age? (Record age in completed	Years ☐☐

IRB #9076

For peer review only - http://bmjopen.bmj.com/site/about/guidelines.xhtml

	years)	
6	What is your professional training?	1. Doctor<br>2. Nurse<br>3. Hospital administrator<br>4. Pharmacy professional<br>5. Other: ________________
7	What is your primary area of work currently?	1. In-patient Department (in the admission wards)<br>2. General Out-Patient (OPD)<br>3. Emergency Department<br>4. Immunization clinic<br>5. Preventive medicine and epidemiology<br>6. Nutrition<br>7. IMCI (Integrated Management of Childhood Illnesses)<br>8. Ante-Natal Clinic (ANC)<br>9. Pharmacy<br>10. Other: ________________
8	For how long have you been working in this profession?	1. Less than 1 year<br>2. 1-3 years<br>3. 3-5 years<br>4. 5-10 years<br>5. More than 10 years
**Experience: The following questions will ask you about your personal experience when vaccinating children. Please answer to the best of your ability or select "I do not know".**		
9	What are the medical exceptions in which you should not vaccinate a child (i.e. contradictions)?<br><br><u>Please CHECK ALL CORRECT OPTIONS:</u>	1. Local reaction to previous dose<br>2. Light fever<br>3. Seizures well controlled under medical treatment<br>4. Pneumonia or diarrhea<br>5. Hospitalization for an acute condition<br>6. Serious injury<br>7. None of the above<br>8. I do not know
10	From day to day, which of the following individuals do you think should evaluate the vaccination status of children and ensure that children are up to date according to the national	1. The child's parents<br>2. The health worker responsible for immunization<br>3. Healthcare workers in hospital<br>4. Community leaders<br>5. All of the above<br>6. I do not know

For peer review only - http://bmjopen.bmj.com/site/about/guidelines.xhtml

	schedule?	
11	When do you think a hospitalized child requiring vaccinations should receive them?	1. Prior to discharge<br>2. After discharge at the same facility<br>3. After discharge at the facility selected by the caregiver<br>4. I do not know
12	Assuming there is no contradiction, do you think it is appropriate to vaccinate a hospitalized child prior to discharge?	1. Yes<br>2. No<br>3. I do not know
13	Please indicate if you agree or disagree with the following statement: "Assuming no absolute contraindications, I am still concerned about vaccinating a child recovering from an acute illness"	1. Agree<br>2. Disagree
13.1	Based on your previous answer, why do you feel that way?	________________________________<br>________________________________<br>________________________________
**Hospital Practice: The following questions will ask you about practices at your hospital regarding delivery of immunization services.**		
14	Is the immunization status of an admitted patient verified in the hospital?	1. Yes<br>2. No
15	Are vaccination services offered every day at this facility?	1. Yes, for all antigens<br>2. Yes, services are offered but not for all antigens<br>3. Yes, but:<br>4. No, certain days are scheduled as immunization days<br>5. No, but:
15.1	If selected "Yes, but" then please elaborate why?	________________________________<br>________________________________<br>________________________________
15.2	If selected "No, but" then please elaborate why not?	________________________________<br>________________________________<br>________________________________
16	Are vaccines available in the inpatient unit?	1. Yes<br>2. No
17	Are vaccines administered in	1. Yes

For peer review only - http://bmjopen.bmj.com/site/about/guidelines.xhtml

	the inpatient unit?	2. No
**Possible Interventions: The following questions will ask your opinion regarding possible interventions to reduce missed opportunities to vaccinate children in the hospital.**		
18	Do you think it is appropriate to vaccinate children in the inpatient unit?	1. Yes<br>2. No
19	What do you think are the barriers (if any) to providing vaccinations in the inpatient ward?<br><u>CHECK ALL THAT APPLY</u>	1. Vaccines are not usually provided in the inpatient ward<br>2. Immunization staff are not usually available in the inpatient ward<br>3. Children are usually too ill to be vaccinated in the inpatient ward<br>4. There is no time in the inpatient ward<br>5. Parents' are reluctant to vaccinate their hospitalized children<br>6. Hours of vaccination are incompatible with parents' busy lives<br>7. Physicians, nurses, and health workers are focused on the acute illness and do not review primary care aspects including vaccination schedules<br>8. Perceived contraindications for vaccination of a hospitalized child by health workers<br>9. Distance from vaccination clinic/lack of available vaccines at the inpatient or emergency unit<br>10. Other: ______________________<br>11. There are no barriers to providing vaccinations in the inpatient ward
20	Do you think some or all of these barriers can be overcome?	1. Yes<br>2. No
21	What strategies, if any, can this health facility employ to improve the number of children receiving all of their recommended vaccinations on time?	1. Improve communication regarding vaccination status screening<br>2. Improve communication regarding vaccine delivery<br>3. Provide a reminder service at discharge for children missing

For peer review only - http://bmjopen.bmj.com/site/about/guidelines.xhtml

	CHECK ALL THAT APPLY	vaccinations<br>4. Other:
21.1	If "Other", then please specify what strategies, if any, can this health facility employ to improve the number of children receiving all of their recommended vaccinations on time?	______________________________<br>______________________________<br>______________________________<br>______________________________<br>______________________________<br>______________________________<br>______________________________<br>______________________________<br>______________________________
22	Additional comments about the interview:	______________________________<br>______________________________<br>______________________________<br>______________________________<br>______________________________<br>______________________________<br>______________________________

For peer review only

STROBE Statement—Checklist of items that should be included in reports of ***cross-sectional studies***

	Item No	Page / Line no	Recommendation
**Title and abstract**	1	Page 1 (Title Page)	(*a*) Indicate the study's design with a commonly used term in the title or the abstract
		Page 2 – 3 (3-41)	(*b*) Provide in the abstract an informative and balanced summary of what was done and what was found
			**Introduction**
Background/rationale	2	Page 5 - 6 (58-83)	Explain the scientific background and rationale for the investigation being reported
Objectives	3	Page 6 (83-86)	State specific objectives, including any prespecified hypotheses
			**Methods**
Study design	4	Page 6 (92-96)	Present key elements of study design early in the paper
Setting	5	Page 6 (93)	Describe the setting, locations, and relevant dates, including periods of recruitment, exposure, follow-up, and data collection
Participants	6	Page 6 - 7 (94-104)	(*a*) Give the eligibility criteria, and the sources and methods of selection of participants
Variables	7	Page 8 – 9 (147-151)	Clearly define all outcomes, exposures, predictors, potential confounders, and effect modifiers. Give diagnostic criteria, if applicable
Data sources/ measurement	8*	Page 6 – 8 (100-129)	For each variable of interest, give sources of data and details of methods of assessment (measurement). Describe comparability of assessment methods if there is more than one group
Bias	9	Page 6 - 7(100-125)	Describe any efforts to address potential sources of bias
Study size	10	N/A	Explain how the study size was arrived at
Quantitative variables	11	Page 8 – 9 (147 – 160)	Explain how quantitative variables were handled in the analyses. If applicable, describe which groupings were chosen and why
Statistical methods	12	Page 8 – 9 (147 – 160)	(*a*) Describe all statistical methods, including those used to control for confounding
		N/A	(*b*) Describe any methods used to examine subgroups and interactions
		N/A	(*c*) Explain how missing data were addressed
		N/A	(*d*) If applicable, describe analytical methods taking account of sampling strategy
		N/A	(*e*) Describe any sensitivity analyses
			**Results**
Participants	13*	Page 9 (165-168)	(a) Report numbers of individuals at each stage of study—eg numbers potentially eligible, examined for eligibility, confirmed eligible, included in the study, completing follow-up, and analysed
		Page 9	(b) Give reasons for non-participation at each stage

For peer review only - http://bmjopen.bmj.com/site/about/guidelines.xhtml

		(165-168)	
		Page 9 (165-168)	(c) Consider use of a flow diagram
Descriptive data	14*	Page 10 (171-180)	(a) Give characteristics of study participants (eg demographic, clinical, social) and information on exposures and potential confounders
		N/A	(b) Indicate number of participants with missing data for each variable of interest
Outcome data	15*	Page 10 – 11 (184 – 206	Report numbers of outcome events or summary measures
Main results	16	Page 11 (224-232)	(*a*) Give unadjusted estimates and, if applicable, confounder-adjusted estimates and their precision (eg, 95% confidence interval). Make clear which confounders were adjusted for and why they were included
		Page 9 (171-173)	(*b*) Report category boundaries when continuous variables were categorized
		N/A	(*c*) If relevant, consider translating estimates of relative risk into absolute risk for a meaningful time period
Other analyses	17	Page 12 - 13 (235-251)	Report other analyses done—eg analyses of subgroups and interactions, and sensitivity analyses
			**Discussion**
Key results	18	Page 13 (254-258)	Summarise key results with reference to study objectives
Limitations	19	Page 16-17 (323-339)	Discuss limitations of the study, taking into account sources of potential bias or imprecision. Discuss both direction and magnitude of any potential bias
Interpretation	20	Page 14 – 15 (269-292)	Give a cautious overall interpretation of results considering objectives, limitations, multiplicity of analyses, results from similar studies, and other relevant evidence
Generalisability	21	Page 17 (341-354)	Discuss the generalisability (external validity) of the study results
			**Other information**
Funding	22	Page 24 (490-491)	Give the source of funding and the role of the funders for the present study and, if applicable, for the original study on which the present article is based

*Give information separately for exposed and unexposed groups.

Note: An Explanation and Elaboration article discusses each checklist item and gives methodological background and published examples of transparent reporting. The STROBE checklist is best used in conjunction with this article (freely available on the Web sites of PLoS Medicine at http://www.plosmedicine.org/, Annals of Internal Medicine at http://www.annals.org/, and Epidemiology at http://www.epidem.com/). Information on the STROBE Initiative is available at www.strobe-statement.org.

For peer review only

Supplementary Data

Table S1. Characteristics of enrolled children, their caregivers, and the household (N = 263)

	n	(%)
**Child Age (Months)**		
Minimum	1	
Mean	13	
Median	7	
Maximum	58	
**Child Gender**		
Male	152	57.8%
Female	111	42.2%
**Child Order**		
First-born	119	45.2%
Second-born	87	33.1%
Third-born	36	13.7%
Fourth-born or younger	21	8.0%
**Caregiver Age (Years)**		
Minimum	18	
Mean	27	
Median	25	
Maximum	63	
**Caregiver Gender[1]**		
Male	56	21.4%
Female	206	78.6%
**Relationship to Child[2]**		
Mother	192	73.3%
Father	50	19.1%
Aunt / Uncle	11	4.2%
Grandparent	9	3.4%
**Can caregiver read and write**		
Yes	203	77.2%
No	60	22.8%
**Caregiver level of education[3]**		
No formal education / did not complete primary	58	22.1%
Primary / middle school	35	13.4%
High School	84	32.1%
College and postgraduate	85	32.4%
**Household Religion**		
Hindu	205	77.9%
Muslim	46	17.5%

Sikh	12	4.6%
**Primary Residence**		
Urban	86	32.7%
Rural	160	60.8%
Suburban	17	6.5%
**Household Total**		
Minimum	3	
Mean	8	
Median	7	
Maximum	26	
**Child been in contact with another health center before today**[4]		
Yes	138	53.7%
No	119	46.3%
**When was child admitted to the other health center**[5] (denominator includes those with prior health center contact - 138)		
Within last month	66	47.8%
1 – 6 months	45	32.6%
Over 6 months	27	19.6%
**Makes the primary decisions on vaccination in the household**[6]		
Mother	142	55.3%
Father	19	7.4%
Consensus of father and mother	72	28.0%
Grandparent or other relative	24	9.3%
**Distance of this hospital (PGIMER or SMS) from residence**[7]		
0-5 km	24	9.3%
6-10 km	20	7.8%
11-50 km	26	10.1%
>50 km	188	72.9%
**Distance of any health facility from residence**[8]		
0-5 km	177	68.9%
6-10 km	60	23.3%
11-50 km	18	7.0%
>50 km	2	0.8%
**Child has a vaccination card**		
Yes, present at hospital	154	58.6%
Yes, absent at hospital	89	33.8%
No	20	7.6%
**Source of Immunization History**		
Immunization card	157	59.7%
Parental recall	106	40.3%
**Immunization Status**		
Fully Vaccinated	91	34.6%
Partially Vaccinated	158	60.1%

Unvaccinated	14	5.3%

[1] 1 respondent missing

[2] 1 respondent missing

[3] 1 respondent missing

[4] 6 respondents missing

[5] Denominator is 138 children in contact with another health facilty before today

[6] 6 respondents missing

[7] 5 respondents missing

[8] 6 respondents missing

For peer review only

Table S2. Additional vaccine coverage (N = 263)

Vaccine Dose	Sample Immunization Status: Vaccinated N	Age Eligible N	Coverage (%)
f-IPV[1] Dose 1	108	263	41.1%
f-IPV[1] Dose 2	52	204	25.5%
Rotavirus Dose 1	74	263	28.1%
Rotavirus Dose 2	55	225	24.4%
Rotavirus Dose 3	35	204	17.2%
Measles 2 (Measles, MR[2], MMR[3])	18	44	40.9%
OPV[4] Booster	25	44	56.8%
DPT[5] Booster	26	44	59.1%

[1] Fractional dose of inactivated poliovirus vaccine (f-IPV).

[2] Measles -rubella (MR) vaccine.

[3] Measles-mumps-rubella (MMR) vaccine.

[4] Oral poliovirus vaccine (OPV).

[5] Diphtheria-tetanus-pertussis (DPT) vaccine.

Table S3. Full vaccination coverage by site compared to state coverage rates (Rajasthan and Chandigarh, NFHS-4)

Fully vaccinated Jaipur study site		Fully vaccinated (Jaipur District, NFHS-4)		Fully vaccinated Chandigarh study site		Fully vaccinated (Chandigarh, NFHS-4)	
n	%	n[1]	%[1,2]	n	%	n[1]	%[1,2]
66	32.8%	5582	58.2	25	40.3	29	79.5

1 International Institute for Population Sciences (IIPS) and ICF. National Family Health Survey (NFHS-4) 2015-16 [Dataset]. Data Extract from IAIR72.SAV, IAKR72.SAV, IABR72.SAV, IAMR74.SAV, and IAPR74.SAV. IPUMS Demographic and Health Surveys (IPUMS DHS), 2018. [2]
2 Ministries of Health and Family Welfare. National Family Health Survey - 4 District Fact Sheet Jaipur Rajasthan. 2015. http://rchiips.org/nfhs/FCTS/RJ/RJ_FactSheet_110_Jaipur.pdf [4]
3 Ministries of Health and Family Welfare. National Family Health Survey-4 State Fact Sheet Chandigarh. *Natl Fam Heal Surv – 4, Demogr Heal Surv* 2015. http://rchiips.org/nfhs/pdf/NFHS4/CH_FactSheet.pdf [5]

BMJ Open

Determining the burden of missed opportunities for vaccination among children admitted in healthcare facilities in India: a cross-sectional study

Journal:	*BMJ Open*
Manuscript ID	bmjopen-2020-046464.R2
Article Type:	Original research
Date Submitted by the Author:	06-Mar-2021
Complete List of Authors:	Albaugh, Nicholas; Johns Hopkins University Bloomberg School of Public Health, Department of Epidemiology<br>Mathew, Joseph; Post Graduate Institute of Medical Education and Research, Pediatrics<br>Choudhary, Richa ; Sawai Man Singh Medical College and Hospital, Pediatrics<br>Sitaraman, Sadasivan; Sawai Man Singh Medical College and Hospital, Pediatrics<br>Tomar, Anjali ; Sawai Man Singh Medical College and Hospital, Pediatrics<br>Bajwa, Ishumeet ; Post Graduate Institute of Medical Education and Research, Pediatrics<br>Dhaliwal, Baldeep; Johns Hopkins University Bloomberg School of Public Health, International Health<br>Shet, Anita; Johns Hopkins University Bloomberg School of Public Health, International Health
**Primary Subject Heading**:	Paediatrics
Secondary Subject Heading:	Global health, Infectious diseases
Keywords:	PAEDIATRICS, Paediatric infectious disease & immunisation < PAEDIATRICS, PUBLIC HEALTH

SCHOLARONE™
Manuscripts

I, the Submitting Author has the right to grant and does grant on behalf of all authors of the Work (as defined in the below author licence), an exclusive licence and/or a non-exclusive licence for contributions from authors who are: i) UK Crown employees; ii) where BMJ has agreed a CC-BY licence shall apply, and/or iii) in accordance with the terms applicable for US Federal Government officers or employees acting as part of their official duties; on a worldwide, perpetual, irrevocable, royalty-free basis to BMJ Publishing Group Ltd ("BMJ") its licensees and where the relevant Journal is co-owned by BMJ to the co-owners of the Journal, to publish the Work in this journal and any other BMJ products and to exploit all rights, as set out in our licence.

The Submitting Author accepts and understands that any supply made under these terms is made by BMJ to the Submitting Author unless you are acting as an employee on behalf of your employer or a postgraduate student of an affiliated institution which is paying any applicable article publishing charge ("APC") for Open Access articles. Where the Submitting Author wishes to make the Work available on an Open Access basis (and intends to pay the relevant APC), the terms of reuse of such Open Access shall be governed by a Creative Commons licence – details of these licences and which Creative Commons licence will apply to this Work are set out in our licence referred to above.

Other than as permitted in any relevant BMJ Author's Self Archiving Policies, I confirm this Work has not been accepted for publication elsewhere, is not being considered for publication elsewhere and does not duplicate material already published. I confirm all authors consent to publication of this Work and authorise the granting of this licence.

For peer review only

Determining the burden of missed opportunities for vaccination among children admitted in healthcare facilities in India: a cross-sectional study

Nicholas W. Albaugh,[1,2] Joseph L. Mathew,[3] Richa Choudhary,[4] S. Sitaraman,[4] Anjali Tomar,[4] Ishumeet Kaur Bajwa,[3] Baldeep Dhaliwal,[2] Anita Shet[2,*]

1. Johns Hopkins Bloomberg School of Public Health, Department of Epidemiology, Baltimore, Maryland
2. Johns Hopkins Bloomberg School of Public Health, Department of International Health, Baltimore, Maryland
3. Postgraduate Institute of Medical Education and Research, Department of Pediatrics, Chandigarh, India
4. Sawai Man Singh Medical College, Department of Pediatrics, Jaipur, India

* Corresponding author at: Department of International Health, Johns Hopkins Bloomberg School of Public Health, 415 N. Washington Street, #535, Baltimore, MD 21205, USA. Email: ashet1@jhu.edu

Word Count (excluding title page, abstract, references, figures and tables): 3,667
Tables: 3
Figures: 2
Supplementary Tables: 3

Keywords: vaccination, MOV, immunization status, India

Abbreviations:
aPR = Adjusted prevalence ratio
BCG = Bacille Calmette-Guerin vaccine
CI = Confidence interval
DPT = Diphtheria-tetanus-pertussis vaccine
f-IPV = Fractional dose of inactivated poliovirus vaccine
Hep B = Hepatitis B vaccine
MCV = Measles containing vaccine
MMR = Measles-mumps-rubella vaccine
MOV = Missed opportunity for vaccination
MR = Measles -rubella vaccine
OPV = Oral poliovirus vaccine
Penta = Diphtheria-tetanus-pertussis-hepatitis B-Haemophilus influenzae type b pentavalent (Penta) vaccine

For peer review only

ABSTRACT

Objectives

Children accessing healthcare systems represent a vulnerable population with risk factors for poor health outcomes, including vaccine-preventable diseases. We aimed to quantify missed vaccination opportunities among hospitalized children in India, and identify vaccination barriers perceived by caregivers and healthcare providers.

Design

Cross-sectional study

Setting

Two public-sector tertiary care hospitals in northern India, during November 2018 and March 2019.

Participants

We tracked 263 hospitalized children aged 1-59 months through hospital discharge, to assess vaccination status, and document catch-up vaccinations given during the hospital stay. We interviewed caregivers and healthcare providers to assess their perceptions on vaccination.

Outcomes

Proportion of hospitalized children considered under-vaccinated for their age; proportion of missed opportunities for vaccination among under-vaccinated children who were eligible for vaccination, and vaccine coverage by antigen.

For peer review only

Results

We found that 65.4% (172/263) of hospitalized children were under-vaccinated for their age when they presented to the hospital. Among under-vaccinated children, 61.0% were less than 4 months old, and 55.6% reported prior contact with a health facility for a sick visit. The proportion of under-vaccinated children in hospitals were higher compared to the general population as indicated by regional vaccination coverage data. Among under-vaccinated children who were tracked till discharge, 98.1% (158/161) remained incompletely vaccinated at discharge and were considered 'missed opportunities for vaccination'. Perceived vaccination contraindications that are not part of established contraindications included in national and international guidelines, was the most common reason for healthcare providers not to vaccinate children during hospital stay. Among caregivers of under-vaccinated children, 90.1% reported being comfortable having their children vaccinated while they were sick, if recommended by the healthcare provider.

Conclusion

This pilot study confirmed that hospitalized sick children had substantial missed vaccination opportunities. Addressing these opportunities through concerted actions involving caregivers, healthcare providers, and healthcare systems can improve overall vaccination coverage.

For peer review only

Strengths and limitations of this study

- This study focuses on children accessing healthcare facilities, and findings indicate that vaccination screening and catch-up vaccination at the inpatient health facility level provides an important opportunity to improve vaccination coverage and protection against vaccine-preventable diseases.
- The proportion of under-vaccinated children was higher among hospitalized children at the two regions in India compared to those in the general population as indicated by regional vaccination coverage information.
- A high proportion of hospitalized children were missed for catch-up vaccination during their admission and hospital course despite the available opportunities to provide catch-up vaccination.
- Over half of the under-vaccinated children reported prior healthcare facility contact for a sick visit, indicating that these children were well within reach of the heathcare system.

For peer review only

Introduction

Although global vaccination rates have remained high over for the last ten years[1], more than one out of every three children were not fully vaccinated in India in 2014[2]. According to the 2015-2016 National Family Health Survey (NFHS-4), vaccination rates have improved steadily with each required vaccine coverage increasing between 8-23 percentage points in overall vaccination coverage in India's National Immunization Schedule over the past ten years [3-5]. A massive nationwide effort called the Mission Indradhanush strategy was launched by the Government of India in 2014, which vaccinated approximately 25.5 million vulnerable children in a two-year span [6]. Nonetheless, a joint report in 2016 conducted by the World Health Organization (WHO), United Nations, and Government of India Ministry of Health and Family Welfare concluded that estimated vaccination rates tended to be overestimated by administrative vaccination coverage reports and must still be improved to meet the WHO Sustainable Development Goal (SDG # 3) of reducing child mortality [7]. The report acknowledged the challenges faced by the government in identifying and addressing 'missed vaccination' opportunities for children [7]. WHO defines a missed opportunity for vaccination (MOV) as any contact with health services by a child or adult who is eligible for vaccination, which does not result in the individual receiving all the vaccine doses for which he or she is eligible [8].

Hospitalized children represent a vulnerable population with risk factors for poor health outcomes, who are more likely than the general population to be eligible for catch-up vaccinations [9]. Children admitted to healthcare facilities may be at greater risk for infectious diseases, especially vaccine-preventable illnesses [10]. These children can benefit if vaccination opportunities are identified and addressed while the child is in the hospital. An analysis using

For peer review only

Demographic Health Surveys (DHS) and Multiple Indicator Cluster Surveys (MICS) estimated that if children in contact with health services for acute care were to receive their due vaccines, the potential increase in DTP3 vaccination coverage would be 3-14% in low and middle income countries (LMIC) settings, including a 12% increase in India [10]. Our study aimed to document vaccination status of hospitalized children by obtaining vaccination cards where available, verifying the medical records at hospital admission and discharge, and assessing vaccination barriers perceived by caregivers and healthcare providers. Our findings will be useful for estimating the proportion of missed opportunities for vaccination among hospitalized children in India, and to understand local barriers for vaccination that can potentially form the basis for contextually effective interventions.

Methods

This study was a cross-sectional observational research study at two tertiary care government hospitals in Chandigarh and Jaipur conducted between November 2018 and March 2019. Children aged 1-59 months, were screened for study eligibility at admission to the hospitals. Those children admitted to the hospital, and whose parents agreed to provide vaccination information and consented to the study, were enrolled. Children who had health conditions that precluded the use of vaccines, such as a known primary or secondary immune deficiency, malignancy requiring chemotherapy, and children with previous reported allergies or contraindications to any vaccine or vaccine component, were excluded from enrollment. Following written informed consent from the caregiver, concerted efforts were made to obtain the vaccination card from parents during the hospital stay. If the card was not present, the parent or caregiver was requested to have a family member bring the card from their home or send a

For peer review only

picture of the card using their mobile phone. Parental recall was used to document vaccination status only if the vaccination card was unavailable.

At admission, caregivers were interviewed about demographic details, household information and prior health seeking behaviors. Children who had received all recommended vaccines for their age, per the prevalent national immunization schedule, were classified as ‘fully vaccinated’ [11]. Those who had received some, but not all, of the recommended vaccines for their age group, were considered ‘partially vaccinated’. Those who had received none of the recommended vaccines for their age were labelled ‘unvaccinated’. Research team members used study definitions and the recommended national immunization schedule to classify children who were unvaccinated or partially vaccinated as ‘under-vaccinated’ (Table 1). This classification was verified during the analysis phase using the child’s age at time of interview and reported vaccinations. No discrepancies existed between research staff evaluation and analysis. Under-vaccinated children were tracked until discharge to record if any vaccinations, or advice regarding vaccinations, had been given during hospitalization or at discharge. The medical chart was reviewed to document, which vaccines, if any, were administered during hospitalization. The caregivers of under-vaccinated children were interviewed at discharge to assess their perspectives on vaccination within the context of the hospital.

Table 1. Study definitions for vaccination status by age range

Age range (months)	Birth Dose	First Dose	Second Dose	Third Dose	Booster Dose
<1 month	BCG				
1 month [4-7 weeks]	BCG OPV	OPV OR f-IPV;			

For peer review only - http://bmjopen.bmj.com/site/about/guidelines.xhtml

		Penta OR DPT+Hep B			
2 months [8-11 weeks]	BCG OPV	OPV OR f-IPV; Penta OR DPT+Hep B	OPV OR f-IPV; Penta OR DPT+Hep B		
3 – 8 months	BCG OPV	OPV OR f-IPV; Penta OR DPT+Hep B	OPV OR f-IPV; Penta OR DPT+Hep B	OPV OR f-IPV; Penta OR DPT+Hep B	
9 – 15 months	BCG OPV	OPV OR f-IPV; Penta OR DPT+Hep B; Measles OR MMR OR MR	OPV OR f-IPV; Penta OR DPT+Hep B	OPV OR f-IPV; Penta OR DPT+Hep B	
16 – 59 months	BCG OPV	OPV OR f-IPV; Penta OR DPT+Hep B; Measles OR MMR OR MR	OPV OR f-IPV; Penta OR DPT+Hep B; Measles OR MMR OR MR	OPV OR f-IPV; Penta OR DPT+Hep B	OPV DPT

**Vaccination Status**	**Definition**
**Fully vaccinated**	Child has had all of the recommended vaccines for his/her age group as defined above
**Partially vaccinated**	Child has had at least one, but not all, of the recommended vaccines for his/her age group as defined above
**Unvaccinated**	Child has had none of the recommended vaccines for his/her age group as defined above

BCG: Bacille Calmette-Guerin vaccine; OPV: Oral poliovirus vaccine; f-IPV: Fractional dose of inactivated poliovirus vaccine; Penta: Diphtheria-tetanus-pertussis-hepatitis B-Haemophilus influenzae type b pentavalent vaccine; DPT: Diphtheria-tetanus-pertussis vaccine; Hep B: Hepatitis B vaccine; Measles: Measles vaccine; MMR: Measles-mumps-rubella vaccine; MR: Measles-rubella vaccine

After the enrollment period, surveys were distributed to hospital healthcare providers involved in children's admission, treatment and discharge. Providers were asked to anonymously share their professional experiences, vaccination practices and related policies of the hospital. Eligible

For peer review only

healthcare providers included physicians, nurses, pharmacy personnel, and postgraduate residents in the inpatient unit and the emergency unit. Questions were derived from the WHO *Planning Guide to Reduce Missed Opportunities for Vaccination* and WHO *Methodology for the Assessment of Missed Opportunities for Vaccination* [10, 12].

Ethical considerations

This study was approved by The Institutional Review Board at the Johns Hopkins Bloomberg School of Public Health and local Institutional Ethics Committees at the Postgraduate Institute of Medical Education and Research (PGIMER), Chandigarh and Sawai Man Singh (SMS) Medical College and attached hospitals, Jaipur. Written informed consent was taken from participants prior to enrollment and implementation of study procedures. All anonymized data was collected on paper and entered into REDCap.

Patient and public involvement

Prior to study initiation and during the development process, we piloted the study questionnaires to refine them further. We involved some caregivers of hospitalized children for this piloting purpose, and their feedback was used to re-design the data collection methods. This contribution is noted in the acknowledgement section. Patients and their caregivers were active participants in this research, but were not involved in the conduct, analysis or dissemination of this research.

Sample size calculations

The sample size calculations were conducted to enroll a total sample of 250 hospitalized eligible children and conduct an equal number of caregiver interviews. These sample size calculations

For peer review only

are based on the following assumptions: the estimated proportion of children admitted to the hospital who are unvaccinated or incompletely immunized is 50%, the estimated proportion of those eligible children who missed the opportunity to get vaccinated during their hospital visit is 30%, and the loss to follow up of children in the study between admission and discharge (discharged before the follow-up contact; or refused/was unable to participate) is 20% (alpha of 0.05, margin of error of ±9%).

Statistical analysis

Study outcomes included (i) the proportion of hospitalized children considered under-vaccinated for their age; (ii) the proportion of missed opportunities for vaccination among under-vaccinated children who were eligible for vaccination [8] and (iii) vaccine coverage by antigen. In addition, we compared these estimates of vaccine coverage among our sample of hospitalized children with regional Chandigarh and Jaipur estimates from the NFHS-4 published dataset [2]. For this sub-analysis to remain comparable with the NFHS-4 datasets, we only included children aged 12-23 months old using the NFHS-4 definition of 'fully vaccinated' who have received 1 dose of BCG, 3 doses each of polio and DPT+Hib+ Hep B (Penta) and 1 dose measles [13]. We fitted multivariable Poisson regression models for predictors of being under-vaccinated. Poisson regression is more appropriate than logistic regression due to the common (>10%) prevalence of being under-vaccinated and because the log binomial odds ratio models did not converge [14]. Weight-for-age z-scores were calculated using WHO growth indicator equations [15]. Caregiver and healthcare provider survey responses were coded and analyzed as binary outcomes for knowledge questions (correct/incorrect) and perception statements (agree/disagree). All

For peer review only

statistical analyses were conducted using Stata (Version 13.1, StataCorp, College Station, Texas, United States).

Results

Of the 312 admitted children who were screened for enrollment, 49 were ineligible (35 had no vaccination card or parental recall of vaccination details and 14 had a prior precluding health condition), leaving 263 (62 at Chandigarh and 201 at Jaipur) who consented and were subsequently enrolled into the study (Figure 1).

Participant characteristics

The median age of children in our sample was 7 months (range, 1 to 58 months), and 57.8% (152/263) were male. First-born children made up 45.2% (119/263) of our sample. The median age of caregivers in the study was 25 years (ranging from 18 to 63 years), and 78.6% (206/262) were female, with the majority of caregivers being mothers. The highest attained education level was high school or greater among 64.5% of caregivers, and 77.2% could read and write. The primary residential setting was rural among 60.8% (160/263), with 31.1% (80/263) of children living more than 5 kilometers away from their nearest health center (Table S1). At the time of this study, 92.4% (243/263) caregivers reported having a vaccination card for their children, however only 59.7% (157/263) were able to make this available at the hospital. Parental recall was used as the immunization source among the remaining 40.3% (106/263).(Table S1).

Vaccination status

For peer review only

Among the 263 children included in the final analysis, 34.6% (91) of the children were fully vaccinated while 65.4% (172) were under-vaccinated. Among under-vaccinated children, 60.1% (158/263) and 5.3% (14/263) were partially vaccinated and unvaccinated, respectively. The proportion of under-vaccinated children was highest at 76.6%, (59/77) among those under four months of age (Figure 2). The average hospital duration among those under-vaccinated was 5 days (ranging from 1 to 58 days), and 47% (72/161) were classified as underweight (weight < 2 z-scores below the mean age-for weight WHO definition) [15]. Prior health facility contact for a sick visit at any time since birth, and within the last month, was reported in 55.6% (93/167) and 40.7% (68/167) of under-vaccinated children, respectively.

The vaccinations with the lowest coverage among all included children were OPV3 (53.9%), DPT3 (54.9%), and DPT2 (63.1%). Our sample also had less than 80% vaccination coverage for the following vaccines: OPV0 (64.3%), OPV1 (69.2%), and OPV2 (64.4%) and MCV1 (79.7%). The coverage of Measles-containing-vaccine second-dose (MCV2) [Measles, MR, MMR], OPV booster dose and DPT booster dose were 40.9%, 56.8% and 59.1% respectively (Supplemental Table 2). Using the NHFS-4 study definition of full vaccination, our sample of 12-23 months old children had a proportion of 35.4% (17/48) fully vaccinated children, while the reported 2015-2016 estimated national average for full vaccination coverage was 62.0% [13]. Furthermore, our sample had lower coverage than the regional average for every vaccine, except BCG (90.1%) and MCV1 (79.7%) [2] (Table 2). A similar look at the two states separately showed similar results (Table S3).

For peer review only - http://bmjopen.bmj.com/site/about/guidelines.xhtml

Table 2. Vaccination coverage for individual vaccine antigens among hospitalized children at the study sites compared to national vaccination coverage rates from states (Chandigarh and Rajasthan NFHS-4)

	Vaccination coverage among study population of hospitalized children (n=263)			Aggregated Chandigarh and Rajasthan NFHS-4 estimates [2015-2016][1]
**Vaccine Dose**	**Vaccinated**	**Age Eligible**	**Coverage**	**Coverage[2]**
	**N**	**N**	**(%)**	**(%)**
BCG	237	263	90.1%	88.2%
OPV Dose 0	169	263	64.3%	71.7%
OPV Dose 1	182	263	69.2%	84.8%
OPV Dose 2	145	225	64.4%	78.2%
OPV Dose 3	110	204	53.9%	63.7%
DPT Dose 1 (DPT or Penta)	187	263	71.1%	83.6%
DPT Dose 2 (DPT or Penta)	142	225	63.1%	78.0%
DPT Dose 3 (DPT or Penta)	112	204	54.9%	69.6%
Measles 1 (Measles, MR, MMR)	55	69	79.7%	80.4%
Fully Vaccinated[3]	17	48	35.4%	62.0%

1 International Institute for Population Sciences (IIPS) and ICF. National Family Health Survey (NFHS-4) 2015-16 [Dataset]. Data Extract from IAIR72.SAV, IAKR72.SAV, IABR72.SAV, IAMR74.SAV, and IAPR74.SAV. IPUMS Demographic and Health Surveys (IPUMS DHS), 2018. [2]
2 Coverage is from birth to 48 months of age. age range in our sample was from 1-59 months of age
3 NFHS defines fully vaccinated as having BCG, measles, and 3 doses each of polio and DPT by 12-23 months of age.

Among 161 under-vaccinated children who had a medical chart available at discharge, 98.1% (158/161) represented a missed opportunity for vaccination. The most common admission diagnoses were, lower respiratory tract infection, sepsis, fever for evaluation, and epilepsy. Three children who expired during the study and 8 children who remained admitted in the hospital at study end (Figure 1) were excluded from the analysis. There was no significant difference of missed opportunities for vaccination by age. (Figure 2). Two children (aged 1 month and 17 months) were given their missed vaccinations in hospital, and a third child (aged 3 months) visited the immunization clinic for catch up vaccination prior to discharge. The discharge interview was completed among 88% (142/161) of caregivers of under-vaccinated children while the remaining 12% (19/161) were discharged outside of study hours. Among those interviewed,

For peer review only - http://bmjopen.bmj.com/site/about/guidelines.xhtml

62.9% of caregivers knew the correct purpose of vaccination (‘to prevent diseases’), 8.5% reported having their child’s vaccination history verified by staff, 1.4% reported receiving vaccine recommendations and information on vaccine clinic locations, 1.4% were told about the potential vaccination side effects, and 90.1% reported they were comfortable having their children vaccinated while they were sick. When asked about vaccine reminder services or text message reminders for vaccinations after discharge, 95% of caregivers said they would support such measures, and 32.9% suggested there should be more vaccination outreach services.

Predictors of being under-vaccinated

In univariate analysis, there was a significant association between being under-vaccinated and the following determinants: children aged less than 4 months compared to older children (PR 1.26, 95% CI 1.04, 1.49), parents with lower than high school educational attainment (PR 1.45, 95% CI 1.15, 1.84), children of mothers not involved in vaccination decisions (PR 1.23, 95% CI 1.02, 1.50), those previously vaccinated in a private health facility (PR 1.32, 95% CI 1.04, 1.67), and children whose vaccination information was collected through parental recall due to the lack of an available vaccination card, (PR 1.48, 95% CI 1.25, 1.76). In multivariable analyses, there were similar trends between the outcome of being under-vaccinated and the predictors, although there was no statistical significance (Table 3).

For peer review only

Table 3. Prevalence rate ratio [PRR] of being under vaccinated based on multiple predictors (N = 263)

	**Total**		**Under Vaccinated**		**Univariate Analysis**			**Multivariable Analysis[1]**		
	**N**	**(%)**	**N**	**(%)**	**PR**	**95% CI**	**p-value**	**aPR**	**95% CI**	**p-value**
Child Gender										
Female	111	42%	71	64%	referent			referent		
Male	152	58%	101	66%	1.03	(0.87 , 1.24)	0.68	0.97	(0.80 , 1.18)	0.78

Child Age										
Over Four Months	186	71%	113	61%	referent			referent		
Under Four Months	77	29%	59	77%	1.26	(1.06 , 1.49)	<0.01*	1.04	(0.83 , 1.29)	0.74
Child Birth Order										
First	119	45%	71	60%	referent			referent		
After (2nd, 3rd, 4th)	144	55%	101	70%	1.18	(0.98 , 1.41)	0.08	1.15	(0.93 , 1.42)	0.19
Residency										
Urban/Suburban	103	39%	61	59%	referent			referent		
Rural	160	61%	111	69%	1.17	(0.97 , 1.42)	0.10	1.14	(0.91 , 1.43)	0.26
Caregiver Education (262)[2]										
College or beyond college	85	32%	44	26%	referent			referent		
High school	84	32%	57	33%	1.31	(1.02 , 1.69)	0.04	1.3	(0.97 , 1.75)	0.08
Less than high school	93	35%	70	41%	1.45	(1.15 , 1.84)	<0.01*	1.29	(0.99 , 1.68)	0.06
Health Facility Contact (257 [2]										
Over three months	44	17%	25	15%	referent			referent		
Within last three months	94	37%	68	41%	1.27	(0.96 , 1.70)	0.10	1.13	(0.81 , 1.57)	0.49
Never	119	46%	74	44%	1.09	(0.82 , 1.47)	0.55	1.01	(0.73 , 1.40)	0.94
Health Facility Distance (257) [2]										
0 - 5 km	177	69%	111	63%	referent			referent		
5 - 50 km	80	31%	56	70%	1.12	(0.93 , 1.34)	0.24	0.99	(0.80 , 1.22)	0.92
Vaccination Decision Maker (257)[2]										
Mother involved	214	83%	133	62%	referent			referent		
Mother not involved	43	17%	33	77%	1.23	(1.02 , 1.50)	0.03*	1.16	(0.92 , 1.46)	0.22
Has Rotavirus Vaccine Dose 1										
Yes	74	28%	31	42%	referent			referent		
No	189	72%	141	75%	1.78	(1.34 , 2.36)	<0.01*	1.40	(1.01 , 1.95)	0.05
Prior Vaccination Setting (223) [2]										
Government	202	91%	124	61%	referent			referent		
Private	21	9%	17	81%	1.32	(1.04 , 1.67)	0.02*	1.35	(1.07 , 1.71)	0.02*
Have Their Vaccination Card										
Present at hospital	154	59%	84	55%	referent			referent		
Absent at hospital	109	41%	88	81%	1.48	(1.25 , 1.76)	<0.00	1.18	(0.94 , 1.47)	0.15

1 Adjusted for all variables in the table
2 Number in parenthesis refers to denominator in each category if different from the main denominator of 263.

Healthcare providers

Among 21 healthcare providers who participated across both sites (physicians and nurses from the pediatric inpatient units, emergency unit, and immunization clinic), 57% were female, the

median age was 27 years and 76.2% reported having more than one year of experience working in the healthcare field. Among the healthcare providers, 85.7% had incorrect knowledge of vaccination contraindications, listing low grade fever, pneumonia or diarrhea, and acute condition-related hospitalization as absolute contraindications. Although 95% agreed that it was appropriate to vaccinate a hospitalized child prior to discharge, 80% had concerns about vaccinating a child recovering from an acute illness, and 80% believed vaccines were not available in the inpatient unit. Reported barriers to vaccination of hospitalized children were, illness of the hospitalized child (71.4%), non-availability of immunization staff in the inpatient ward (71.4%), non-provision of vaccines in the inpatient ward (61.9%), and the focus of hospital health providers on management of acute illness rather than primary preventative measures (47.6%). Most (94.4%) believed these barriers could be addressed. Strategies suggested for improving catch-up vaccinations were: providing a reminder service at discharge for children missing vaccinations (85.7%), improving communication regarding vaccination status screening (76.2%), and improving vaccine delivery logistics and communication among hospital staff (66.7%).

Discussion

We observed a high proportion of under-vaccinated children among those hospitalized. This proportion was substantially higher than the national and regional averages for the period 2015-16 [2]. The overall proportion of missed opportunities for vaccination during hospitalization was 98.1%, which was significantly higher in comparison with other observational studies that included inpatient and outpatient healthcare facilities (25 - 43%) [9, 16-19]. A recent meta-analysis from seven low and middle income countries (LMICs) in Asia, Africa and the Americas

For peer review only

found a pooled prevalence of missed vaccination opportunities among children accessing healthcare facilities at 32% over a 22 year period [20]. An observational survey study in Mumbai found that healthcare providers missed opportunities to vaccinate children 80% of the time during a visit for an illness compared to only 0.7% of the time during a visit for a well child [21]. Using WHO standardized evaluation instruments, a study across 30 health facilities in Chad and Malawi found a higher proportion of missed vaccination opportunities in non-vaccination purpose visits (77%-92%), as seen in our study, compared to vaccination visits (47-51%) [22]. A descriptive analysis of private and public health facilities in four African countries found that 93-99% of eligible children remained under-vaccinated during outpatient non-vaccination, acute-care visits based on the provider's response [23]. In line with these studies, our findings suggest that reducing missed vaccination opportunities can be impactful during inpatient non-vaccination hospital visits.

We observed that vaccination coverage among our sample of hospitalized children was substantially lower than nationally reported and regional vaccination coverage estimates for infant vaccination in general. Hospitalized children accessing healthcare in the public sector, such as our study sites, usually come from lower income quintiles from outside the cities seeking tertiary care and are often the most vulnerable to repeated events of ill health [24]. Individuals in the lowest-income quintiles are particularly vulnerable to illness as they are at higher risk of being malnourished, living in crowded conditions, and having less access to clean water [25]. In addition to conditions that contribute to poor health outcomes, the poorest children in India are less likely to be fully vaccinated, leaving them even more susceptible to preventable diseases, and leaving their caregivers susceptible to catastrophic health costs. We found that children who

For peer review only

were in contact with a health facility in the last three months were less likely to be fully vaccinated, suggesting a continual pattern of missed opportunities for vaccination. It is important in chracterizing this association to understanding that parents may not routinely seek vaccines or be aware that their children are under-vaccinated, hence it is crucial for facilities to devote more attention to vaccination screening among admitted children. Addressing missed opportunities for vaccination is even more important considering the COVID-19 pandemic, and its negative impact on routine vaccine-seeking behavior [26]. Interventions and communication strategies will be particularly important, as this will facilitate easy catch-up for vaccines missed during the pandemic, and it will also ensure there is infrastructure in place to rollout an eventual COVID-19 vaccine.

In this study, some healthcare provider perceptions could be significant barriers to provision of vaccinations. A study in Chad and Malawi found that 92% and 88% of healthcare providers, respectively, were unable to correctly identify valid contraindications for vaccination [22]. Our observations on a limited number of healthcare provider perceptions indicate that interventions targeting vaccination catch-up should focus on facility barriers training on vaccination schedules and contraindications, encourage screening for childhood vaccination status upon admission, and ensure that staff at inpatient and sick visit areas as well as in immunization clinics are well informed of the need for routine and catch-up childhood vaccination services. In studies that investigated the determinants of vaccination, barriers have been generalized into categories that included health system barriers (cost, infrastructure); provider barriers (poor knowledge of contraindications, poor access to immunization records); and caregiver barriers (economic problems, poor understanding of side effects) [27,28]. As barriers to vaccination are unique

For peer review only

across regions, with varying determinants, understanding them at the caregiver and provider level can lead to the design of context-specific interventions to drive vaccine uptake in the inpatient setting. A systematic review of barriers to vaccination and missed opportunities found risk factors associated with being under-vaccinated which were similar to our findings [20]. This review additionally identified lower household socioeconomic status, late initiation of the child's vaccination, parental information gap, provider practices, and a variety of clinic factors as contributing factors [28]. The first 3 months of life is an intense period for vaccinations, and is also a vulnerable time for illnesses. More research is needed to further explore the association among these risk factors, as well as test interventions that bring together community and facility-based care to ensure complete vaccination of all children. The MOV strategy designed by the WHO describes the standardized global practice for reducing missed opportunities for vaccination, which includes implementing the interventions, providing supportive supervision and monitoring progress, conducting rapid field evaluations of outcome, and incorporating into the long term vaccination plans to ensure sustainable goals [29]. Implementing screening and communication strategies to reduce missed opportunities for vaccination at the facility level will be crucial and cost-effective for enhancing and optimising vaccination coverage.

This study was limited by a small sample size and was completed within a short duration. We included those without a vaccination card and depended on parental recall in some children, which may have affected reliability [30]. A systematic review on validity of parental recall for childhood vaccination surveys called into question the reliability of this method, but also concluded that excluding caregiver recall could result in considerable bias in vaccine coverage estimations [31]. We took considerable precautions to ensure that hospital healthcare providers

For peer review only

did not become greatly aware of the study details, and this minimized influencing their behavior during the study [32]. Caregiver persepctives were assessed only among those whose children were under-vaccinated, due to limitations in follow-up. Admission diagnoses were not routinely captured for the purpose of the study and hence are not reported in the analyses. Healthcare provider responses were not analyzed based on professional job title or years of experience, as the numbers were too small to show significant differences. This study was only conducted at two government hospitals, which could limit the generalizability of the results. In India, patients that receive medical care at government-run hospitals have lower out-of-pocket expenses compared to those accessing healthcare in the private sector, potentially biasing our sample based on facility selection [24]. While the two hospitals are in different states, there was no private hospital comparison in this preliminary study. However, we believe that focusing on government-run hospitals is a strength of our work, as it enabled us to characterize a population in India that is more prone to being under-vaccinated, while also allowing us to capture missed opportunities for vaccination among vulnerable populations [24, 28]. Moreover, the public sector is responsible for providing 60% of antenatal visits, as well as 90% of vaccination doses to children in India [24]. By conducting this research in the public sector, we were able to gain an understanding of missed opportunities, as well as barriers to vaccinations, in the setting where the vast majority of children are vaccinated. Interventions based on these insights have the potential to have a profound impact on vaccine uptake in India. This pilot study helped establish feasibility assessments and power calculations for an interventional trial for addressing missed opportunities for vaccination that has been initiated in India and Nigeria.

Conclusion

For peer review only

In conclusion, we found a high proportion of under-vaccination among hospitalized children, and the proportion of missed vaccination opportunities was high in these healthcare facilities. Our observation that a substantial proportion of under-vaccinated children have had prior contact with healthcare facilities underscores the need for interventions that repurpose existing resources at these healthcare facilities to ensure that vaccination opportunities are not missed. Addressing the system for vaccination screening at healthcare facilities, improved communication strategies targeted at both caregivers and healthcare providers and removing logistic barriers to vaccine access within the healthcare facility, can significantly improve vaccination inequities.

For peer review only

References

1 Peck M, Gacic-dobo M, Diallo MS, *et al.* Global Routine Vaccination Coverage, 2018. *MMWR* 2019;68:937–942. doi:http://dx.doi.org/10.15585/mmwr.mm6842a1

2 International Institute for Population Sciences (IIPS) and ICF. National Family Health Survey (NFHS-4) 2015-16 [Dataset]. Data Extract from IAIR72.SAV, IAKR72.SAV, IABR72.SAV, IAMR74.SAV, and IAPR74.SAV. IPUMS Demographic and Health Surveys (IPUMS DHS), 2018.

3 Ministries of Health and Family Welfare. National Family Health Survey - 4 State Fact Sheet NCT Delhi. 2015; 1–36. http://rchiips.org/NFHS/pdf/NFHS4/DL_FactSheet.pdf

4 Ministries of Health and Family Welfare. National Family Health Survey - 4 District Fact Sheet Jaipur Rajasthan. 2015. http://rchiips.org/nfhs/FCTS/RJ/RJ_FactSheet_110_Jaipur.pdf

5 Ministries of Health and Family Welfare. National Family Health Survey-4 State Fact Sheet Chandigarh. *Natl Fam Heal Surv – 4, Demogr Heal Surv* 2015. http://rchiips.org/nfhs/pdf/NFHS4/CH_FactSheet.pdf

6 Gurnani V, Haldar P, Aggarwal MK, *et al.* Improving vaccination coverage in India: lessons from Intensified Mission Indradhanush, a - cross-sectoral systems strengthening

For peer review only

strategy. *bmj* 2018;363:1–7. doi:10.1136/bmj.k4782

7 Bhatnagar P, Gupta S, Kumar R, *et al.* Estimation of child vaccination coverage at state and national levels in India. *Bulletin of the World Health Organization* 2016;94:728–34. doi:10.2471/BLT.15.167593

8 Bvudzai P, Joao G, Shendale S, *et al.* Reducing missed opportunities for vaccination in selected provinces of Mozambique : A study protocol. *Gates Open Research* 2017;1:1–11. doi:10.12688/gatesopenres.12761.1

9 Kumar D, Aggarwal A, Gomber S. Immunization Status of Children Admitted to a Tertiary-care Hospital of North India : Reasons for Partial Immunization or Non-immunization. *Journal of health, population, and nutrition* 2010;28:300–4. doi:10.3329/jhpn.v28i3.5560

10 World Health Organization. Planning Guide to Reduce Missed Opportunities for Vaccination (MOV). 2016;:1–60.

11 Ministries of Health and Family Welfare. National Immunization Schedule (NIS) National Immunization Schedule in India; 2017. 2017. http://www.ihatepsm.com/blog/national-immunization-schedule-india-2017

12 World Health Organization. Methodology for the Assessment of Missed Opportunities

For peer review only

for Vaccination. 2017;:1-72.

13 Ministries of Health and Family Welfare. National Family Health Survey - 4 India National Fact Sheet. *Natl Fam Heal Surv - 4* 2015.

14 Spiegelman D, Hertzmark E. Easy SAS Calculations for Risk or Prevalence Ratios and Differences. *Am J Epidemiol* 2005;162:199–200. doi:10.1093/aje/kwi188

15 World Health Organization. Training Course on Child Growth Assessment WHO Child Growth Standards. 2008;:7.

16 Garib Z, Vargas AL, Trumbo SP, *et al.* Missed Opportunities for Vaccination in the Dominican Republic : Results of an Operational Investigation. *BioMed research international* 2016. doi: https://doi.org/10.1155/2016/4721836

17 Tagbo B, Onwuasigwe C. Missed immunization opportunities among children in Enugu. *Niger J Paediatr* 2006;32:73-76

18 Deivanayagam N, Nedunchelian K, Mala N, *et al.* Missed opportunities for immunization in children under 2 years attending an urban teaching hospital. *Indian Pediatr* 1995;32:51–7.

19 Li AJ, Sudath T, Peiris R, *et al.* Opportunities to improve vaccination coverage in a

For peer review only

country with a fledgling health system : Findings from an assessment of missed opportunities for vaccination among health center attendees — Timor. *Vaccine* 2019;37:4281–90. doi:10.1016/j.vaccine.2019.06.041

20 Sridhar S, Maleq N, Guillermet E, *et al.* A systematic literature review of missed opportunities for immunization in low- and middle-income countries. *Vaccine* 2014;32:6870–9. doi:10.1016/j.vaccine.2014.10.063

21 Muranjan Mehta, C., Pakhare, A. M. An Observational, Health Service Based Survey for Missed Opportunities for Immunization. *Indian Pediatr* 2011;48:633–6.

22 Ogbuanu IU, Li AJ, Anya BM, *et al.* Can vaccination coverage be improved by reducing missed opportunities for vaccination ? Findings from assessments in Chad and Malawi using the new WHO methodology. 2019;14:1–19. doi:10.1371/journal.pone.0210648

23 Olorunsaiye CZ, Langhamer MS, Wallace AS, *et al.* Missed opportunities and barriers for vaccination : a descriptive analysis of private and public health facilities in four African countries. *Pan Afr Med J* 2017;27:1–9. doi:10.11604/pamj.supp.2017.27.3.12083

24 Mahal A, Yazbeck AS, Peters DH, *et al.* The Poor and Health Service Use in India. 2001. doi: https://doi.org/10.11588/xarep.00003863

25 Agarwal S, Satyavada A, Kaushik S, *et al.* Urbanization, Urban Poverty and Health of the

For peer review only

Urban Poor: Status, Challenges and the Way Forward. *Demography India* 2007;36:121–34.

26 Nelson R. COVID-19 disrupts vaccine delivery. *Lancet Infect Dis* 2020;20:546. doi:10.1016/S1473-3099(20)30304-2

27 Esposito S, Principi N, Cornaglia G, *et al.* Barriers to the vaccination of children and adolescents and possible solutions. *Clinical Microbiology and Infection* 2014;20:25-31. doi: https://doi.org/10.1111/1469-0691.12447

28 Santoli JM, Szilagyi PG, Rodewald LE. Barriers to immunization and missed opportunities. *Pediatr Ann* 1998;27:366–74. doi:10.3928/0090-4481-19980601-11

29 World Health Organization. Intervention Guidebook Intervention Guidebook for implementing and monitoring activities to reduce Missed Opportunities for Vaccination. 2019;:1-30

30 Miles M, Ryman TK, Dietz V, *et al.* Validity of vaccination cards and parental recall to estimate vaccination coverage: A systematic review of the literature. *Vaccine* 2013;31:1560–8. doi:10.1016/j.vaccine.2012.10.089

31 Modi RN, King C, Bar-Zeev N, *et al*. Caregiver recall in childhood vaccination surveys: Systematic review of recall quality and use in low- and middle-income settings. *Vaccine*

For peer review only

2018;36:4161-4170. doi: 10.1016/j.vaccine.2018.05.089

32 Leonard KL, Masatu MC. Using the Hawthorne effect to examine the gap between a doctor ' s best possible practice and actual performance. *J Dev Econ* 2010;93:226–34. doi:10.1016/j.jdeveco.2009.11.001

For peer review only

Contributorship Statement: NWA, JLM, and AS conceived the study with contributions from SS. NWA prepared the dataset and conducted the analysis of the data. NWA, BD, and AS prepared the first draft of the manuscript. NWA, RC, AT, and IB were responsible for overseeing the acquisition and management of the data for the study. All authors reviewed the drafts of this manuscript, provided critical input, and approved the final version for submission.

Acknowledgements: The authors thank Laura Lochlainn, Ikechukwu Udo, and Stephanie Shendale of the Department of Immunization, Vaccines & Biologicals, WHO headquarters (WHO-HQ) for early discussion and input on the study, and thank Farhad Khan for helpful discussions on the analysis and drafting of the manuscript. The authors are grateful to the caregivers of hospitalized children who assisted in refining and piloting of the study questionnaires prior to study initiation.

Funding: The MOVAHC study was funded through the Bill and Melinda Gates Foundation Gates Grand Challenges (OPP1217304).

Data sharing statement: Additional data can be made available upon request by emailing ashet1@jhu.edu

Figure Legends

Figure 1. Health-center–based flow-chart for determining eligibility. Missing vaccination history includes unable able to provide a vaccination card or accurately recall the vaccination history.

For peer review only

Figure 2. The two main study outcomes are shown: proportion under-vaccinated (in blue) and proportion who missed opportunities for vaccination while in the health facility (in green) by age range groupings (months). The number below the age range groupings on the x-axis show the number of children within each age range grouping. The age range groupings correspond to ages when doses are recommended by the National Immunization Schedule.

For peer review only

Figure 1. Enrollment flow-chart for the study

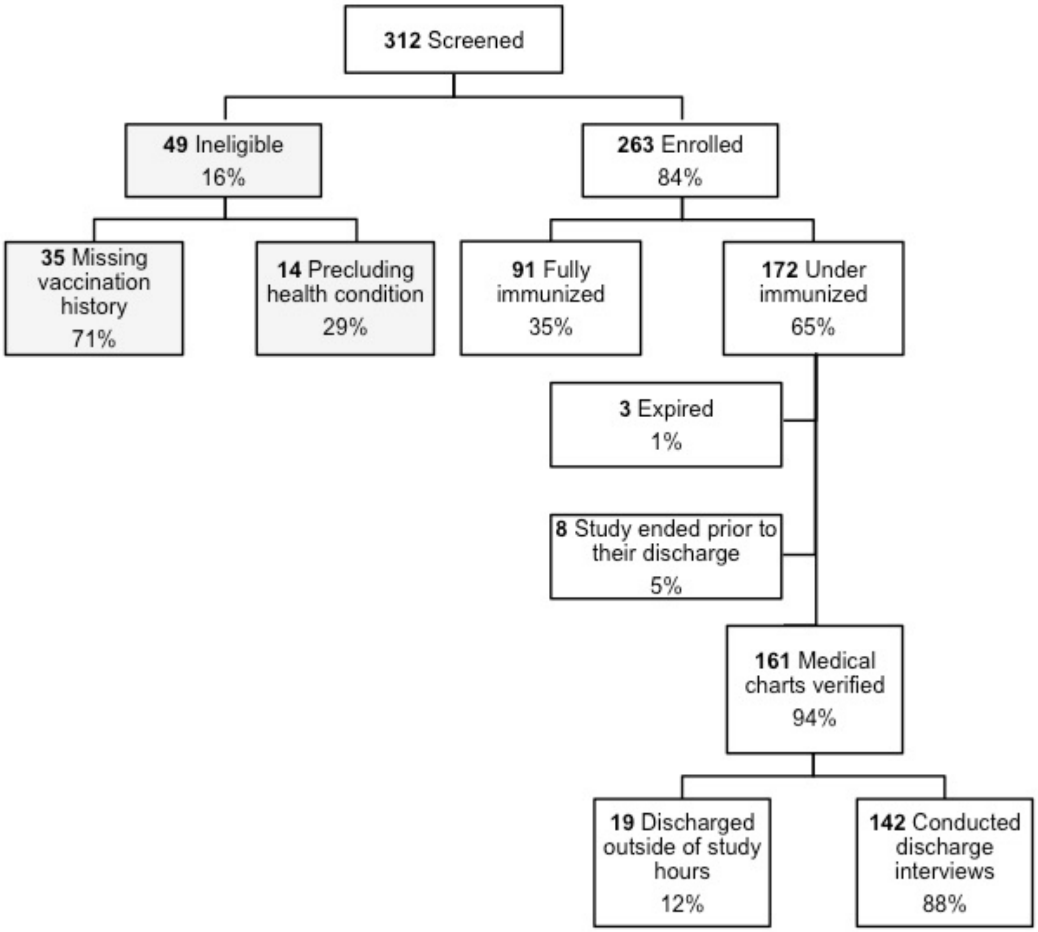

Figure 2. Proportion of children under vaccinated and missed oppurtunities for vaccination by age
n = 263

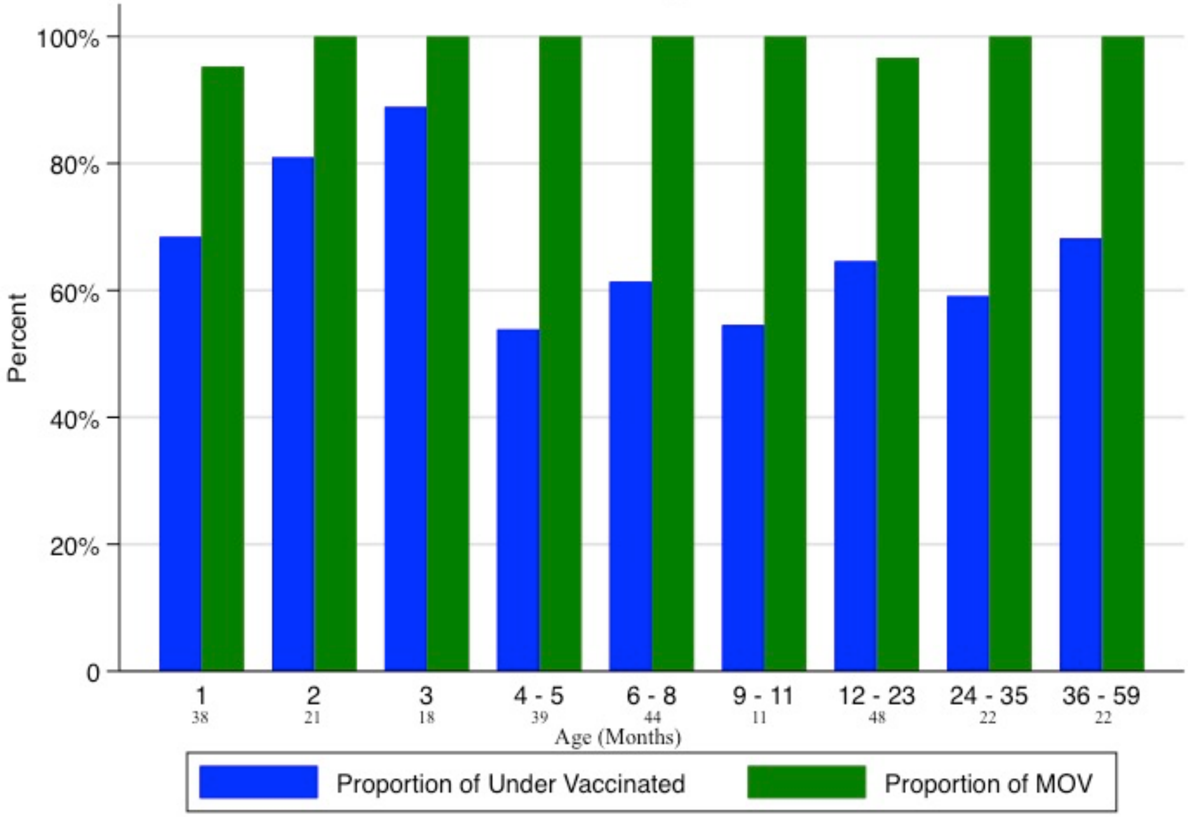

iew only

Supplementary Data

Table S1. Characteristics of enrolled children, their caregivers, and the household (N = 263)

	n	(%)
**Child Age (Months)**		
Minimum	1	
Mean	13	
Median	7	
Maximum	58	
**Child Gender**		
Male	152	57.8%
Female	111	42.2%
**Child Order**		
First-born	119	45.2%
Second-born	87	33.1%
Third-born	36	13.7%
Fourth-born or younger	21	8.0%
**Caregiver Age (Years)**		
Minimum	18	
Mean	27	
Median	25	
Maximum	63	
**Caregiver Gender[1]**		
Male	56	21.4%
Female	206	78.6%
**Relationship to Child[2]**		
Mother	192	73.3%
Father	50	19.1%
Aunt / Uncle	11	4.2%
Grandparent	9	3.4%
**Can caregiver read and write**		
Yes	203	77.2%
No	60	22.8%
**Caregiver level of education[3]**		
No formal education / did not complete primary	58	22.1%
Primary / middle school	35	13.4%
High School	84	32.1%
College and postgraduate	85	32.4%
**Household Religion**		
Hindu	205	77.9%
Muslim	46	17.5%

Sikh	12	4.6%
**Primary Residence**		
Urban	86	32.7%
Rural	160	60.8%
Suburban	17	6.5%
**Household Total**		
Minimum	3	
Mean	8	
Median	7	
Maximum	26	
**Child been in contact with another health center before today[4]**		
Yes	138	53.7%
No	119	46.3%
**When was child admitted to the other health center[5]** (denominator includes those with prior health center contact - 138)		
Within last month	66	47.8%
1 – 6 months	45	32.6%
Over 6 months	27	19.6%
**Makes the primary decisions on vaccination in the household[6]**		
Mother	142	55.3%
Father	19	7.4%
Consensus of father and mother	72	28.0%
Grandparent or other relative	24	9.3%
**Distance of this hospital (PGIMER or SMS) from residence[7]**		
0-5 km	24	9.3%
6-10 km	20	7.8%
11-50 km	26	10.1%
>50 km	188	72.9%
**Distance of any health facility from residence[8]**		
0-5 km	177	68.9%
6-10 km	60	23.3%
11-50 km	18	7.0%
>50 km	2	0.8%
**Child has a vaccination card**		
Yes, present at hospital	154	58.6%
Yes, absent at hospital	89	33.8%
No	20	7.6%
**Source of Immunization History**		
Immunization card	157	59.7%
Parental recall	106	40.3%
**Immunization Status**		
Fully Vaccinated	91	34.6%
Partially Vaccinated	158	60.1%

Unvaccinated	14	5.3%

[1] 1 respondent missing

[2] 1 respondent missing

[3] 1 respondent missing

[4] 6 respondents missing

[5] Denominator is 138 children in contact with another health facilty before today

[6] 6 respondents missing

[7] 5 respondents missing

[8] 6 respondents missing

For peer review only

Table S2. Additional vaccine coverage (N = 263)

Vaccine Dose	Sample Immunization Status		
	Vaccinated N	Age Eligible N	Coverage (%)
f-IPV[1] Dose 1	108	263	41.1%
f-IPV[1] Dose 2	52	204	25.5%
Rotavirus Dose 1	74	263	28.1%
Rotavirus Dose 2	55	225	24.4%
Rotavirus Dose 3	35	204	17.2%
Measles 2 (Measles, MR[2], MMR[3])	18	44	40.9%
OPV[4] Booster	25	44	56.8%
DPT[5] Booster	26	44	59.1%

[1] Fractional dose of inactivated poliovirus vaccine (f-IPV).

[2] Measles -rubella (MR) vaccine.

[3] Measles-mumps-rubella (MMR) vaccine.

[4] Oral poliovirus vaccine (OPV).

[5] Diphtheria-tetanus-pertussis (DPT) vaccine.

Table S3. Full vaccination coverage by site compared to state coverage rates (Rajasthan and Chandigarh, NFHS-4)

Fully vaccinated Jaipur study site		Fully vaccinated (Jaipur District, NFHS-4)		Fully vaccinated Chandigarh study site		Fully vaccinated (Chandigarh, NFHS-4)	
n	%	n[1]	%[1,2]	n	%	n[1]	%[1,2]
66	32.8%	5582	58.2	25	40.3	29	79.5

1 International Institute for Population Sciences (IIPS) and ICF. National Family Health Survey (NFHS-4) 2015-16 [Dataset]. Data Extract from IAIR72.SAV, IAKR72.SAV, IABR72.SAV, IAMR74.SAV, and IAPR74.SAV. IPUMS Demographic and Health Surveys (IPUMS DHS), 2018. [2]
2 Ministries of Health and Family Welfare. National Family Health Survey - 4 District Fact Sheet Jaipur Rajasthan. 2015. http://rchiips.org/nfhs/FCTS/RJ/RJ_FactSheet_110_Jaipur.pdf [4]
3 Ministries of Health and Family Welfare. National Family Health Survey-4 State Fact Sheet Chandigarh. *Natl Fam Heal Surv – 4, Demogr Heal Surv* 2015. http://rchiips.org/nfhs/pdf/NFHS4/CH_FactSheet.pdf [5]
